# Investigating causal associations among gut microbiota, metabolites, and liver diseases: a Mendelian randomization study

**DOI:** 10.3389/fendo.2023.1159148

**Published:** 2023-07-05

**Authors:** Lilong Zhang, Liuliu Zi, Tianrui Kuang, Kunpeng Wang, Zhendong Qiu, Zhongkai Wu, Li Liu, Rongqiang Liu, Peng Wang, Weixing Wang

**Affiliations:** ^1^ Department of General Surgery, Renmin Hospital of Wuhan University, Wuhan, Hubei, China; ^2^ Hubei Key Laboratory of Digestive System Disease, Wuhan, Hubei, China; ^3^ Central Laboratory, Renmin Hospital of Wuhan University, Wuhan, Hubei, China

**Keywords:** alcoholic liver disease, nonalcoholic fatty liver disease, viral hepatitis, gut microbiota, gut microbiota-derived metabolites, mendelian randomization analysis

## Abstract

**Objective:**

There is some evidence for an association between gut microbiota and nonalcoholic fatty liver disease (NAFLD), alcoholic liver disease (ALD), and viral hepatitis, but no studies have explored their causal relationship.

**Methods:**

Instrumental variables of the gut microbiota (N = 13266) and gut microbiota-derived metabolites (N = 7824) were acquired, and a Mendelian randomization study was performed to explore their influence on NAFLD (1483 European cases and 17,781 European controls), ALD (2513 European cases and 332,951 European controls), and viral hepatitis risk (1971 European cases and 340,528 European controls). The main method for examining causality is inverse variance weighting (IVW).

**Results:**

IVW results confirmed that *Anaerotruncus* (*p* = 0.0249), *Intestinimonas* (*p* = 0.0237), *Lachnoclostridium* (*p* = 0.0245), *Lachnospiraceae NC2004 group* (*p* = 0.0083), *Olsenella* (*p* = 0.0163), and *Peptococcus* (*p* = 0.0472) were protective factors for NAFLD, and *Ruminococcus 1* (*p* = 0.0120) was detrimental for NAFLD. The higher abundance of three genera, *Lachnospira* (*p* = 0.0388), *Desulfovibrio* (*p* = 0.0252), and *Ruminococcus torques group* (*p* = 0.0364), was correlated with a lower risk of ALD, while *Ruminococcaceae UCG 002* level was associated with a higher risk of ALD (*p* = 0.0371). The *Alistipes* (*p* = 0.0069) and *Ruminococcaceae NK4A214 group* (*p* = 0.0195) were related to a higher risk of viral hepatitis. Besides, alanine (*p* = 0.0076) and phenyllactate (*p* = 0.0100) were found to be negatively correlated with NAFLD, while stachydrine (O*p* = 0.0244) was found to be positively associated with NAFLD. The phenylacetate (*p* = 0.0353) and ursodeoxycholate (*p* = 0.0144) had a protective effect on ALD, while the threonate (*p* = 0.0370) exerted a detrimental influence on ALD. The IVW estimates of alanine (*p* = 0.0408) and cholate (*p* = 0.0293) showed their suggestive harmful effects against viral hepatitis, while threonate (*p* = 0.0401) displayed its suggestive protective effect against viral hepatitis.

**Conclusion:**

In conclusion, our research supported causal links between the gut microbiome and its metabolites and NAFLD, ALD, and viral hepatitis.

## 1 Introduction

Nonalcoholic fatty liver disease (NAFLD) is a prevailing form of chronic liver disease that is marked by the accumulation of hepatic fat in patients who do not have a history of heavy alcohol intake (1). It comprises a wide range of gradually deteriorating pathological disorders, ranging from a straightforward case of nonalcoholic fatty liver to a more serious case of nonalcoholic steatohepatitis (NASH), the latter of which has a higher risk of developing cirrhosis, organ failure, and hepatocellular carcinoma (2–4). Alcoholic liver disease (ALD) is a disease due to chronic and excessive alcohol intake. The accumulation of fat in the liver cells is one of the early responses to excessive alcohol use. When alcohol abuse persists, steatosis may develop into steatohepatitis, fibrosis, cirrhosis, and ultimately hepatocellular cancer (5). As an inflammation of the liver, hepatitis can either go away on its own or develop into a serious condition that results in cirrhosis or hepatocellular cancer. Globally, the main cause of hepatitis is viral, with hepatitis B and C virus infections usually developing into chronic hepatitis (6). There is an urgent need to identify potential causal risk factors for NAFLD, ALD, and viral hepatitis since they pose a significant health burden globally.

The gut microbiota, as the “forgotten organ”, is a dynamic and intricate community of ecological bacteria (7). The liver is the first organ crossed by the portal vein of the intestine. The phrase “gut-liver axis” was coined to describe the close connection between the intestinal flora, the immune system, and the intestinal barrier that occurs in the gut and liver (8). Through the portal vein, the liver gets 75% of its blood from the gut. By secreting bile and other mediators, it also gives the intestines feedback (9). Thus, various gut factors, such as gut microbiota, bacterial composition, and gut microbiota-derived metabolites, are deeply involved in the homeostasis of the liver.

Recently, there has been growing evidence that intestinal flora is closely related to human health and is involved in the etiology of various complex diseases, including liver diseases (9, 10). However, there is controversy among these studies. For example, Zhu et al. revealed a higher relative abundance of *Prevotella* and no distinct alternation in *Bacteroides* in NAFLD patients than the control (11). However, Boursier et al. found that, compared to healthy controls, patients with NASH had higher levels of *Bacteroides* and lower levels of *Prevotella* (12). Besides, when compared to controls, several studies have demonstrated an increase in the *Firmicutes* to *Bacteroidetes* ratio in NAFLD and NASH (13, 14), while others have shown a decrease in this ratio (11, 15, 16). Confounding or reverse causation in observational studies could be to blame for the contradictory results in gut microbial dysbiosis in NAFLD.

As we know, confounding factors and reverse causation may both affect the findings of current observational epidemiological research, making causal inference difficult. The Mendelian randomization (MR) method using genetic variants as instrumental variables (IVs) in the epidemiological investigation has been generally accepted to estimate the causal influence of exposure on diseases (17). Based on the Mendelian inheritance rule, parental genetic alleles are randomly dispersed to their offspring during the meiotic process, which is regarded as a randomized controlled study (RCT). This method was chosen because it was practical, economical, and less likely to be confounded by covariables (18). Also, since genetic variants are already set at the time of conception, MR is less susceptible to the influence of reverse causation. Previous genetic research has shown that host genetic variants can affect the intestinal flora, allowing us to explore the relationship between gut microbiota and liver diseases using the MR approach.

Thus, in this study, the summary data from genome-wide association studies (GWASs) was used to explore the causal association of gut microbiota and metabolites with NAFLD, ALD, and viral hepatitis using the two-sample MR analysis.

## 2 Materials and methods

### 2.1 Study design

**Figure 1:**
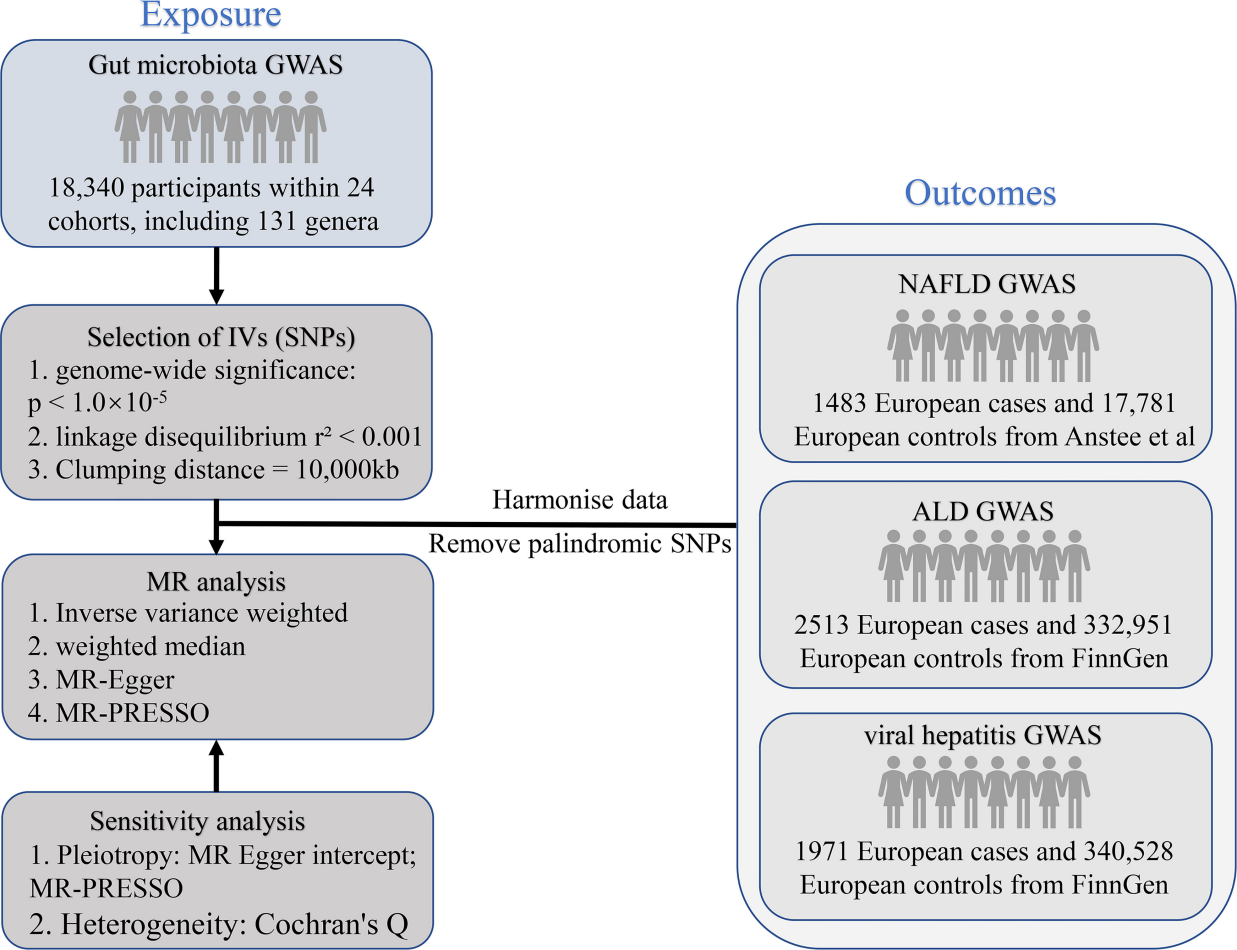
The study design of the present MR study of the associations of gut microbiota and metabolites and liver diseases. MR, Mendelian randomization; SNP, single nucleotide polymorphism; NAFLD, non-alcoholic fatty liver disease; ALD, alcoholic liver disease; GWAS, genome-wide association study; IV, instrumental variables.

MR analysis is a genetic method that infers the causal effects of exposure on outcomes by using the random allocation of genetic variants at conception. The SNPs employed as IVs need to meet the following basic assumptions. First, there has to be a solid association between the SNPs and the exposure; second, the SNPs should not be related to the outcome *via* confounders; and third, the SNPs should not impact the outcome directly. Earlier research detailed further particulars of this method (19). The STROBE-MR guidelines were used to design this research (20). **Figure 1** shows the flowchart of the MR study between gut microbiota and metabolites with liver diseases.

### 2.2 Exposure sources

Genetic instruments of intestinal microbiome were acquired from the largest genome-wide meta-analysis published by the MiBioGen consortium (21). The study contained 24 cohorts with 18,340 individuals, most of whom were of European ancestry (16 cohorts, N = 13,266). The study targeted variable regions V4, V3–V4, and V1–V2 of the 16S rRNA gene to profile the microbial composition and to conduct taxonomic classification using direct taxonomic binning. For each cohort, microbiota quantitative trait loci (mbQTL) mapping analysis included only the taxa presented in > 10% of the samples (21). The lowest taxonomic level in this study was genus, and 131 genera with a mean abundance > 1% were found, including 12 unknown genera (21). Thus, 119 genus-level taxa were obtained in our study for MR analysis. The included cohorts all made adjustments for sex and age as covariates in their calculations (21).

We also used summary-level data from the human metabolome GWAS performed among subjects of European descent (TwinsUK and KORA, N = 7824) in light of the significant roles gut metabolites play in microbiota-host interaction (22). Then we utilized HMDB (23) to acquire a list of 12 gut microbiota-derived metabolite traits from all the measured metabolites in the GWAS, such as betaine, carnitine, cholate, choline, alanine, phenylacetate, phenyllactate, stachydrine, threonate, and ursodeoxycholate.

### 2.3 Outcome sources

The genetic association with NAFLD was extracted from the newly published GWAS summary statistics by Anstee et al., consisting of 1483 European cases and 17,781 European controls (24). The top 5 genetic principal components and genotyping batch were corrected during the analysis (24). GWAS summary-level data for ALD (2513 European cases and 332,951 European controls) and viral hepatitis (1971 European cases and 340,528 European controls) were downloaded from FinnGen consortium R8 release data (25). During the analysis, age, sex, the first 10 principal components, and the genotyping batch were corrected (25).

### 2.4 Genetic instrument selection

To satisfy the above MR assumption, we selected IVs with linkage disequilibrium r² < 0.001 and distance > 10,000 kb and attaining genome-wide significance (*p* < 1.0×10^-5^) (26). The linkage disequilibrium reference panel was established utilizing the 1000 Genomes Project European sample (27). Each IV’s strength was determined utilizing the F statistics = beta^2^/se^2^ (28). For adequate strength to be determined, the F-statistics had to be >10.

### 2.5 Statistical analysis

The primary statistical analysis method was the inverse variance weighted (IVW) method under random effects. This method was supplemented with weighted median analysis (29), MR-Egger regression (30), and MR-PRESSO methods (31). IVW assumes that all genetic variation SNPs are valid IVs with an overall bias of zero. As for the weighted median analysis, this estimate is consistent even if up to half of the weights are from invalid instruments. Besides, MR-Egger analysis can identify horizontal pleiotropy through the intercept (*p* < 0.05 for the intercept indicates pleiotropy) (30). The MR-PRESSO method can detect possible outliers and generate causal estimates after the removal of outlying IVs (31). To measure the degree of heterogeneity, the Q-value from Cochrane was applied. The causal relationship is considered significant if: 1) the *p*-value of the IVW method is less than < 0.05; 2) the estimations obtained using the MR-Egger, weighted median, and IVW methods all have the same direction; and 3) neither the MR-Egger intercept test nor the MR-PRESSO global test has statistical significance (*p* > 0.05) (32). Furthermore, in addition to meeting the 3 conditions mentioned above, for the connection between gut microbiota or metabolites and liver diseases, a Bonferroni-adjusted IVW *p* (*p*FDR) value of 4.2 × 10^-5^ (*p* = 0.05/119) or 5 × 10^-4^ (P = 0.05/10) was employed as the cut-off for statistical significance. *p* < 0.05 but more than the Bonferroni corrected significance level was seen as suggestive of evidence for a potential association (33, 34). Each test was two-sided and conducted utilizing the TwoSampleMR and MR-PRESSO packages in the R software (version 4.2.1) (31, 35).

## 3 Results

### 3.1 Causal effect of gut microbiota on NAFLD

**Figure 2:**
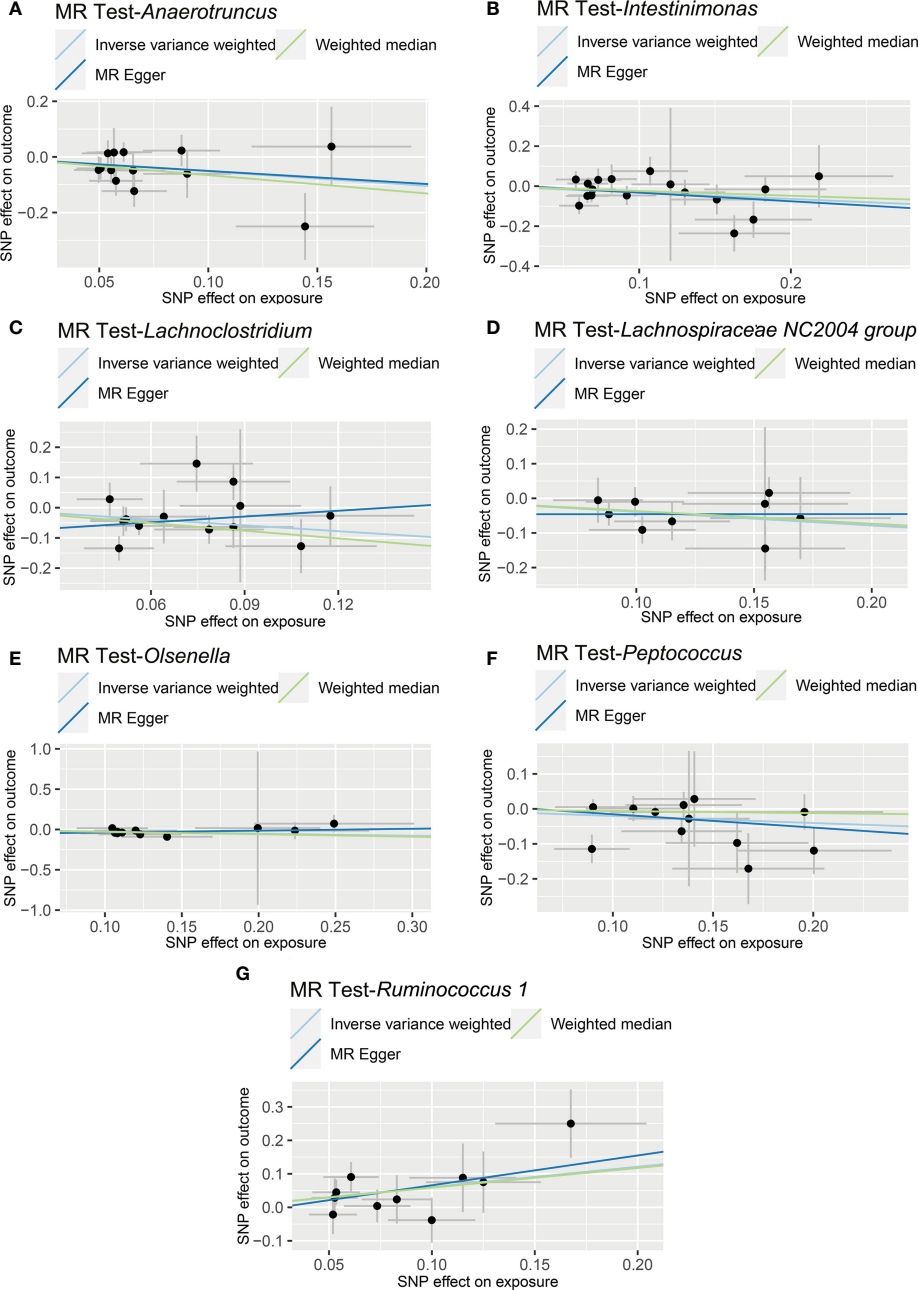
Causal relationship between gut microbiota and the risk of non-alcoholic fatty liver disease. Each point represents the SNP effects on *Anaerotruncus* **(A)**, *Intestinimonas* **(B)**, *Lachnoclostridium* **(C)**, *Lachnospiraceae NC2004 group* **(D)**, *Olsenella* **(E)**, *Peptococcus* **(F)**, *Ruminococcus 1* **(G)** and *non-alcoholic fatty liver disease*. MR, Mendelian randomization; SNP, single nucleotide polymorphism.

The results of IVW analyses demonstrated that *Anaerotruncus* (OR = 0.595, 95% CI: 0.378-0.937, *p* = 0.0249), *Intestinimonas* (OR = 0.726, 95% CI: 0.550-0.958, *p* = 0.0237), *Lachnoclostridium* (OR = 0.523, 95% CI: 0.297-0.920, *p* = 0.0245), *Lachnospiraceae NC2004 group* (OR = 0.676, 95% CI: 0.505-0.904, *p* = 0.0083), *Olsenella* (OR = 0.770, 95% CI: 0.623-0.953, *p* = 0.0163), and *Peptococcus* (OR = 0.817, 95% CI: 0.669-0.998, *p* = 0.0472) were negatively associated with NAFLD (**Table 1** and **Figure 2**), indicating a protective impact of the above genera on NAFLD (**Table 1**). The results of IVW analyses revealed that *Ruminococcus 1* (OR = 1.833, 95% CI: 1.142-2.940, *p* = 0.0120) was positively related to NAFLD, suggesting a detrimental effect on NAFLD (**Table 1** and **Figure 2**). These associations were also supported by the MR-PRESSO method, as shown in **Table 1**. Besides, the MR estimates of the weighted median analysis showed similar results in *Anaerotruncus* (OR = 0.519, 95% CI: 0.273-0.985, *p* = 0.0449), *Lachnoclostridium* (OR = 0.429, 95% CI: 0.225-0.816, *p* = 0.0099), and *Olsenella* (OR = 0.743, 95% CI: 0.562-0.982, *p* = 0.0370) (**Table 1** and **Figure 2**). Whereas, for *Lachnoclostridium* and *Olsenella*, MR-Egger analysis results were in the opposite direction to IVW and weighted median analysis results (**Table 1** and **Figure 2**). Detailed statistics for the remaining genera are shown in **Table S1**. We do not find significant heterogeneity across these results using Cochrane Q statistics (**Table 1**). The estimation of the intercept that was generated from the MR-Egger regression was centered around 0 and did not offer definitive evidence of horizontal pleiotropy (**Table 1**). No outliers were found by MR-PRESSO. The average F-statistic was 21.386, ranging from 17.045 to 28.784, revealing that there was no weak IV bias (**Table S2**).

**Table 1:** Association of genetically predicted gut microbiota with non-alcoholic fatty liver disease.

Methods	IVs	OR	95% CI	*p* value	Egger intercept, *p* value	Heterogeneity (Q, *p* value)	MR-PRESSO (Global test *p* value)
Anaerotruncus							
IVW	13	0.595	0.378-0.937	**0.0249**	-0.003, 0.952	11.377, 0.497	0.494
Weighted median	13	0.519	0.273-0.985	**0.0449**			
MR-Egger	13	0.625	0.119-3.301	0.5914			
MR-PRESSO	13	0.595	0.382-0.926	**0.0400**			
Intestinimonas							
IVW	17	0.726	0.550-0.958	**0.0237**	0.010, 0.761	18.312, 0.306	0.337
Weighted median	17	0.787	0.544-1.140	0.2049			
MR-Egger	17	0.651	0.308-1.376	0.2785			
MR-PRESSO	17	0.726	0.550-0.958	**0.0380**			
Lachnoclostridium							
IVW	13	0.523	0.297-0.920	**0.0245**	-0.087, 0.234	18.982, 0.089	0.107
Weighted median	13	0.429	0.225-0.816	**0.0099**			
MR-Egger	13	1.893	0.237-15.130	0.5593			
MR-PRESSO	13	0.523	0.297-0.920	**0.0441**			
Lachnospiraceae NC2004 group							
IVW	9	0.676	0.505-0.904	**0.0083**	-0.046, 0.542	7.121, 0.524	0.464
Weighted median	9	0.694	0.455-1.058	0.0891			
MR-Egger	9	0.998	0.290-3.466	0.9967			
MR-PRESSO	9	0.676	0.514-0.889	**0.0233**			
Olsenella							
IVW	11	0.770	0.623-0.953	**0.0163**	-0.060, 0.405	4.987, 0.892	0.897
Weighted median	11	0.743	0.562-0.982	**0.0370**			
MR-Egger	11	1.257	0.410-3.854	0.6979			
MR-PRESSO	11	0.770	0.663-0.895	**0.0067**			
Peptococcus							
IVW	12	0.817	0.669-0.998	**0.0472**	0.023, 0.690	14.159, 0.224	0.265
Weighted median	12	0.941	0.736-1.203	0.6284			
MR-Egger	12	0.683	0.283-1.646	0.4156			
MR-PRESSO	12	0.817	0.669-0.998	**0.0472**			
Ruminococcus 1							
IVW	10	1.833	1.142-2.940	**0.0120**	-0.023, 0.670	7.555, 0.580	0.897
Weighted median	10	1.800	0.920-3.523	0.0862			
MR-Egger	10	2.435	0.635-9.332	0.2305			
MR-PRESSO	10	1.833	1.188-2.826	**0.0228**			

IV, instrumental variables; OR, Odd Ratio; IVW, inverse variance weighted; Bold represents p < 0.05.

### 3.2 Causal effect of gut microbiota on ALD

**Figure 3:**
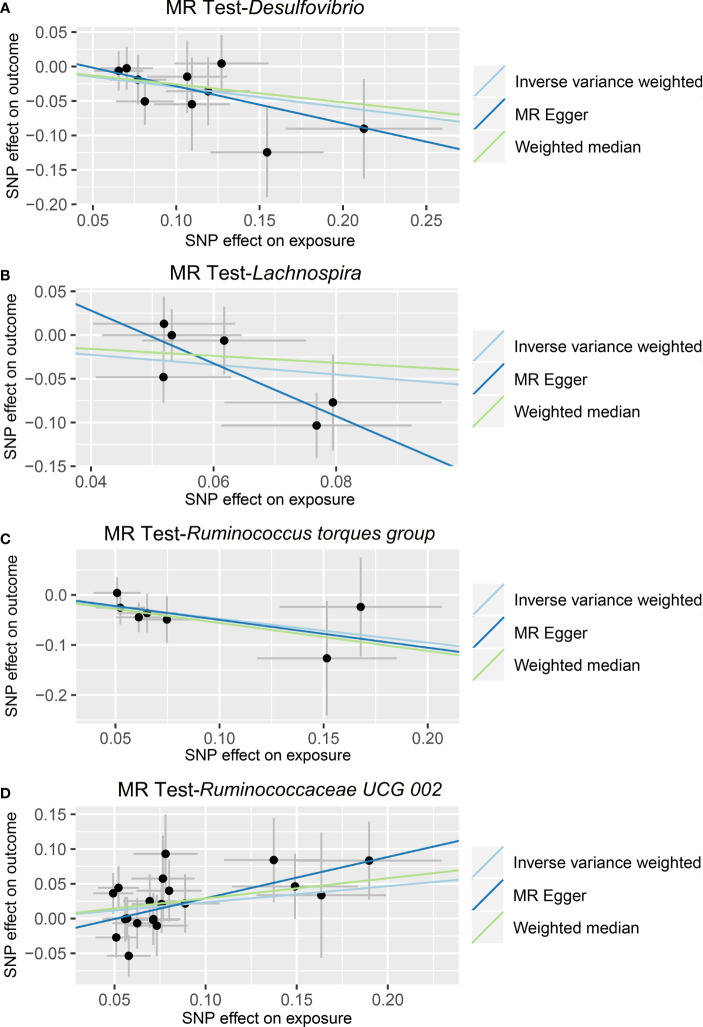
Causal relationship between gut microbiota and the risk of alcoholic liver disease. Each point represents the SNP effects on *Desulfovibrio* **(A)**, *Lachnospira* **(B)**, *Ruminococcus torques group* **(C)**, *Ruminococcaceae UCG 002* **(D)**, and alcoholic liver disease. MR, Mendelian randomization; SNP, single nucleotide polymorphism.

In the IVW method, we found that the genetically predicted higher relative abundance of three genera, *Lachnospira* (OR = 0.568, 95% CI: 0.332-0.971, *p* = 0.0388), *Desulfovibrio* (OR = 0.744, 95% CI: 0.574-0.964, *p* = 0.0252), and *Ruminococcus torques group* (OR = 0.621, 95% CI: 0.398-0.970, *p* = 0.0364), was correlated with a lower risk of ALD (**Table 2** and **Figure 3**); while, the genetically predicted *Ruminococcaceae UCG 002* level was associated with a higher risk of ALD (OR = 1.263, 95% CI: 1.014-1.572, *p* = 0.0371) (**Table 2** and **Figure 3**). The results of the MR-PRESSO analysis were similar to those of the IVW method (**Table 2**). The IVW test, weighted median method, and MR-Egger test were all in the same direction, which strengthened the confidence in the true causal associations. Detailed statistics for the remaining genera are shown in **Table S3**. No significant heterogeneity was observed across these results (**Table 2**). MR-Egger regression confirmed that there was no horizontal pleiotropy between IVs and outcomes (**Table 2**). Moreover, neither outliers nor any indication of pleiotropy were observed in the MR-PRESSO analysis (**Table 2**). The F-statistics of IVs ranged between 18.53 and 31.28, indicating no evidence of weak instrument bias (**Table S4**).

**Table 2:** Association of genetically predicted gut microbiota with alcoholic liver disease.

Methods	IVs	OR	95% CI	*p* value	Egger intercept, *p* value	Heterogeneity (Q, *p* value)	MR-PRESSO (Global test *p* value)
Ruminococcaceae UCG 002							
IVW	20	1.263	1.014-1.572	**0.0371**	-0.030, 0.196	13.997, 0.784	0.797
Weighted median	20	1.337	0.980-1.824	0.0670			
MR-Egger	20	1.812	1.024-3.206	0.0561			
MR-PRESSO	20	1.263	1.046-1.524	**0.0252**			
Lachnospira							
IVW	6	0.568	0.332-0.971	**0.0388**	0.149, 0.148	6.680, 0.246	0.278
Weighted median	6	0.672	0.355-1.273	0.2227			
MR-Egger	6	0.049	0.003-0.745	0.0956			
MR-PRESSO	6	0.568	0.332-0.971	**0.0454**			
Desulfovibrio							
IVW	10	0.744	0.574-0.964	**0.0252**	0.025, 0.532	4.043, 0.906	0.907
Weighted median	10	0.772	0.553-1.077	0.1272			
MR-Egger	10	0.584	0.271-1.260	0.2076			
MR-PRESSO	10	0.744	0.625-0.885	**0.0087**			
Ruminococcus torques group							
IVW	7	0.621	0.398-0.970	**0.0364**	0.006, 0.910	1.726, 0.943	0.945
Weighted median	7	0.571	0.319-1.022	0.0592			
MR-Egger	7	0.574	0.146-2.259	0.4633			
MR-PRESSO	7	0.621	0.489-0.789	**0.0080**			

IV, instrumental variables; OR, Odd Ratio; IVW, inverse variance weighted; Bold represents p < 0.05.

### 3.3 Causal effect of gut microbiota on viral hepatitis

**Figure 4:**
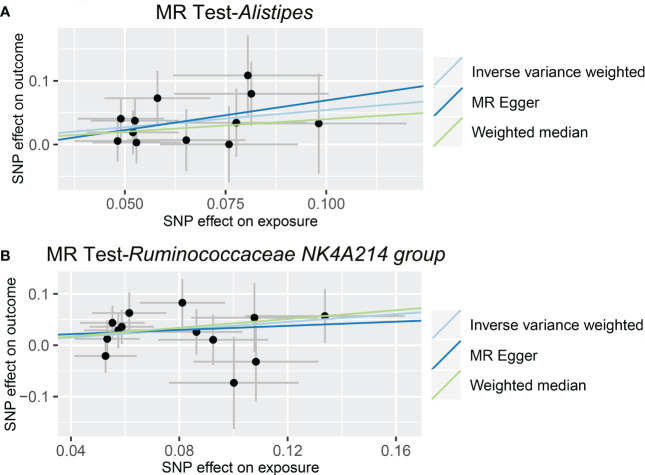
Causal relationship between gut microbiota and the risk of viral hepatitis. Each point represents the SNP effects on *Alistipes* **(A)**, *Ruminococcaceae NK4A214 group* **(B)**, and viral hepatitis. MR, Mendelian randomization; SNP, single nucleotide polymorphism.

As shown in **Figure 4** and **Table 3**, we observed that *Alistipes* (OR = 1.720, 95% CI: 1.160-2.550, *p* = 0.0069) and *Ruminococcaceae NK4A214 group* (OR = 1.460, 95% CI: 1.063-2.006, *p* = 0.0195) were related to a higher risk of viral hepatitis. The results of the MR-PRESSO analysis supported the above findings. Detailed statistics for the remaining genera are shown in **Table S5**. None of the MR-Egger regression intercepts deviated from null, and no outliers were detected with the MR-PRESSO test, suggesting no evidence of horizontal pleiotropy (**Table 3**). Besides, the F statistic was larger than 10, and the Cochrane Q statistic results revealed no significant heterogeneity (**Tables 3**, **S6**).

**Table 3:** Association of genetically predicted gut microbiota with viral hepatitis.

Methods	IVs	OR	95% CI	*p* value	Egger intercept, *p* value	Heterogeneity (Q, *p* value)	MR-PRESSO (Global test *p* value)
Alistipes							
IVW	12	1.720	1.160-2.550	**0.0069**	-0.024, 0.686	4.704, 0.920	0.951
Weighted median	12	1.490	0.891-2.494	0.1288			
MR-Egger	12	2.538	0.390-16.521	0.3527			
MR-PRESSO	12	1.720	1.330-2.225	**0.0017**			
Ruminococcaceae NK4A214 group							
IVW	13	1.460	1.063-2.006	**0.0195**	0.014, 0.735	7.206, 0.844	0.869
Weighted median	13	1.531	1.004-2.335	**0.0479**			
MR-Egger	13	1.224	0.429-3.493	0.7134			
MR-PRESSO	13	1.460	1.142-1.868	**0.0108**			

IV, instrumental variables; OR, Odd Ratio; IVW, inverse variance weighted; Bold represents p < 0.05.

### 3.4 Causal effect of gut microbiota-derived metabolites on liver diseases

**Figure 5:**
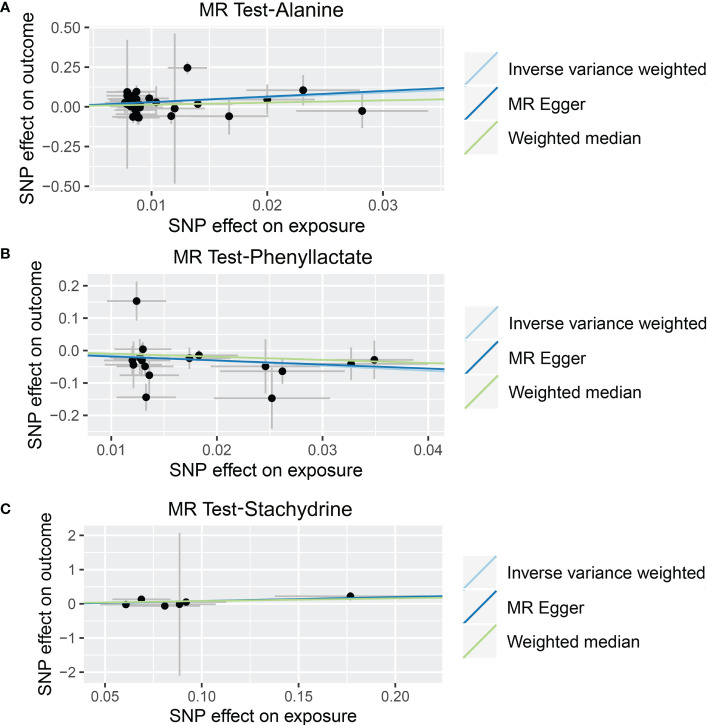
Causal relationship between gut microbiota-derived metabolites and the risk of non-alcoholic fatty liver disease. Each point represents the SNP effects on Alanine **(A)**, Phenyllactate **(B)**, Stachydrine **(C)**, and non-alcoholic fatty liver disease. MR, Mendelian randomization; SNP, single nucleotide polymorphism.

**Figure 6:**
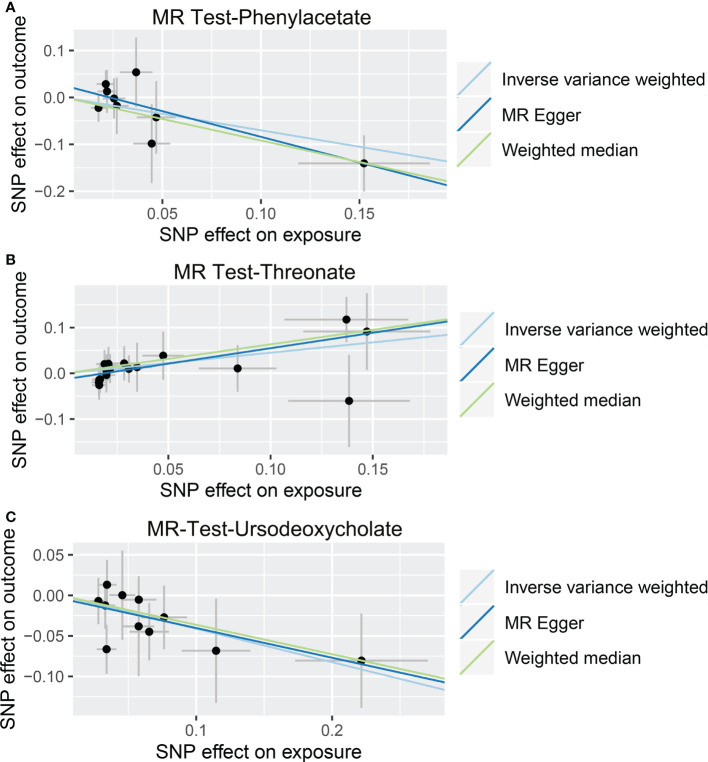
Causal relationship between gut microbiota-derived metabolites and the risk of alcoholic liver disease. Each point represents the SNP effects on Phenylacetate **(A)**, Threonate **(B)**, Ursodeoxycholate **(C)**, and alcoholic liver disease. MR, Mendelian randomization; SNP, single nucleotide polymorphism.

**Figure 7:**
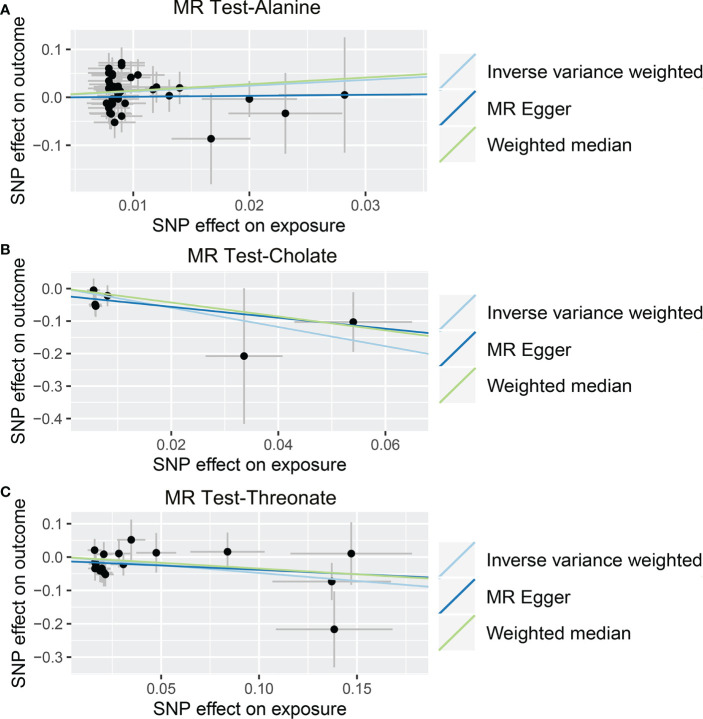
Causal relationship between gut microbiota-derived metabolites and the risk of viral hepatitis. Each point represents the SNP effects on Alanine **(A)**, Cholate **(B)**, Threonate **(C)**, and viral hepatitis. MR, Mendelian randomization; SNP, single nucleotide polymorphism.

As shown in **Tables 4**, **S7**, and **Figure 5**, alanine (OR = 19.586, 95% CI: 2.206-173.934, *p* = 0.0076) and phenyllactate (OR = 0.212, 95% CI: 0.065-0.689, *p* = 0.0100) were found to be negatively correlated with NAFLD, while stachydrine (OR = 2.228, 95% CI: 1.109-4.474, *p* = 0.0244) was found to be positively associated with NAFLD in the IVW and MR-PRESSO methods. The IVW estimate indicated that phenylacetate (OR = 0.496, 95% CI: 0.258-0.953, *p* = 0.0353) and ursodeoxycholate (OR = 0.662, 95% CI: 0.476-0.921, *p* = 0.0144) had a protective effect on ALD; while threonate (OR = 1.570, 95% CI: 1.028-2.397, *p* = 0.0370) exerts a detrimental influence on ALD (**Tables 5**, **S8**, and **Figure 6**). Besides, the IVW estimate of alanine (OR = 3.348, 95% CI: 1.052-10.655, *p* = 0.0408) and cholate (OR = 1.560, 95% CI: 1.046-2.327, *p* = 0.0293) showed its suggestive harmful effect against viral hepatitis; and threonate (OR = 0.621, 95% CI: 0.385-0.971, *p* = 0.0401) displayed its suggestive protective effect against viral hepatitis (**Tables 6**, **S9**, and **Figure 7**). No heterogeneity and horizontal pleiotropy were observed in these analyses (**Tables 4**–**6**). The F-statistics of IVs ranged between 17.64 and 88.97 (**Tables S10**–**12**).

**Table 4:** Association of genetically predicted gut microbiota derived metabolites with non-alcoholic fatty liver disease.

Methods	IVs	OR	95% CI	*p* value	Egger intercept, *p* value	Heterogeneity (Q, *p* value)	MR-PRESSO (Global test *p* value)
Alanine							
IVW	33	19.586	2.206-173.934	**0.0076**	-0.005 0.896	66.480, 0.001	0.067
Weighted median	33	3.814	0.289-50.406	0.3095			
MR-Egger	33	33.147	0.010-110573	0.4041			
MR-PRESSO	33	19.584	2.206-173.934	**0.0118**			
Phenyllactate							
IVW	17	0.212	0.065-0.689	**0.0100**	-0.006, 0.873	22.769, 0.120	
Weighted median	17	0.384	0.093-1.588	0.1863			0.183
MR-Egger	17	0.289	0.005-15.218	0.5486			
MR-PRESSO	17	0.212	0.065-0.689	**0.0203**			
Stachydrine							
IVW	6	2.228	1.109-4.474	**0.0244**	-0.052, 0.237	13.221, 0.104	
Weighted median	6	2.211	0.898-5.444	0.0843			0.454
MR-Egger	6	3.148	0.228-43.387	0.4398			
MR-PRESSO	6	2.228	1.109-4.474	**0.0342**			

IV, instrumental variables; OR, Odd Ratio; IVW, inverse variance weighted; Bold represents p < 0.05.

**Table 5:** Association of genetically predicted gut microbiota derived metabolites with alcoholic liver disease.

Methods	IVs	OR	95% CI	*p* value	Egger intercept, *p* value	Heterogeneity (Q, *p* value)	MR-PRESSO (Global test *p* value)
Phenylacetate							
IVW	9	0.496	0.258-0.953	**0.0353**	0.025, 0.274	4.928, 0.765	0.656
Weighted median	9	0.399	0.166-0.958	**0.0399**			
MR-Egger	9	0.335	0.133-0.841	0.0526			
MR-PRESSO	9	0.496	0.297-0.828	**0.0278**			
Threonate							
IVW	18	1.570	1.028-2.397	**0.0370**	-0.013, 0.333	6.562, 0.989	
Weighted median	18	1.885	1.018-3.490	**0.0436**			0.972
MR-Egger	18	1.965	1.066-3.619	**0.0457**			
MR-PRESSO	18	1.570	1.206-2.042	**0.0037**			
Ursodeoxycholate							
IVW	11	0.662	0.476-0.921	**0.0144**	-0.004, 0.851	4.814, 0.903	
Weighted median	11	0.696	0.425-1.137	0.1480			0.931
MR-Egger	11	0.693	0.392-1.224	0.2381			
MR-PRESSO	11	0.662	0.527-0.833	**0.0055**			

IV, instrumental variables; OR, Odd Ratio; IVW, inverse variance weighted; Bold represents p < 0.05.

**Table 6:** Association of genetically predicted gut microbiota derived metabolites with viral hepatitis.

Methods	IVs	OR	95% CI	*p* value	Egger intercept, *p* value	Heterogeneity (Q, *p* value)	MR-PRESSO (Global test *p* value)
Alanine							
IVW	37	3.348	1.052-10.655	**0.0408**	0.021, 0.323	31.059, 0.703	0.719
Weighted median	37	3.948	0.783-19.904	0.0962			
MR-Egger	37	1.098	0.005-29.948	0.6788			
MR-PRESSO	37	3.348	1.142-9.812	**0.0341**			
Cholate							
IVW	9	1.560	1.046-2.327	**0.0293**	-0.023, 0.475	14.834, 0.062	
Weighted median	9	1.291	0.798-2.086	0.2976			0.135
MR-Egger	9	1.989	0.936-4.223	0.1168			
MR-PRESSO	9	1.560	1.046-2.327	**0.0410**			
Threonate							
IVW	18	0.621	0.385-0.971	**0.0401**	-0.012, 0.406	10.826, 0.865	
Weighted median	18	0.709	0.346-1.453	0.3472			0.883
MR-Egger	18	0.769	0.387-1.528	0.4648			
MR-PRESSO	18	0.621	0.424-0.908	**0.0249**			

IV, instrumental variables; OR, Odd Ratio; IVW, inverse variance weighted; Bold represents p < 0.05.

## 4 Discussion

According to our knowledge, this study is the first to estimate the causal relationships between gut microbiota, gut microbiota-derived metabolites, and liver diseases using MR analysis. Our results revealed that multiple gut microbiota and metabolites play significant roles in the development of liver diseases, 5 suggestive microbial taxa (*Anaerotruncus*, *Intestinimonas*, *Lachnospiraceae NC2004 group*, *Peptococcus*, and *Ruminococcus 1*) and 3 suggestive metabolites (*Alanine*, *Phenyllactate*, and *Stachydrine*) in NAFLD, 4 suggestive microbial taxa (*Ruminococcaceae UCG 002*, *Lachnospira*, *Desulfovibrio*, and *Ruminococcus torques group*) and 3 suggestive metabolites (*Phenylacetate*, *Threonate*, and *Ursodeoxycholate*) in ALD, 2 suggestive microbial taxa (*Alistipes* and *Ruminococcaceae NK4A214 group*) and 3 suggestive metabolites (*Alanine*, *Cholate*, and *Threonate*) in viral hepatitis. Notably, the MR test *p* values for both gut microbiota and metabolites and liver diseases were greater than *p*FDR.

*Anaerotruncus* and *Intestinimonas* were revealed to be butyrate-producing bacterium in the intestine (36–39). *Intestinimonas* is generally recognized as beneficial bacteria with anti-inflammatory and anti-obesity properties (40). Rodriguez-Diaz et al. (41) found a significant decrease in the abundance of *Intestinimonas* in patients with NAFLD compared to the healthy population. Supplementation with Adzuki beans has been shown to significantly reduce high-fat diet-induced obesity and lipid accumulation, as well as lipopolysaccharide levels, and alleviate liver function impairment and hepatic steatosis (42). Besides, it significantly reversed the imbalance of gut microbiota caused by high-fat diets and significantly increased the abundance of *Lachnoclostridium* (42). As for *Olsenella*, Zhong et al. showed that probiotic-fermented blueberry juice significantly reduced low-density lipoprotein cholesterol levels and fat accumulation, ameliorated insulin resistance, and improved the abundance and diversity of intestinal microbial communities in high-fat diet mice (43). The blueberry juice-treated mouse showed a relatively high abundance of lean bacteria (*Olsenella* and *Bifidobacterium*) and a lower abundance of obesity-associated bacteria (*Oscillibacter* and *Alistipes*) compared to the high-fat diet-fed mouse (43). Interestingly, Li et al. revealed that the gut formation of propionic acid and acetic acid is related to an increase in *Olsenella* in pectin-fed mice (44). Recently, Pan et al. diagnosed 21 chronic hepatitis B and 42 NAFLD patients with the classic damp-heat (DH) syndrome group and identified 29 chronic hepatitis B and 28 NAFLD patients as the non-DH syndrome group. They found a decreased relative abundance of the *Lachnospiraceae NC2004 group* in patients with the DH syndrome compared to the non-DH syndrome (45). Taken together, these studies were in agreement with our MR analysis that this aforementioned genus plays a protective role in NAFLD. In contrast, Pung et al. demonstrated that Ulva prolifera polysaccharide greatly slowed high-fat diet-induced weight gain, ameliorated metabolic disturbances in high-fat diet-fed mice, and improved intestinal flora disorders, as evidenced by the growth in *Bifidobacterium* abundance and downregulation of *Ruminococcus 1* abundance (46). This implies that *Ruminococcus 1* may play a negative role in NAFLD.

*Alistipes* is mainly found in the intestines of healthy humans (47, 48). However, *Alistipes* has also been isolated from the bloodstream, appendiceal, and abdominal, highlighting its possible opportunistic pathogenic involvement in human disorders (48). Feng et al. found that *Alistipes* could promote the development of colorectal cancer *via* the interleukin-6/signal transducer and activator of transcription 3 pathway (49). As for *Ruminococcaceae UCG 002*, there was significant enrichment of *Ruminococcaceae UCG 002* abundance in prostate cancer patients compared to the healthy population, suggesting a pathogenic role (50). These studies support our conclusions. We found that *Ruminococcaceae UCG 002* and *Alistipes* play a pathogenic role in ALD and viral hepatitis, respectively.

*Lachnospira* was significantly lower in all disease cohorts (multiple sclerosis, inflammatory bowel disease, and rheumatoid arthritis) relative to healthy controls (51). Due to its reduced abundance, studies suggest that *Lachnospira* may have a protective effect under inflammatory conditions (51, 52). *Desulfovibrio* was negatively related to the host body mass index, waist, triglyceride, and uric acid, which are signs of obesity or metabolic disorders (53–56). The abundance of *Desulfovibrio* was positively related to the diversity of flora, favoring microbiome stability and host health (57, 58). Besides, *Desulfovibrio* was positively correlated with the beneficial bacteria *Oscillospira*, *Phascolarctobacterium*, *Prevotella*, *Coprococcus*, *Dialister*, *Ruminococcus*, *Akkermansia*, *Roseburia*, *Faecalibacterium*, and *Bacteroides* and negatively correlated with the harmful bacteria *Streptococcus*, *Clostridium*, *Escherichia*, *Klebsiella*, and *Ralstonia* (59–68). Previous studies have shown a positive correlation between the *Ruminococcus torques group* and short-chain fatty acid levels by studying some people who ingested less starch in order to lose weight (69). Recently, Wan et al. found that improvement in colitis was associated with a higher *Ruminococcus torques group*, suggesting that the *Ruminococcus torques group* may have another application as a potential probiotic in the anti-inflammatory response (70). The above studies revealed their beneficial role in human diseases and supported our findings.

This work also has some limitations. First, because only people of European heritage were included in the GWAS, the conclusions of this study might not apply to people of other racial or ethnic backgrounds. Second, the sequencing of the 16S rRNA genes only permitted resolution from the genus to the phylum level, not at a more specific level, and the results were skewed when certain specific species affected the risk of liver diseases. Third, our results are not significant after the Bonferroni adjustment. However, multiple statistical corrections may overlook GM taxa with a potential causal connection to liver diseases because they are excessively tight and cautious. Furthermore, although the Mendelian randomization analysis was comparable to the level of evidence from the RCT study, further animal experimental confirmation is necessary.

## 5 Conclusion

In conclusion, our research supported causal links between the gut microbiome and its metabolites and NAFLD, ALD, and viral hepatitis. It is necessary to conduct further population-based research on the potential mechanisms of gut microbiota and liver disease development.

## Data availability statement

The original contributions presented in the study are included in the article/**Supplementary Material**. Further inquiries can be directed to the corresponding authors.

## Ethics statement

Ethical review and approval was not required for the study on human participants in accordance with the local legislation and institutional requirements. Written informed consent for participation was not required for this study in accordance with the national legislation and the institutional requirements.

## Author contributions

LilZ, LiuZ, TK, PW, RL, and WW conceived and designed the study. LilZ, LiuZ, TK, ZQ, KW, ZW, and LL were responsible for the collection and assembly of data, data analysis, interpretation, and writing the manuscript. RL, PW, and WW revised the manuscript. All the work was performed under RL, PW, and WW instructions. All authors contributed to the article and approved the submitted version.

## References

[1] Younossi ZM Koenig AB Abdelatif D Fazel Y Henry L Wymer M. Global epidemiology of nonalcoholic fatty liver disease-meta-analytic assessment of prevalence, incidence, and outcomes. Hepatology (2016) 64(1):73–84. doi: 10.1002/hep.28431 26707365

[2] Hardy T Oakley F Anstee QM Day CP. Nonalcoholic fatty liver disease: Pathogenesis and disease spectrum. Annu Rev Pathol (2016) 11:451–96. doi: 10.1146/annurev-pathol-012615-044224 26980160

[3] Marjot T Moolla A Cobbold JF Hodson L Tomlinson JW. Nonalcoholic fatty liver disease in adults: Current concepts in etiology, outcomes, and management. Endocr Rev (2020) 41(1):bnz009. doi: 10.1210/endrev/bnz009 31629366

[4] Yuan Q Deng D Pan C Ren J Wei T Wu Z. Integration of transcriptomics, proteomics, and metabolomics data to reveal HER2-associated metabolic heterogeneity in gastric cancer with response to immunotherapy and neoadjuvant chemotherapy. Front Immunol (2022) 13:951137. doi: 10.3389/fimmu.2022.951137 35990657 PMC9389544

[5] Osna NA Donohue TM Jr. Kharbanda KK. Alcoholic liver disease: Pathogenesis and current management. Alcohol Res (2017) 38(2):147–61. 10.35946/arcr.v38.2.01 PMC5513682 28988570

[6] Ikeda K Saitoh S Suzuki Y Kobayashi M Tsubota A Koida I. Disease progression and hepatocellular carcinogenesis in patients with chronic viral hepatitis: a prospective observation of 2215 patients. J Hepatol (1998) 28(6):930–8. doi: 10.1016/s0168-8278(98)80339-5 9672166

[7] O'Hara AM Shanahan F. The gut flora as a forgotten organ. EMBO Rep (2006) 7(7):688–93. doi: 10.1038/sj.embor.7400731 PMC1500832 16819463

[8] Bruneau A Hundertmark J Guillot A Tacke F. Molecular and cellular mediators of the gut-liver axis in the progression of liver diseases. Front Med (Lausanne) (2021) 8:725390. doi: 10.3389/fmed.2021.725390 34650994 PMC8505679

[9] Manzoor R Ahmed W Afify N Memon M Yasin M Memon H. Trust your gut: The association of gut microbiota and liver disease. Microorganisms (2022) 10(5):1045. doi: 10.3390/microorganisms10051045 35630487 PMC9146349

[10] Xu M Luo K Li J Li Y Zhang Y Yuan Z. Role of intestinal microbes in chronic liver diseases. Int J Mol Sci (2022) 23(20):12661. doi: 10.3390/ijms232012661 36293518 PMC9603943

[11] Zhu L Baker SS Gill C Liu W Alkhouri R Baker RD. Characterization of gut microbiomes in nonalcoholic steatohepatitis (NASH) patients: a connection between endogenous alcohol and NASH. Hepatology (2013) 57(2):601–9. doi: 10.1002/hep.26093 23055155

[12] Boursier J Mueller O Barret M Machado M Fizanne L Araujo-Perez F. The severity of nonalcoholic fatty liver disease is associated with gut dysbiosis and shift in the metabolic function of the gut microbiota. Hepatology (2016) 63(3):764–75. doi: 10.1002/hep.28356 PMC4975935 26600078

[13] Del Chierico F Nobili V Vernocchi P Russo A De Stefanis C Gnani D. Gut microbiota profiling of pediatric nonalcoholic fatty liver disease and obese patients unveiled by an integrated meta-omics-based approach. Hepatology (2017) 65(2):451–64. doi: 10.1002/hep.28572 27028797

[14] Monga Kravetz A Testerman T Galuppo B Graf J Pierpont B Siebel S. Effect of gut microbiota and PNPLA3 rs738409 variant on nonalcoholic fatty liver disease (NAFLD) in obese youth. J Clin Endocrinol Metab (2020) 105(10):e3575–85. doi: 10.1210/clinem/dgaa382 PMC7458486 32561908

[15] Jasirwan COM Lesmana CRA Hasan I Sulaiman AS Gani RA. The role of gut microbiota in non-alcoholic fatty liver disease: pathways of mechanisms. Biosci Microbiota Food Health (2019) 38(3):81–8. doi: 10.12938/bmfh.18-032 PMC6663510 31384519

[16] Wong VW Tse CH Lam TT Wong GL Chim AM Chu WC. Molecular characterization of the fecal microbiota in patients with nonalcoholic steatohepatitis–a longitudinal study. PloS One (2013) 8(4):e62885. doi: 10.1371/journal.pone.0062885 23638162 PMC3636208

[17] Emdin CA Khera AV Kathiresan S. Mendelian randomization. JAMA (2017) 318(19):1925–6. doi: 10.1001/jama.2017.17219 29164242

[18] Paternoster L Tilling K Davey Smith G. Genetic epidemiology and mendelian randomization for informing disease therapeutics: Conceptual and methodological challenges. PloS Genet (2017) 13(10):e1006944. doi: 10.1371/journal.pgen.1006944 28981501 PMC5628782

[19] Dimou NL Tsilidis KK. A primer in mendelian randomization methodology with a focus on utilizing published summary association data. Methods Mol Biol (2018) 1793:211–30. doi: 10.1007/978-1-4939-7868-7_13 29876899

[20] Skrivankova VW Richmond RC Woolf BAR Davies NM Swanson SA VanderWeele TJ. Strengthening the reporting of observational studies in epidemiology using mendelian randomisation (STROBE-MR): explanation and elaboration. BMJ (2021) 375:n2233. doi: 10.1136/bmj.n2233 34702754 PMC8546498

[21] Kurilshikov A Medina-Gomez C Bacigalupe R Radjabzadeh D Wang J Demirkan A. Large-scale association analyses identify host factors influencing human gut microbiome composition. Nat Genet (2021) 53(2):156–65. doi: 10.1038/s41588-020-00763-1 PMC8515199 33462485

[22] Ning J Huang SY Chen SD Zhang YR Huang YY Yu JT. Investigating casual associations among gut microbiota, metabolites, and neurodegenerative diseases: A mendelian randomization study. J Alzheimers Dis (2022) 87(1):211–22. doi: 10.3233/JAD-215411 35275534

[23] Wishart DS Feunang YD Marcu A Guo AC Liang K Vazquez-Fresno R. HMDB 4.0: the human metabolome database for 2018. Nucleic Acids Res (2018) 46(D1):D608–D17. doi: 10.1093/nar/gkx1089 PMC5753273 29140435

[24] Anstee QM Darlay R Cockell S Meroni M Govaere O Tiniakos D. Genome-wide association study of non-alcoholic fatty liver and steatohepatitis in a histologically characterised cohort. J Hepatol (2020) 73(3):505–15. doi: 10.1016/j.jhep.2020.04.003 32298765

[25] Kurki MI Karjalainen J Palta P Sipilä TP Kristiansson K Donner K. FinnGen: Unique genetic insights from combining isolated population and national health register data. medRxiv (2022) 2022.03.03.22271360. doi: 10.1101/2022.03.03.22271360

[26] Sanna S van Zuydam NR Mahajan A Kurilshikov A Vich Vila A Vosa U. Causal relationships among the gut microbiome, short-chain fatty acids and metabolic diseases. Nat Genet (2019) 51(4):600–5. doi: 10.1038/s41588-019-0350-x PMC6441384 30778224

[27] Clarke L Zheng-Bradley X Smith R Kulesha E Xiao C Toneva I. The 1000 genomes project: data management and community access. Nat Methods (2012) 9(5):459–62. doi: 10.1038/nmeth.1974 PMC3340611 22543379

[28] Xie J Huang H Liu Z Li Y Yu C Xu L. The associations between modifiable risk factors and nonalcoholic fatty liver disease: A comprehensive mendelian randomization study. Hepatology (2023) 77(3):949–64. doi: 10.1002/hep.32728 35971878

[29] Bowden J Davey Smith G Haycock PC Burgess S. Consistent estimation in mendelian randomization with some invalid instruments using a weighted median estimator. Genet Epidemiol (2016) 40(4):304–14. doi: 10.1002/gepi.21965 PMC4849733 27061298

[30] Bowden J Davey Smith G Burgess S. Mendelian randomization with invalid instruments: effect estimation and bias detection through egger regression. Int J Epidemiol (2015) 44(2):512–25. doi: 10.1093/ije/dyv080 PMC4469799 26050253

[31] Verbanck M Chen CY Neale B Do R. Detection of widespread horizontal pleiotropy in causal relationships inferred from mendelian randomization between complex traits and diseases. Nat Genet (2018) 50(5):693–8. doi: 10.1038/s41588-018-0099-7 PMC6083837 29686387

[32] Zhang Y Liu Z Choudhury T Cornelis MC Liu W. Habitual coffee intake and risk for nonalcoholic fatty liver disease: a two-sample mendelian randomization study. Eur J Nutr (2021) 60(4):1761–7. doi: 10.1007/s00394-020-02369-z PMC7910323 32856188

[33] Yu XH Yang YQ Cao RR Bo L Lei SF. The causal role of gut microbiota in development of osteoarthritis. Osteoarthritis Cartilage (2021) 29(12):1741–50. doi: 10.1016/j.joca.2021.08.003 34425228

[34] Zhuang Z Li N Wang J Yang R Wang W Liu Z. GWAS-associated bacteria and their metabolites appear to be causally related to the development of inflammatory bowel disease. Eur J Clin Nutr (2022) 76(7):1024–30. doi: 10.1038/s41430-022-01074-w 35046568

[35] Hemani G Zheng J Elsworth B Wade KH Haberland V Baird D. The MR-base platform supports systematic causal inference across the human phenome. Elife (2018) 7:e34408. doi: 10.7554/eLife.34408 29846171 PMC5976434

[36] Bailen M Bressa C Martinez-Lopez S Gonzalez-Soltero R Montalvo Lominchar MG San Juan C. Microbiota features associated with a high-Fat/Low-Fiber diet in healthy adults. Front Nutr (2020) 7:583608. doi: 10.3389/fnut.2020.583608 33392236 PMC7775391

[37] Wang J Ji H Wang S Liu H Zhang W Zhang D. Probiotic lactobacillus plantarum promotes intestinal barrier function by strengthening the epithelium and modulating gut microbiota. Front Microbiol (2018) 9:1953. doi: 10.3389/fmicb.2018.01953 30197632 PMC6117384

[38] Luo L Hu M Li Y Chen Y Zhang S Chen J. Association between metabolic profile and microbiomic changes in rats with functional dyspepsia. RSC Adv (2018) 8(36):20166–81. doi: 10.1039/c8ra01432a PMC9080732 35541663

[39] Bui TP Ritari J Boeren S de Waard P Plugge CM de Vos WM. Production of butyrate from lysine and the amadori product fructoselysine by a human gut commensal. Nat Commun (2015) 6:10062. doi: 10.1038/ncomms10062 26620920 PMC4697335

[40] Cai W Xu J Li G Liu T Guo X Wang H. Ethanol extract of propolis prevents high-fat diet-induced insulin resistance and obesity in association with modulation of gut microbiota in mice. Food Res Int (2020) 130:108939. doi: 10.1016/j.foodres.2019.108939 32156386

[41] Rodriguez-Diaz C Taminiau B Garcia-Garcia A Cueto A Robles-Diaz M Ortega-Alonso A. Microbiota diversity in nonalcoholic fatty liver disease and in drug-induced liver injury. Pharmacol Res (2022) 182:106348. doi: 10.1016/j.phrs.2022.106348 35817360

[42] Zhao Q Hou D Fu Y Xue Y Guan X Shen Q. Adzuki bean alleviates obesity and insulin resistance induced by a high-fat diet and modulates gut microbiota in mice. Nutrients (2021) 13(9):3240. doi: 10.3390/nu13093240 34579118 PMC8466346

[43] Zhong H Abdullah Deng L Zhao M Tang J Liu T. Probiotic-fermented blueberry juice prevents obesity and hyperglycemia in high fat diet-fed mice in association with modulating the gut microbiota. Food Funct (2020) 11(10):9192–207. doi: 10.1039/d0fo00334d 33030465

[44] Li W Zhang K Yang H. Pectin alleviates high fat (Lard) diet-induced nonalcoholic fatty liver disease in mice: Possible role of short-chain fatty acids and gut microbiota regulated by pectin. J Agric Food Chem (2018) 66(30):8015–25. doi: 10.1021/acs.jafc.8b02979 29987933

[45] Pan Y Guo J Hu N Xun Y Zhang B Feng Q. Distinct common signatures of gut microbiota associated with damp-heat syndrome in patients with different chronic liver diseases. Front Pharmacol (2022) 13:1027628. doi: 10.3389/fphar.2022.1027628 36467028 PMC9712756

[46] Pung HC Lin WS Lo YC Hsu CC Ho CT Pan MH. Ulva prolifera polysaccharide exerts anti-obesity effects *via* upregulation of adiponectin expression and gut microbiota modulation in high-fat diet-fed C57BL/6 mice. J Food Drug Anal (2022) 30(1):46–61. doi: 10.38212/2224-6614.3395 35647728 PMC9931001

[47] Parker BJ Wearsch PA Veloo ACM Rodriguez-Palacios A. The genus alistipes: Gut bacteria with emerging implications to inflammation, cancer, and mental health. Front Immunol (2020) 11:906. doi: 10.3389/fimmu.2020.00906 32582143 PMC7296073

[48] Shkoporov AN Chaplin AV Khokhlova EV Shcherbakova VA Motuzova OV Bozhenko VK. Alistipes inops sp. nov. and coprobacter secundus sp. nov., isolated from human faeces. Int J Syst Evol Microbiol (2015) 65(12):4580–8. doi: 10.1099/ijsem.0.000617 26377180

[49] Feng Q Liang S Jia H Stadlmayr A Tang L Lan Z. Gut microbiome development along the colorectal adenoma-carcinoma sequence. Nat Commun (2015) 6:6528. doi: 10.1038/ncomms7528 25758642

[50] Tsai KY Wu DC Wu WJ Wang JW Juan YS Li CC. Exploring the association between gut and urine microbiota and prostatic disease including benign prostatic hyperplasia and prostate cancer using 16S rRNA sequencing. Biomedicines (2022) 10(11):2676. doi: 10.3390/biomedicines10112676 36359196 PMC9687995

[51] Forbes JD Chen CY Knox NC Marrie RA El-Gabalawy H de Kievit T. A comparative study of the gut microbiota in immune-mediated inflammatory diseases-does a common dysbiosis exist? Microbiome (2018) 6(1):221. doi: 10.1186/s40168-018-0603-4 30545401 PMC6292067

[52] Wright EK Kamm MA Wagner J Teo SM Cruz P Hamilton AL. Microbial factors associated with postoperative crohn's disease recurrence. J Crohns Colitis (2017) 11(2):191–203. doi: 10.1093/ecco-jcc/jjw136 27466174

[53] Osborne G Wu F Yang L Kelly D Hu J Li H. The association between gut microbiome and anthropometric measurements in bangladesh. Gut Microbes (2020) 11(1):63–76. doi: 10.1080/19490976.2019.1614394 31138061 PMC6973329

[54] He Y Wu W Wu S Zheng HM Li P Sheng HF. Linking gut microbiota, metabolic syndrome and economic status based on a population-level analysis. Microbiome (2018) 6(1):172. doi: 10.1186/s40168-018-0557-6 30249275 PMC6154942

[55] Zeng Q Li D He Y Li Y Yang Z Zhao X. Discrepant gut microbiota markers for the classification of obesity-related metabolic abnormalities. Sci Rep (2019) 9(1):13424. doi: 10.1038/s41598-019-49462-w 31530820 PMC6748942

[56] Tito RY Chaffron S Caenepeel C Lima-Mendez G Wang J Vieira-Silva S. Population-level analysis of blastocystis subtype prevalence and variation in the human gut microbiota. Gut (2019) 68(7):1180–9. doi: 10.1136/gutjnl-2018-316106 PMC6582744 30171064

[57] Le Chatelier E Nielsen T Qin J Prifti E Hildebrand F Falony G. Richness of human gut microbiome correlates with metabolic markers. Nature (2013) 500(7464):541–6. doi: 10.1038/nature12506 23985870

[58] Vieira-Silva S Falony G Darzi Y Lima-Mendez G Garcia Yunta R Okuda S. Species-function relationships shape ecological properties of the human gut microbiome. Nat Microbiol (2016) 1(8):16088. doi: 10.1038/nmicrobiol.2016.88 27573110

[59] Konikoff T Gophna U. Oscillospira: a central, enigmatic component of the human gut microbiota. Trends Microbiol (2016) 24(7):523–4. doi: 10.1016/j.tim.2016.02.015 26996766

[60] Zhang J Guo Z Xue Z Sun Z Zhang M Wang L. A phylo-functional core of gut microbiota in healthy young chinese cohorts across lifestyles, geography and ethnicities. ISME J (2015) 9(9):1979–90. doi: 10.1038/ismej.2015.11 PMC4542028 25647347

[61] Valles-Colomer M Falony G Darzi Y Tigchelaar EF Wang J Tito RY. The neuroactive potential of the human gut microbiota in quality of life and depression. Nat Microbiol (2019) 4(4):623–32. doi: 10.1038/s41564-018-0337-x 30718848

[62] Precup G Vodnar DC. Gut prevotella as a possible biomarker of diet and its eubiotic versus dysbiotic roles: a comprehensive literature review. Br J Nutr (2019) 122(2):131–40. doi: 10.1017/S0007114519000680 30924428

[63] Duncan SH Barcenilla A Stewart CS Pryde SE Flint HJ. Acetate utilization and butyryl coenzyme a (CoA):acetate-CoA transferase in butyrate-producing bacteria from the human large intestine. Appl Environ Microbiol (2002) 68(10):5186–90. doi: 10.1128/AEM.68.10.5186-5190.2002 PMC126392 12324374

[64] Hiippala K Jouhten H Ronkainen A Hartikainen A Kainulainen V Jalanka J. The potential of gut commensals in reinforcing intestinal barrier function and alleviating inflammation. Nutrients (2018) 10(8):988. doi: 10.3390/nu10080988 30060606 PMC6116138

[65] Li W Zhu Y Li Y Shu M Wen Y Gao X. The gut microbiota of hand, foot and mouth disease patients demonstrates down-regulated butyrate-producing bacteria and up-regulated inflammation-inducing bacteria. Acta Paediatr (2019) 108(6):1133–9. doi: 10.1111/apa.14644 30427066

[66] Plovier H Everard A Druart C Depommier C Van Hul M Geurts L. A purified membrane protein from akkermansia muciniphila or the pasteurized bacterium improves metabolism in obese and diabetic mice. Nat Med (2017) 23(1):107–13. doi: 10.1038/nm.4236 27892954

[67] Hou Q Zhao F Liu W Lv R Khine WWT Han J. Probiotic-directed modulation of gut microbiota is basal microbiome dependent. Gut Microbes (2020) 12(1):1736974. doi: 10.1080/19490976.2020.1736974 32200683 PMC7524168

[68] Chen YR Jing QL Chen FL Zheng H Chen LD Yang ZC. Desulfovibrio is not always associated with adverse health effects in the guangdong gut microbiome project. PeerJ (2021) 9:e12033. doi: 10.7717/peerj.12033 34466295 PMC8380029

[69] Zhang L Ouyang Y Li H Shen L Ni Y Fang Q. Metabolic phenotypes and the gut microbiota in response to dietary resistant starch type 2 in normal-weight subjects: a randomized crossover trial. Sci Rep (2019) 9(1):4736. doi: 10.1038/s41598-018-38216-9 30894560 PMC6426958

[70] Wan F Wang M Zhong R Chen L Han H Liu L. Supplementation with chinese medicinal plant extracts from lonicera hypoglauca and scutellaria baicalensis mitigates colonic inflammation by regulating oxidative stress and gut microbiota in a colitis mouse model. Front Cell Infect Microbiol (2021) 11:798052. doi: 10.3389/fcimb.2021.798052 35059326 PMC8763710

