# Supplementary material for: Investigating causal associations among gut microbiota, metabolites, and liver diseases: a Mendelian randomization study
Source: Front Endocrinol (Lausanne). 2023 Jul 5;14:1159148. doi: 10.3389/fendo.2023.1159148 (PMC10354516; doi:10.3389/fendo.2023.1159148)
Supplement: Supplementary file 1 [file Table_1.docx]

| Table S1. Association of genetically predicted remaining genera with non-alcoholic fatty liver disease | | | | | | | | | |
| --- | --- | --- | --- | --- | --- | --- | --- | --- | --- |
| Genus | IVW | | |  | MR-Egger | |  | Weighted median | |
|  | IVs | OR(95% CI) | *p* value | | OR(95%CI) | *p* value | | OR(95%CI) | *p* value |
| Actinomyces | 7 | 1.048(0.700-1.569) | 0.821 | | 1.123(0.367-3.436) | 0.847 | | 1.279(0.780-2.098) | 0.330 |
| Adlercreutzia | 8 | 1.075(0.748-1.544) | 0.697 | | 1.175(0.132-10.458) | 0.890 | | 1.082(0.687-1.703) | 0.735 |
| Akkermansia | 10 | 0.854(0.578-1.262) | 0.428 | | 0.420(0.116-1.518) | 0.223 | | 0.842(0.480-1.476) | 0.548 |
| Alistipes | 14 | 0.917(0.733-1.146) | 0.445 | | 0.464(0.063-3.408) | 0.465 | | 0.990(0.764-1.283) | 0.940 |
| Allisonella | 8 | 1.104(0.882-1.381) | 0.389 | | 0.526(0.114-2.427) | 0.442 | | 1.080(0.808-1.445) | 0.602 |
| Alloprevotella | 6 | 0.849(0.632-1.141) | 0.278 | | 8.133(0.376-175.86) | 0.252 | | 0.857(0.568-1.293) | 0.461 |
| Anaerofilum | 11 | 0.995(0.934-1.060) | 0.884 | | 1.479(0.575-3.803) | 0.438 | | 0.993(0.924-1.067) | 0.845 |
| Anaerostipes | 12 | 0.998(0.573-1.739) | 0.996 | | 0.465(0.089-2.417) | 0.384 | | 1.292(0.717-2.328) | 0.393 |
| Bacteroides | 9 | 1.016(0.808-1.278) | 0.890 | | 0.398(0.088-1.788) | 0.268 | | 1.016(0.808-1.278) | 0.945 |
| Bifidobacterium | 13 | 0.908(0.595-1.388) | 0.657 | | 0.538(0.144-2.010) | 0.376 | | 0.954(0.602-1.511) | 0.841 |
| Bilophila | 11 | 0.943(0.751-1.185) | 0.617 | | 0.508(0.084-3.073) | 0.479 | | 0.983(0.741-1.304) | 0.906 |
| Blautia | 12 | 0.781(0.519-1.176) | 0.237 | | 1.276(0.504-3.232) | 0.618 | | 0.841(0.483-1.463) | 0.540 |
| Butyricicoccus | 8 | 0.851(0.564-1.283) | 0.440 | | 0.904(0.458-1.782) | 0.780 | | 0.780(0.441-1.377) | 0.391 |
| Butyricimonas | 13 | 1.006(0.776-1.305) | 0.963 | | 0.745(0.264-2.104) | 0.590 | | 0.910(0.642-1.291) | 0.598 |
| Butyrivibrio | 14 | 0.896(0.749-1.071) | 0.228 | | 1.327(0.476-3.698) | 0.598 | | 0.902(0.707-1.152) | 0.409 |
| Candidatus Soleaferrea | 9 | 1.079(0.858-1.356) | 0.518 | | 1.142(0.048-26.950) | 0.936 | | 1.061(0.813-1.386) | 0.663 |
| Catenibacterium | 5 | 0.887(0.630-1.249) | 0.491 | | 0.795(0.025-25.582) | 0.905 | | 0.977(0.658-1.452) | 0.908 |
| Christensenellaceae R.7 group | 10 | 0.999(0.984-1.014) | 0.848 | | 0.928(0.578-1.489) | 0.764 | | 0.998(0.982-1.015) | 0.839 |
| Clostridium sensu stricto 1 | 7 | 1.490(0.774-2.868) | 0.233 | | 0.593(0.144-2.440) | 0.501 | | 1.059(0.486-2.306) | 0.885 |
| Clostridium innocuum group | 9 | 1.183(0.830-1.688) | 0.352 | | 2.403(0.263-21.984) | 0.463 | | 1.245(0.769-2.015) | 0.372 |
| Collinsella | 9 | 1.001(0.991-1.010) | 0.861 | | 1.050(0.770-1.430) | 0.768 | | 1.001(0.991-1.011) | 0.865 |
| Coprobacter | 11 | 0.980(0.810-1.186) | 0.834 | | 1.063(0.638-1.771) | 0.821 | | 1.062(0.838-1.345) | 0.619 |
| Coprococcus 1 | 10 | 0.997(0.661-1.503) | 0.989 | | 0.805(0.288-2.245) | 0.689 | | 1.015(0.577-1.785) | 0.959 |
| Coprococcus 2 | 8 | 0.899(0.427-1.892) | 0.778 | | 0.047(0001-21.230) | 0.364 | | 1.692(0.832-3.439) | 0.146 |
| Coprococcus 3 | 6 | 0.981(0.771-1.248) | 0.874 | | 0.230(0.002-28.479) | 0.582 | | 0.976(0.736-1.294) | 0.868 |
| Defluviitaleaceae UCG 011 | 9 | 0.817(0.639-1.044) | 0.106 | | 1.396(0.493-3.955) | 0.550 | | 0.838(0.613-1.147) | 0.270 |
| Desulfovibrio | 10 | 1.001(0.734-1.365) | 0.996 | | 0.746(0.227-2.448) | 0.641 | | 1.103(0.743-1.636) | 0.626 |
| Dialister | 11 | 1.002(0.820-1.225) | 0.982 | | 12.351(1.769-86.250) | **0.032** | | 0.997(0.826-1.205) | 0.981 |
| Dorea | 10 | 1.100(0.628-1.928) | 0.738 | | 2.018(0.533-7.598) | 0.333 | | 1.592(0.738-3.433) | 0.236 |
| Eggerthella | 11 | 0.983(0.803-1.205) | 0.872 | | 0.830(0.278-2.479) | 0.746 | | 1.057(0.802-1.392) | 0.695 |
| Eisenbergiella | 11 | 0.886(0.694-1.131) | 0.330 | | 0.633(0.074-5.377) | 0.685 | | 0.823(0.590-1.149) | 0.253 |
| Enterorhabdus | 7 | 1.012(0.844-1.214) | 0.093 | | 0.958(0.575-1.598) | 0.261 | | 0.993(0.806-1.225) | 0.107 |
| Erysipelatoclostridium | 15 | 1.043(0.744-1.462) | 0.809 | | 1.029(0.319-3.317) | 0.963 | | 1.085(0.720-1.636) | 0.696 |
| Erysipelotrichaceae UCG 003 | 16 | 1.161(0.858-1.571) | 0.332 | | 0.737(0.235-2.311) | 0.609 | | 1.014(0.754-1.363) | 0.928 |
| Escherichia Shigella | 10 | 1.099(0.776-1.556) | 0.594 | | 0.880(0.399-1.944) | 0.760 | | 1.034(0.653-1.637) | 0.887 |
| Eubacterium brachy group | 9 | 1.113(0.818-1.514) | 0.495 | | 3.050(0.646-14.388) | 0.202 | | 1.100(0.781-1.550) | 0.586 |
| Eubacterium coprostanoligenes group | 13 | 1.031(0.617-1.724) | 0.907 | | 5.224(0.965-28.289) | 0.081 | | 1.363(0.781-1.376) | 0.276 |
| Eubacterium eligens group | 7 | 0.719(0.501-1.033) | 0.075 | | 0.321(0.069-1.484) | 0.205 | | 0.686(0.437-1.076) | 0.101 |
| Eubacterium fissicatena group | 9 | 1.054(0.847-1.311) | 0.637 | | 1.674(0.417-6.719) | 0.491 | | 0.991(0.807-1.217) | 0.933 |
| Eubacterium hallii group | 16 | 1.002(0.864-1.167) | 0.982 | | 1.065(0.768-1.478) | 0.710 | | 1.023(0.856-1.222) | 0.805 |
| Eubacterium nodatum group | 11 | 1.004(0.978-1.031) | 0.743 | | 1.079(0.591-1.972) | 0.810 | | 1.004(0.978-1.030) | 0.766 |
| Eubacterium rectale group | 8 | 1.161(0.663-2.033) | 0.601 | | 0.232(0.021-2.563) | 0.278 | | 1.118(0.703-1.777) | 0.639 |
| Eubacterium ruminantium group | 18 | 0.988(0.770-1.268) | 0.926 | | 0.509(0.157-1.645) | 0.276 | | 0.909(0.632-1.308) | 0.609 |
| Eubacterium oxidoreducens group | 5 | 1.074(0.717-1.609) | 0.730 | | 0.599(0.163-2.193) | 0.495 | | 1.018(0.611-1.697) | 0.945 |
| Eubacterium ventriosum group | 15 | 0.909(0.767-1.077) | 0.269 | | 0.313(0.093-1.051) | 0.751 | | 0.969(0.800-1.174) | 0.269 |
| Eubacterium xylanophilum group | 9 | 1.037(0.914-1.177) | 0.575 | | 1.098(0.602-2.002) | 0.769 | | 1.028(0.912-1.159) | 0.655 |
| Faecalibacterium | 10 | 0.810(0.480-1.367) | 0.430 | | 2.478(1.025-5.989) | 0.079 | | 1.286(0.805-2.054) | 0.293 |
| Family XIII AD3011 group | 12 | 0.941(0.516-1.717) | 0.842 | | 0.976(0.039-24.645) | 0.988 | | 0.627(0.311-1.264) | 0.192 |
| Family XIII UCG 001 | 8 | 0.855(0.528-1.384) | 0.524 | | 1.023(0.206-5.087) | 0.979 | | 1.046(0.543-2.016) | 0.894 |
| Flavonifractor | 5 | 1.438(0.396-5.221) | 0.581 | | 6.800(0.021-2284.49) | 0.564 | | 0.731(0.308-1.735) | 0.478 |
| Fusicatenibacter | 18 | 0.945(0.771-1.159) | 0.588 | | 1.057(0.396-2.817) | 0.914 | | 0.954(0.732-1.244) | 0.730 |
| Gordonibacter | 12 | 1.052(0.879-1.260) | 0.580 | | 0.732(0.300-1.789) | 0.510 | | 1.026(0.806-1.305) | 0.837 |
| Haemophilus | 9 | 0.767(0.474-1.242) | 0.281 | | 0.251(0.096-0.656) | **0.026** | | 0.778(0.462-1.310) | 0.344 |
| Holdemania | 15 | 0.992(0.936-1.052) | 0.800 | | 1.015(0.753-1.370) | 0.922 | | 0.987(0.925-1.052) | 0.686 |
| Holdemanella | 10 | 0.813(0.588-1.124) | 0.210 | | 0.579(0.236-1.421) | 0.267 | | 0.710(0.451-1.119) | 0.140 |
| Howardella | 10 | 0.839(0.663-1.060) | 0.141 | | 0.6437(0.176-2.357) | 0.525 | | 0.821(0.607-1.112) | 0.203 |
| Hungatella | 5 | 1.258(0.859-1.841) | 0.239 | | 3.233(0.392-26.662) | 0.355 | | 1.327(0.818-2.154) | 0.251 |
| Intestinibacter | 13 | 1.003(0.786-1.280) | 0.979 | | 1.344(0.714-2.528) | 0.379 | | 1.108(0.796-1.543) | 0.542 |
| Lachnospira | 5 | 1.484(0.756-2.911) | 0.251 | | 0.523(0.004-62.700) | 0.808 | | 1.189(0.658-2.148) | 0.566 |
| Lactobacillus | 10 | 1.185(0.876-1.603) | 0.271 | | 1.088(0.435-2.725) | 0.861 | | 1.164(0.753-1.800) | 0.494 |
| Lachnospiraceae FCS020 group | 12 | 0.990(0.819-1.197) | 0.097 | | 1.455(0.513-4.122) | 0.531 | | 0.989(0.796-1.229) | 0.111 |
| Lachnospiraceae ND3007 group | 3 | 1.212(0.390-3.766) | 0.739 | | 152702(0.19-1197697) | 0.335 | | 1.230(0.366-4.133) | 0.738 |
| Lachnospiraceae NK4A136 group | 14 | 0.964(0.720-1.291) | 0.808 | | 0.785(0.467-1.320) | 0.380 | | 0.922(0.617-1.377) | 0.691 |
| Lachnospiraceae UCG 001 | 12 | 0.992(0.800-1.229) | 0.939 | | 0.819(0.230-2.916) | 0.764 | | 1.051(0.784-1.410) | 0.737 |
| Lachnospiraceae UCG 004 | 12 | 1.553(0.896-2.691) | 0.117 | | 1.276(0.110-14.831) | 0.850 | | 1.799(0.927-3.493) | 0.083 |
| Lachnospiraceae UCG 008 | 11 | 0.998(0.958-1.040) | 0.919 | | 0.887(0.135-5.831) | 0.904 | | 0.997(0.957-1.038) | 0.874 |
| Lachnospiraceae UCG 010 | 9 | 1.214(0.891-1.654) | 0.219 | | 1.536(0.571-4.127) | 0.423 | | 1.109(0.778-1.581) | 0.566 |
| Lactococcus | 9 | 1.150(0.905-1.460) | 0.253 | | 1.294(0.384-4.366) | 0.690 | | 1.090(0.776-1.531) | 0.620 |
| Marvinbryantia | 10 | 0.900(0.734-1.103) | 0.309 | | 1.569(0.514-4.790) | 0.452 | | 0.957(0.731-1.254) | 0.753 |
| Methanobrevibacter | 6 | 0.930(0.761-1.136) | 0.475 | | 1.322(0.679-2.575) | 0.458 | | 0.963(0.755-1.227) | 0.758 |
| Odoribacter | 7 | 1.007(0.881-1.151) | 0.916 | | 0.875(0.319-2.399) | 0.806 | | 1.003(0.893-1.127) | 0.958 |
| Oscillibacter | 13 | 0.913(0.715-1.165) | 0.464 | | 0.650(0.256-1.651) | 0.384 | | 0.970(0.687-1.369) | 0.862 |
| Oscillospira | 8 | 0.912(0.701-1.186) | 0.492 | | 0.377(0.083-1.709) | 0.253 | | 0.925(0.706-1.211) | 0.571 |
| Oxalobacter | 11 | 1.016(0.810-1.273) | 0.893 | | 0.716(0.188-2.723) | 0.636 | | 1.036(0.779-1.379) | 0.808 |
| Parabacteroides | 6 | 0.841(0.463-1.530) | 0.571 | | 1.757(0.201-15.329) | 0.637 | | 1.002(0.477-2.108) | 0.995 |
| Paraprevotella | 13 | 0.810(0.564-1.162) | 0.252 | | 0.484(0.136- 1.731) | 0.288 | | 0.795(0.541-1.169) | 0.244 |
| Parasutterella | 14 | 1.012(0.770-1.330) | 0.932 | | 0.867(0.369-2.038) | 0.749 | | 1.077(0.747-1.554) | 0.691 |
| Phascolarctobacterium | 10 | 0.860(0.683-1.085) | 0.203 | | 0.801(0.198-3.241) | 0.763 | | 0.872(0.639-1.188) | 0.384 |
| Prevotella7 | 10 | 0.955(0.770-1.185) | 0.677 | | 0.738(0.191-2.857) | 0.672 | | 0.867(0.643-1.170) | 0.350 |
| Prevotella9 | 10 | 0.950(0.701-1.288) | 0.741 | | 1.227(0.579-2.602) | 0.608 | | 0.957(0.640-1.432) | 0.832 |
| Rikenellaceae RC9 gut group | 11 | 0.985(0.929-1.046) | 0.628 | | 1.396(0.699-2.789) | 0.370 | | 0.978(0.906-1.055) | 0.564 |
| Romboutsia | 13 | 0.971(0.797-1.182) | 0.767 | | 0.848(0.332-2.166) | 0.736 | | 0.977(0.780-1.224) | 0.839 |
| Roseburia | 14 | 1.393(0.834-2.326) | 0.205 | | 0.635(0.145-2.782) | 0.558 | | 1.065(0.570-1.992) | 0.843 |
| Ruminiclostridium 5 | 9 | 1.078(0.671-1.732) | 0.757 | | 2.6874(0.366-19.720) | 0.363 | | 1.108(0.581-2.113) | 0.755 |
| Ruminiclostridium 6 | 16 | 0.896(0.789-1.018) | 0.092 | | 0.820(0.427-1.577) | 0.562 | | 0.939(0.806-1.095) | 0.424 |
| Ruminiclostridium 9 | 9 | 1.009(0.662-1.538) | 0.968 | | 1.825(0.336-9.901) | 0.508 | | 0.789(0.450-1.382) | 0.407 |
| Ruminococcus gauvreauii group | 11 | 0.927(0.709-1.213) | 0.582 | | 0.425(0.023-7.879) | 0.580 | | 0.956(0.731-1.249) | 0.741 |
| Ruminococcus gnavus group | 12 | 1.208(0.817-1.787) | 0.344 | | 11.779(3.238-42.851) | **0.004** | | 1.014(0.703-1.462) | 0.943 |
| Ruminococcus torques group | 7 | 0.984(0.758-1.276) | 0.900 | | 0.813(0.193-3.430) | 0.787 | | 1.053(0.788-1.407) | 0.726 |
| Ruminococcaceae NK4A214 group | 12 | 0.987(0.902-1.080) | 0.778 | | 1.184(0.575-2.436) | 0.657 | | 0.979(0.879-1.089) | 0.694 |
| Ruminococcaceae UCG 002 | 22 | 1.025(0.937-1.121) | 0.586 | | 0.962(0.639-1.449) | 0.854 | | 1.002(0.906-1.108) | 0.970 |
| Ruminococcaceae UCG 003 | 12 | 0.991(0.871-1.128) | 0.893 | | 1.336(0.606-2.945) | 0.488 | | 0.986(0.843-1.152) | 0.858 |
| Ruminococcaceae UCG 004 | 10 | 0.998(0.717-1.391) | 0.993 | | 0.593(0.040-8.741) | 0.713 | | 0.915(0.588-1.424) | 0.695 |
| Ruminococcaceae UCG 005 | 14 | 1.226(0.832-1.805) | 0.303 | | 1.242(0.290-5.321) | 0.776 | | 1.203(0.700-2.067) | 0.503 |
| Ruminococcaceae UCG 009 | 12 | 1.089(0.855-1.387) | 0.490 | | 1.271(0.322-5.011) | 0.739 | | 1.064(0.775-1.461) | 0.702 |
| Ruminococcaceae UCG 010 | 6 | 1.002(0.979-1.026) | 0.845 | | 2.276(0.249-20.770) | 0.506 | | 1.002(0.977-1.027) | 0.882 |
| Ruminococcaceae UCG 011 | 8 | 0.992(0.742-1.326) | 0.956 | | 0.741(0.195-2.824) | 0.676 | | 0.961(0.744-1.241) | 0.762 |
| Ruminococcaceae UCG 013 | 13 | 0.860(0.583-1.268) | 0.447 | | 0.568(0.200-1.607) | 0.311 | | 1.062(0.667-1.691) | 0.801 |
| Ruminococcaceae UCG 014 | 10 | 1.203(0.745-1.943) | 0.450 | | 2.217(0.455-10.802) | 0.353 | | 0.894(0.489-1.636) | 0.717 |
| Ruminococcus 2 | 15 | 1.025(0.806-1.303) | 0.842 | | 2.086(0.642-6.781) | 0.243 | | 1.027(0.820-1.285) | 0.818 |
| Sellimonas | 9 | 1.043(0.901-1.207) | 0.576 | | 1.398(0.456-4.279) | 0.576 | | 1.002(0.825-1.216) | 0.986 |
| Senegalimassilia | 5 | 0.579(0.329-1.022) | 0.059 | | 0.560(0.033-9.408) | 0.714 | | 0.654(0.345-1.241) | 0.194 |
| Slackia | 6 | 0.935(0.819-1.067) | 0.319 | | 0.362(0.058-2.270) | 0.339 | | 0.959(0.824-1.116) | 0.588 |
| Streptococcus | 15 | 1.022(0.651-1.606) | 0.924 | | 1.364(0.257-7.243) | 0.721 | | 1.142(0.660- 1.978) | 0.635 |
| Subdoligranulum | 10 | 1.004(0.844-1.195) | 0.962 | | 0.940(0.344-2.566) | 0.907 | | 1.016(0.827-1.248) | 0.879 |
| Sutterella | 12 | 0.970(0.651-1.446) | 0.881 | | 1.377(0.216-8.764) | 0.742 | | 0.840(0.498-1.416) | 0.513 |
| Terrisporobacter | 4 | 1.004(0.839-1.202) | 0.962 | | 1.250(0.639-2.446) | 0.581 | | 1.013(0.808-1.269) | 0.912 |
| Turicibacter | 10 | 0.989(0.730-1.339) | 0.942 | | 0.375(0.088-1.597) | 0.221 | | 0.967(0.640-1.461) | 0.873 |
| Tyzzerella 3 | 12 | 0.975(0.797-1.192) | 0.801 | | 0.706(0.213-2.344) | 0.582 | | 0.895(0.680-1.178) | 0.428 |
| Veillonella | 7 | 1.187(0.559-2.523) | 0.655 | | 0.003(0.0001-3383.9) | 0.455 | | 1.405(0.627-3.151) | 0.409 |
| Victivallis | 9 | 1.053(0.875-1.267) | 0.587 | | 1.746(0.383-7.969) | 0.495 | | 0.953(0.740-1.227) | 0.709 |
