# Supplementary material for: Investigating causal associations among gut microbiota, metabolites, and liver diseases: a Mendelian randomization study
Source: Front Endocrinol (Lausanne). 2023 Jul 5;14:1159148. doi: 10.3389/fendo.2023.1159148 (PMC10354516; doi:10.3389/fendo.2023.1159148)
Supplement: Supplementary file 2 [file Table_2.docx]

| Table S2. SNPs used as instrumental variables from gut microbiota and their associations with non-alcoholic fatty liver disease | | | | | | | | | | | | |
| --- | --- | --- | --- | --- | --- | --- | --- | --- | --- | --- | --- | --- |
| Genus | SNP | Effect allele | Other allele | | gut microbiota | | | | NAFLD | | | F |
|  |  |  |  |  | Beta | SE | *p* value |  | Beta | SE | *p* value |  |
| Anaerotruncus | rs11018566 | A | G | -0.156 | | 0.037 | 6.14E-06 | | -0.037 | 0.143 | 0.795 | 18.272 |
| Anaerotruncus | rs34449434 | A | C | -0.050 | | 0.011 | 9.85E-06 | | 0.047 | 0.048 | 0.325 | 19.208 |
| Anaerotruncus | rs17734739 | T | C | 0.066 | | 0.015 | 7.43E-06 | | -0.123 | 0.057 | 0.030 | 19.603 |
| Anaerotruncus | rs6494922 | A | G | 0.090 | | 0.020 | 6.62E-06 | | -0.061 | 0.086 | 0.481 | 19.938 |
| Anaerotruncus | rs1431492 | C | T | -0.065 | | 0.015 | 7.36E-06 | | 0.049 | 0.064 | 0.442 | 20.075 |
| Anaerotruncus | rs7155595 | C | A | 0.054 | | 0.012 | 7.55E-06 | | 0.013 | 0.047 | 0.782 | 20.575 |
| Anaerotruncus | rs10150232 | A | G | 0.057 | | 0.012 | 6.68E-06 | | 0.016 | 0.087 | 0.856 | 20.622 |
| Anaerotruncus | rs115414803 | A | C | -0.144 | | 0.032 | 6.83E-06 | | 0.250 | 0.120 | 0.037 | 20.669 |
| Anaerotruncus | rs9347879 | T | C | 0.051 | | 0.011 | 4.22E-06 | | -0.041 | 0.040 | 0.299 | 20.988 |
| Anaerotruncus | rs4669806 | G | T | 0.058 | | 0.012 | 2.42E-06 | | -0.086 | 0.053 | 0.106 | 21.962 |
| Anaerotruncus | rs8005030 | C | T | 0.055 | | 0.012 | 2.28E-06 | | -0.048 | 0.047 | 0.303 | 22.132 |
| Anaerotruncus | rs1272208 | G | T | -0.061 | | 0.013 | 4.28E-06 | | -0.017 | 0.035 | 0.628 | 22.202 |
| Anaerotruncus | rs6563550 | T | C | 0.088 | | 0.018 | 2.35E-07 | | 0.023 | 0.057 | 0.687 | 24.629 |
| Intestinimonas | rs17067892 | C | T | 0.107 | | 0.025 | 6.38E-06 | | 0.075 | 0.072 | 0.299 | 18.383 |
| Intestinimonas | rs62427239 | C | A | 0.163 | | 0.037 | 9.41E-06 | | -0.236 | 0.091 | 0.009 | 19.574 |
| Intestinimonas | rs9823439 | T | C | -0.058 | | 0.013 | 9.86E-06 | | -0.033 | 0.041 | 0.420 | 19.598 |
| Intestinimonas | rs4113676 | A | C | -0.219 | | 0.049 | 7.42E-06 | | -0.050 | 0.156 | 0.751 | 19.873 |
| Intestinimonas | rs2276760 | A | G | -0.069 | | 0.015 | 7.84E-06 | | 0.046 | 0.035 | 0.190 | 20.178 |
| Intestinimonas | rs4784055 | T | C | -0.175 | | 0.039 | 8.72E-07 | | 0.167 | 0.091 | 0.066 | 20.631 |
| Intestinimonas | rs72982915 | C | T | 0.183 | | 0.040 | 4.91E-06 | | -0.016 | 0.062 | 0.797 | 20.682 |
| Intestinimonas | rs1859797 | G | A | 0.060 | | 0.013 | 4.12E-06 | | -0.097 | 0.042 | 0.020 | 20.981 |
| Intestinimonas | rs6934519 | C | T | 0.069 | | 0.015 | 8.57E-06 | | -0.017 | 0.028 | 0.551 | 20.982 |
| Intestinimonas | rs7170984 | T | C | -0.066 | | 0.014 | 2.98E-06 | | 0.049 | 0.035 | 0.164 | 21.858 |
| Intestinimonas | rs2731794 | C | T | 0.121 | | 0.026 | 1.92E-06 | | 0.009 | 0.383 | 0.981 | 21.942 |
| Intestinimonas | rs10262702 | T | C | 0.092 | | 0.019 | 2.06E-06 | | -0.047 | 0.048 | 0.329 | 22.189 |
| Intestinimonas | rs2930225 | G | T | 0.073 | | 0.015 | 1.35E-06 | | 0.031 | 0.057 | 0.582 | 22.750 |
| Intestinimonas | rs62240188 | G | A | 0.130 | | 0.027 | 2.20E-06 | | -0.031 | 0.064 | 0.627 | 23.701 |
| Intestinimonas | rs12226153 | A | G | -0.151 | | 0.031 | 5.12E-07 | | 0.067 | 0.075 | 0.375 | 24.250 |
| Intestinimonas | rs11258178 | A | G | 0.066 | | 0.013 | 6.98E-07 | | 0.013 | 0.030 | 0.664 | 24.264 |
| Intestinimonas | rs716604 | A | G | 0.082 | | 0.017 | 8.57E-07 | | 0.035 | 0.073 | 0.629 | 24.289 |
| Lachnoclostridium | rs2385421 | A | G | 0.075 | | 0.018 | 7.14E-06 | | 0.146 | 0.092 | 0.115 | 17.046 |
| Lachnoclostridium | rs72829893 | G | T | 0.117 | | 0.027 | 5.58E-06 | | -0.027 | 0.097 | 0.784 | 19.199 |
| Lachnoclostridium | rs12566975 | T | C | -0.047 | | 0.011 | 9.57E-06 | | -0.028 | 0.055 | 0.612 | 19.580 |
| Lachnoclostridium | rs1528479 | G | A | -0.050 | | 0.011 | 9.64E-06 | | 0.135 | 0.040 | 0.001 | 19.783 |
| Lachnoclostridium | rs1997204 | T | C | -0.108 | | 0.024 | 5.97E-06 | | 0.128 | 0.089 | 0.153 | 19.941 |
| Lachnoclostridium | rs1031599 | G | T | -0.079 | | 0.018 | 6.31E-06 | | 0.072 | 0.047 | 0.127 | 20.039 |
| Lachnoclostridium | rs3821998 | C | A | -0.086 | | 0.019 | 6.72E-06 | | 0.064 | 0.070 | 0.359 | 20.144 |
| Lachnoclostridium | rs4738679 | G | A | -0.052 | | 0.011 | 4.42E-06 | | 0.038 | 0.040 | 0.337 | 20.813 |
| Lachnoclostridium | rs78068103 | A | G | 0.089 | | 0.019 | 3.67E-06 | | 0.006 | 0.253 | 0.981 | 20.814 |
| Lachnoclostridium | rs789029 | C | T | -0.064 | | 0.014 | 3.75E-06 | | 0.030 | 0.089 | 0.739 | 21.603 |
| Lachnoclostridium | rs62285313 | A | G | 0.086 | | 0.018 | 1.58E-06 | | 0.086 | 0.061 | 0.161 | 22.655 |
| Lachnoclostridium | rs615997 | T | C | 0.051 | | 0.011 | 2.03E-06 | | -0.045 | 0.049 | 0.357 | 23.094 |
| Lachnoclostridium | rs6112314 | A | C | -0.056 | | 0.011 | 2.43E-07 | | 0.060 | 0.030 | 0.047 | 26.964 |
| Lachnospiraceae NC2004 group | rs117467633 | T | C | -0.170 | | 0.038 | 9.13E-06 | | 0.057 | 0.119 | 0.630 | 19.612 |
| Lachnospiraceae NC2005 group | rs12127733 | G | A | 0.115 | | 0.025 | 3.11E-06 | | -0.067 | 0.055 | 0.226 | 21.922 |
| Lachnospiraceae NC2006 group | rs12208226 | C | A | -0.155 | | 0.034 | 9.75E-06 | | 0.145 | 0.065 | 0.026 | 20.668 |
| Lachnospiraceae NC2007 group | rs12863463 | G | A | -0.156 | | 0.035 | 6.04E-06 | | -0.016 | 0.046 | 0.733 | 20.498 |
| Lachnospiraceae NC2009 group | rs17067076 | G | A | -0.155 | | 0.035 | 5.61E-06 | | 0.016 | 0.221 | 0.943 | 19.277 |
| Lachnospiraceae NC2010 group | rs1928659 | T | C | 0.103 | | 0.023 | 6.17E-06 | | -0.091 | 0.040 | 0.022 | 20.498 |
| Lachnospiraceae NC2011 group | rs1929743 | T | C | 0.084 | | 0.019 | 9.06E-06 | | -0.006 | 0.065 | 0.932 | 19.351 |
| Lachnospiraceae NC2012 group | rs3756315 | A | G | -0.088 | | 0.019 | 3.33E-06 | | 0.046 | 0.033 | 0.161 | 21.991 |
| Lachnospiraceae NC2013 group | rs6116753 | G | A | 0.099 | | 0.021 | 2.92E-06 | | -0.010 | 0.042 | 0.813 | 22.623 |
| Olsenella | rs8066522 | G | A | -0.107 | | 0.024 | 9.70E-06 | | 0.041 | 0.038 | 0.277 | 19.640 |
| Olsenella | rs9460691 | C | A | 0.120 | | 0.027 | 7.28E-06 | | -0.013 | 0.028 | 0.639 | 19.941 |
| Olsenella | rs61090148 | A | G | -0.105 | | 0.023 | 6.44E-06 | | -0.016 | 0.053 | 0.763 | 20.515 |
| Olsenella | rs6046522 | C | T | 0.123 | | 0.027 | 4.48E-06 | | -0.061 | 0.045 | 0.176 | 20.743 |
| Olsenella | rs1035588 | A | G | -0.108 | | 0.024 | 4.86E-06 | | 0.030 | 0.034 | 0.386 | 20.850 |
| Olsenella | rs7540303 | C | T | 0.108 | | 0.024 | 5.32E-06 | | -0.047 | 0.043 | 0.273 | 20.892 |
| Olsenella | rs35225860 | A | G | -0.224 | | 0.048 | 3.87E-06 | | 0.015 | 0.106 | 0.888 | 21.486 |
| Olsenella | rs2759329 | G | A | -0.111 | | 0.024 | 3.43E-06 | | 0.038 | 0.032 | 0.234 | 21.947 |
| Olsenella | rs17148768 | G | A | 0.140 | | 0.030 | 2.20E-06 | | -0.090 | 0.048 | 0.058 | 22.570 |
| Olsenella | rs72691585 | C | A | -0.249 | | 0.052 | 2.95E-06 | | -0.072 | 0.108 | 0.500 | 22.872 |
| Olsenella | rs62112538 | C | T | -0.199 | | 0.041 | 1.19E-06 | | -0.017 | 0.950 | 0.986 | 24.006 |
| Peptococcus | rs12069354 | C | T | 0.168 | | 0.038 | 9.28E-06 | | -0.171 | 0.102 | 0.092 | 19.511 |
| Peptococcus | rs72850165 | T | C | -0.134 | | 0.030 | 5.74E-06 | | 0.064 | 0.033 | 0.055 | 19.985 |
| Peptococcus | rs5770862 | T | C | 0.162 | | 0.036 | 3.22E-06 | | -0.097 | 0.086 | 0.257 | 20.618 |
| Peptococcus | rs74592222 | G | A | 0.138 | | 0.030 | 8.55E-06 | | -0.028 | 0.193 | 0.885 | 20.735 |
| Peptococcus | rs36121075 | A | G | -0.141 | | 0.031 | 6.99E-06 | | -0.028 | 0.136 | 0.835 | 21.094 |
| Peptococcus | rs413827 | G | A | 0.110 | | 0.024 | 3.30E-06 | | 0.001 | 0.036 | 0.978 | 21.537 |
| Peptococcus | rs6918730 | G | A | 0.135 | | 0.029 | 1.15E-06 | | 0.011 | 0.038 | 0.773 | 21.809 |
| Peptococcus | rs7033353 | T | G | 0.090 | | 0.019 | 2.22E-06 | | 0.005 | 0.022 | 0.824 | 22.525 |
| Peptococcus | rs2054133 | G | A | 0.090 | | 0.019 | 2.14E-06 | | -0.115 | 0.041 | 0.005 | 22.606 |
| Peptococcus | rs11001941 | G | A | -0.196 | | 0.039 | 1.33E-06 | | 0.009 | 0.050 | 0.859 | 24.873 |
| Peptococcus | rs77681628 | C | T | 0.200 | | 0.039 | 2.69E-07 | | -0.119 | 0.067 | 0.076 | 26.745 |
| Peptococcus | rs10031059 | T | C | -0.121 | | 0.023 | 1.24E-07 | | 0.009 | 0.021 | 0.669 | 28.784 |
| Ruminococcus 1 | rs3819978 | C | T | -0.115 | | 0.026 | 8.74E-06 | | -0.088 | 0.102 | 0.388 | 19.557 |
| Ruminococcus 1 | rs10167839 | A | G | 0.052 | | 0.012 | 8.09E-06 | | -0.022 | 0.058 | 0.710 | 19.952 |
| Ruminococcus 1 | rs78572139 | G | A | 0.125 | | 0.028 | 5.23E-06 | | 0.075 | 0.092 | 0.412 | 20.026 |
| Ruminococcus 1 | rs6105066 | T | C | -0.061 | | 0.013 | 5.06E-06 | | -0.090 | 0.044 | 0.042 | 20.447 |
| Ruminococcus 1 | rs11783695 | G | T | -0.073 | | 0.016 | 4.73E-06 | | -0.004 | 0.049 | 0.935 | 20.689 |
| Ruminococcus 1 | rs78613526 | G | A | 0.167 | | 0.037 | 5.11E-06 | | 0.250 | 0.102 | 0.014 | 20.757 |
| Ruminococcus 1 | rs6493760 | C | T | 0.054 | | 0.012 | 3.38E-06 | | 0.045 | 0.037 | 0.228 | 21.334 |
| Ruminococcus 1 | rs7583465 | C | T | 0.053 | | 0.011 | 2.56E-06 | | 0.029 | 0.058 | 0.620 | 21.952 |
| Ruminococcus 1 | rs17781867 | C | T | 0.100 | | 0.021 | 1.96E-06 | | -0.038 | 0.067 | 0.569 | 22.275 |
| Ruminococcus 1 | rs7117576 | A | G | 0.083 | | 0.017 | 6.48E-07 | | 0.024 | 0.072 | 0.743 | 23.561 |
