# Supplementary material for: Investigating causal associations among gut microbiota, metabolites, and liver diseases: a Mendelian randomization study
Source: Front Endocrinol (Lausanne). 2023 Jul 5;14:1159148. doi: 10.3389/fendo.2023.1159148 (PMC10354516; doi:10.3389/fendo.2023.1159148)
Supplement: Supplementary file 3 [file Table_3.docx]

| Table S3. Association of genetically predicted remaining genera with alcoholic liver disease | | | | | | | | | |
| --- | --- | --- | --- | --- | --- | --- | --- | --- | --- |
| Genus | IVW | | |  | MR-Egger | |  | Weighted median | |
|  | IVs | OR(95% CI) | *p* value | | OR(95%CI) | *p* value | | OR(95%CI) | *p* value |
| Actinomyces | 7 | 1.095(0.845-1.420) | 0.492 | | 0.900(0.478-1.696) | 0.759 | | 1.072(0.773-1.488) | 0.676 |
| Adlercreutzia | 8 | 1.124(0.851-1.484) | 0.409 | | 2.533(0.731-8.776) | 0.193 | | 1.252(0.877-1.785) | 0.215 |
| Akkermansia | 11 | 0.857(0.607-1.208) | 0.378 | | 0.632(0.190-2.105) | 0.474 | | 0.797(0.551-1.151) | 0.226 |
| Alistipes | 12 | 0.882(0.595-1.309) | 0.533 | | 2.178(0.331-14.323) | 0.437 | | 1.060(0.636-1.767) | 0.823 |
| Allisonella | 8 | 1.017(0.824-1.254) | 0.876 | | 0.938(0.202-4.345) | 0.937 | | 1.085(0.870-1.352) | 0.468 |
| Alloprevotella | 5 | 1.123(0.912-1.384) | 0.276 | | 0.826(0.135-5.055) | 0.849 | | 1.036(0.788-1.363) | 0.799 |
| Anaerotruncus | 13 | 0.899(0.667-1.210) | 0.482 | | 0.639(0.269-1.517) | 0.332 | | 0.969(0.648-1.449) | 0.878 |
| Anaerofilum | 10 | 1.110(0.918-1.341) | 0.281 | | 1.821(0.650-5.101) | 0.287 | | 0.995(0.782-1.267) | 0.970 |
| Anaerostipes | 13 | 1.023(0.750-1.397) | 0.884 | | 0.945(0.305-2.925) | 0.923 | | 1.005(0.656-1.540) | 0.981 |
| Bacteroides | 8 | 0.831(0.552-1.253) | 0.377 | | 0.994(0.096-10.344) | 0.996 | | 0.790(0.456-1.367) | 0.399 |
| Barnesiella | 12 | 1.113(0.832-1.488) | 0.471 | | 0.782(0.236-2.585) | 0.695 | | 1.118(0.751-1.664) | 0.583 |
| Bifidobacterium | 12 | 1.291(0.983-1.652) | 0.072 | | 2.263(0.826-4.176) | 0.066 | | 1.440(0.915-2.043) | 0.071 |
| Bilophila | 13 | 0.780(0.587-1.037) | 0.088 | | 1.057(0.246-4.545) | 0.942 | | 0.909(0.632-1.306) | 0.605 |
| Blautia | 12 | 0.947(0.683-1.314) | 0.745 | | 0.818(0.350-1.909) | 0.652 | | 1.086(0.696-1.696) | 0.716 |
| Butyricicoccus | 8 | 0.833(0.592-1.172) | 0.295 | | 0.907(0.463-1.774) | 0.785 | | 0.887(0.570-1.380) | 0.594 |
| Butyricimonas | 13 | 0.904(0.699-1.169) | 0.441 | | 0.584(0.234-1.457) | 0.274 | | 1.028(0.733-1.442) | 0.873 |
| Butyrivibrio | 15 | 0.987(0.871-1.118) | 0.836 | | 1.029(0.601-1.760) | 0.920 | | 1.041(0.873-1.240) | 0.656 |
| Candidatus Soleaferrea | 9 | 1.091(0.877-1.358) | 0.436 | | 0.077(0.008-0.718) | 0.059 | | 1.136(0.842-1.533) | 0.403 |
| Catenibacterium | 4 | 1.188(0.928-1.521) | 0.172 | | 2.182(0.099-48.243) | 0.670 | | 1.206(0.916-1.588) | 0.183 |
| Christensenellaceae R.7 group | 8 | 1.045(0.700-1.560) | 0.830 | | 0.366(0.089-1.500) | 0.212 | | 1.095(0.643-1.865) | 0.739 |
| Clostridium sensu stricto 1 | 6 | 0.950(0.676-1.334) | 0.766 | | 1.004(0.359-2.807) | 0.995 | | 0.868(0.565-1.334) | 0.519 |
| Clostridium innocuum group | 8 | 0.982(0.817-1.180) | 0.843 | | 1.879(0.747-4.730) | 0.229 | | 1.032(0.805-1.323) | 0.803 |
| Collinsella | 9 | 0.865(0.602-1.242) | 0.432 | | 0.867(0.214-3.505) | 0.847 | | 0.905(0.546-1.501) | 0.700 |
| Coprobacter | 11 | 1.226(0.979-1.536) | 0.076 | | 1.733(0.707-4.248) | 0.260 | | 1.084(0.816-1.440) | 0.578 |
| Coprococcus 1 | 12 | 0.784(0.546-1.126) | 0.188 | | 0.638(0.246-1.653) | 0.376 | | 0.687(0.440-1.075) | 0.100 |
| Coprococcus 2 | 8 | 1.221(0.813-1.833) | 0.336 | | 0.049(0.004-0.634) | 0.061 | | 1.215(0.785-1.879) | 0.382 |
| Coprococcus 3 | 8 | 0.863(0.583-1.276) | 0.460 | | 0.337(0.038-3.020) | 0.368 | | 0.910(0.571-1.451) | 0.691 |
| Defluviitaleaceae UCG 011 | 9 | 1.197(0.905-1.582) | 0.207 | | 1.622(0.567-4.639) | 0.397 | | 1.090(0.749-1.588) | 0.653 |
| Dialister | 11 | 0.859(0.639-1.154) | 0.312 | | 1.425(0.415-4.890) | 0.587 | | 0.958(0.636-1.443) | 0.837 |
| Dorea | 10 | 0.987(0.691-1.411) | 0.945 | | 1.438(0.532-3.884) | 0.494 | | 1.069(0.667-1.712) | 0.782 |
| Eggerthella | 9 | 1.040(0.822-1.314) | 0.746 | | 1.305(0.427-3.986) | 0.654 | | 1.039(0.775-1.391) | 0.800 |
| Eisenbergiella | 11 | 0.972(0.782-1.209) | 0.801 | | 1.651(0.312-8.727) | 0.569 | | 0.920(0.706-1.200) | 0.540 |
| Enterorhabdus | 6 | 1.248(0.920-1.695) | 0.155 | | 1.200(0.487-2.954) | 0.713 | | 1.276(0.841-1.938) | 0.252 |
| Erysipelatoclostridium | 15 | 0.829(0.671-1.024) | 0.082 | | 0.998(0.436-2.288) | 0.997 | | 0.980(0.727-1.321) | 0.896 |
| Erysipelotrichaceae UCG 003 | 16 | 0.827(0.633-1.082) | 0.165 | | 1.312(0.643-2.677) | 0.467 | | 1.019(0.724-1.433) | 0.916 |
| Escherichia Shigella | 10 | 1.059(0.735-1.525) | 0.759 | | 1.032(0.310-3.442) | 0.960 | | 1.087(0.712-1.658) | 0.700 |
| Eubacterium brachy group | 9 | 1.018(0.857-1.209) | 0.841 | | 1.118(0.558-2.240) | 0.761 | | 1.047(0.848-1.291) | 0.671 |
| Eubacterium coprostanoligenes group | 12 | 0.972(0.701-1.347) | 0.864 | | 0.599(0.172-2.085) | 0.439 | | 1.004(0.648-1.555) | 0.987 |
| Eubacterium eligens group | 6 | 1.182(0.784-1.782) | 0.423 | | 1.007(0.217-4.680) | 0.993 | | 1.048(0.617-1.780) | 0.862 |
| Eubacterium fissicatena group | 9 | 1.080(0.899-1.298) | 0.411 | | 1.554(0.598-4.036) | 0.396 | | 1.071(0.840-1.364) | 0.581 |
| Eubacterium hallii group | 14 | 1.068(0.797-1.431) | 0.659 | | 1.708(0.979-2.981) | 0.084 | | 1.029(0.700-1.513) | 0.884 |
| Eubacterium nodatum group | 11 | 1.109(0.954-1.288) | 0.177 | | 0.722(0.370-1.405) | 0.362 | | 1.061(0.868-1.297) | 0.563 |
| Eubacterium rectale group | 8 | 1.181(0.808-1.725) | 0.390 | | 0.557(0.139-2.228) | 0.440 | | 1.268(0.775-2.076) | 0.344 |
| Eubacterium ruminantium group | 18 | 1.150(0.974-1.358) | 0.099 | | 1.054(0.605-1.835) | 0.856 | | 1.138(0.902-1.434) | 0.276 |
| Eubacterium oxidoreducens group | 5 | 0.885(0.673-1.165) | 0.384 | | 0.624(0.226-1.727) | 0.431 | | 0.888(0.622-1.270) | 0.516 |
| Eubacterium ventriosum group | 15 | 0.862(0.661-1.125) | 0.275 | | 1.599(0.487-5.248) | 0.453 | | 0.997(0.701-1.418) | 0.987 |
| Eubacterium xylanophilum group | 9 | 0.890(0.664-1.194) | 0.438 | | 1.457(0.605-3.507) | 0.429 | | 0.866(0.591-1.269) | 0.460 |
| Faecalibacterium | 10 | 0.770(0.557-1.065) | 0.114 | | 0.661(0.342-1.278) | 0.253 | | 0.823(0.553-1.224) | 0.337 |
| Family XIII AD3011 group | 13 | 1.080(0.766-1.522) | 0.660 | | 5.934(1.534-22.946) | **0.026** | | 1.094(0.720-1.661) | 0.675 |
| Family XIII UCG 001 | 8 | 1.061(0.766-1.470) | 0.720 | | 0.940(0.344-2.570) | 0.908 | | 0.998(0.655-1.521) | 0.994 |
| Flavonifractor | 5 | 0.880(0.601-1.288) | 0.511 | | 0.792(0.155-4.049) | 0.797 | | 0.870(0.523-1.446) | 0.591 |
| Fusicatenibacter | 18 | 1.103(0.847-1.437) | 0.466 | | 0.809(0.300-2.184) | 0.682 | | 1.098(0.767-1.574) | 0.609 |
| Gordonibacter | 11 | 0.925(0.732-1.168) | 0.510 | | 1.698(0.650-4.435) | 0.308 | | 0.908(0.721-1.145) | 0.415 |
| Haemophilus | 9 | 0.989(0.785-1.247) | 0.927 | | 0.796(0.475-1.334) | 0.923 | | 0.985(0.726-1.337) | 0.415 |
| Holdemania | 14 | 1.064(0.859-1.317) | 0.572 | | 1.444(0.768-2.714) | 0.277 | | 1.000(0.752-1.331) | 0.999 |
| Holdemanella | 11 | 0.986(0.808-1.204) | 0.893 | | 1.056(0.599-1.864) | 0.854 | | 0.942(0.737-1.202) | 0.630 |
| Howardella | 9 | 1.025(0.872-1.205) | 0.765 | | 1.159(0.610-2.202) | 0.667 | | 1.030(0.833-1.273) | 0.788 |
| Hungatella | 5 | 1.089(0.851-1.394) | 0.499 | | 1.325(0.297-5.918) | 0.737 | | 1.098(0.823-1.465) | 0.526 |
| Intestinimonas | 16 | 1.011(0.808-1.266) | 0.923 | | 0.831(0.445-1.554) | 0.572 | | 1.190(0.869-1.629) | 0.278 |
| Intestinibacter | 14 | 1.075(0.842-1.374) | 0.561 | | 1.539(0.719-3.296) | 0.289 | | 1.098(0.782-1.543) | 0.588 |
| Lachnoclostridium | 13 | 0.903(0.639-1.277) | 0.564 | | 0.243(0.087-0.680) | 0.021 | | 0.818(0.537-1.246) | 0.350 |
| Lactobacillus | 8 | 1.026(0.742-1.420) | 0.875 | | 1.326(0.554-3.173) | 0.549 | | 1.182(0.870-1.606) | 0.284 |
| Lachnospiraceae FCS020 group | 12 | 0.863(0.660-1.129) | 0.282 | | 0.542(0.265-1.107) | 0.123 | | 0.849(0.595-1.211) | 0.366 |
| Lachnospiraceae NC2004 group | 9 | 1.007(0.806-1.257) | 0.952 | | 1.032(0.403-2.642) | 0.606 | | 0.924(0.684-1.249) | 0.950 |
| Lachnospiraceae ND3007 group | 3 | 0.872(0.465-1.633) | 0.668 | | 0.081(0.001-3228.68) | 0.723 | | 0.880(0.429-1.806) | 0.728 |
| Lachnospiraceae NK4A136 group | 15 | 1.201(0.946-1.525) | 0.132 | | 1.284(0.794-2.078) | 0.327 | | 1.312(0.935-1.841) | 0.116 |
| Lachnospiraceae UCG 001 | 13 | 0.948(0.743-1.210) | 0.670 | | 0.697(0.248-1.959) | 0.508 | | 0.925(0.676-1.266) | 0.626 |
| Lachnospiraceae UCG 004 | 12 | 0.869(0.612-1.234) | 0.431 | | 0.380(0.091-1.589) | 0.214 | | 0.628(0.406-0.971) | **0.036** |
| Lachnospiraceae UCG 008 | 10 | 1.203(0.974-1.486) | 0.086 | | 1.451(0.498-4.229) | 0.514 | | 1.234(0.934-1.629) | 0.139 |
| Lachnospiraceae UCG 010 | 10 | 0.849(0.558-1.291) | 0.444 | | 0.659(0.171-2.535) | 0.561 | | 1.009(0.648-1.572) | 0.968 |
| Lactococcus | 8 | 1.031(0.849-1.253) | 0.755 | | 0.779(0.315-1.922) | 0.607 | | 1.142(0.890-1.466) | 0.297 |
| Marvinbryantia | 10 | 1.331(0.986-1.795) | 0.062 | | 1.677(0.518-5.427) | 0.413 | | 1.330(0.893-1.981) | 0.160 |
| Methanobrevibacter | 6 | 0.875(0.700-1.095) | 0.243 | | 0.868(0.376-2.006) | 0.757 | | 0.873(0.657-1.159) | 0.347 |
| Odoribacter | 7 | 1.038(0.715-1.506) | 0.845 | | 0.629(0.196-2.020) | 0.471 | | 0.950(0.584-1.545) | 0.837 |
| Olsenella | 10 | 1.007(0.860-1.178) | 0.933 | | 1.301(0.785-2.156) | 0.337 | | 1.011(0.819-1.248) | 0.917 |
| Oscillibacter | 13 | 1.065(0.827-1.370) | 0.627 | | 0.763(0.285-2.047) | 0.602 | | 0.978(0.720-1.329) | 0.888 |
| Oscillospira | 8 | 1.089(0.773-1.534) | 0.626 | | 0.531(0.125-2.255) | 0.424 | | 1.145(0.772-1.697) | 0.501 |
| Oxalobacter | 11 | 1.009(0.811-1.254) | 0.938 | | 0.549(0.202-1.492) | 0.270 | | 1.086(0.847-1.392) | 0.517 |
| Parabacteroides | 5 | 0.635(0.400-1.009) | 0.055 | | 0.926(0.052-16.477) | 0.962 | | 0.599(0.335-1.071) | 0.084 |
| Paraprevotella | 13 | 0.928(0.773-1.115) | 0.425 | | 0.684(0.342-1.367) | 0.305 | | 0.945(0.740-1.208) | 0.653 |
| Parasutterella | 14 | 0.911(0.732-1.133) | 0.401 | | 0.919(0.502-1.683) | 0.790 | | 1.000(0.736-1.359) | 1.000 |
| Peptococcus | 12 | 0.968(0.783-1.197) | 0.765 | | 0.948(0.403-2.228) | 0.904 | | 0.957(0.745-1.229) | 0.729 |
| Phascolarctobacterium | 8 | 0.866(0.583-1.287) | 0.476 | | 1.974(0.303-12.836) | 0.503 | | 0.845(0.547-1.307) | 0.449 |
| Prevotella7 | 11 | 0.942(0.795-1.116) | 0.491 | | 1.083(0.385-3.045) | 0.884 | | 0.901(0.722-1.124) | 0.356 |
| Prevotella9 | 15 | 0.946(0.774-1.156) | 0.588 | | 1.230(0.686-2.205) | 0.500 | | 0.946(0.725-1.234) | 0.681 |
| Rikenellaceae RC9 gut group | 11 | 1.057(0.888-1.258) | 0.532 | | 1.095(0.349-3.431) | 0.880 | | 0.965(0.781-1.192) | 0.741 |
| Romboutsia | 13 | 0.816(0.604-1.101) | 0.184 | | 0.644(0.266-1.556) | 0.349 | | 0.907(0.620-1.326) | 0.613 |
| Roseburia | 14 | 1.340(0.953-1.885) | 0.092 | | 1.274(0.426-3.806) | 0.672 | | 1.200(0.779-1.847) | 0.408 |
| Ruminiclostridium 5 | 10 | 1.080(0.694-1.680) | 0.735 | | 3.961(0.735-21.353) | 0.148 | | 1.048(0.638-1.722) | 0.852 |
| Ruminiclostridium 6 | 15 | 1.052(0.814-1.359) | 0.701 | | 0.731(0.386-1.383) | 0.353 | | 1.148(0.806-1.634) | 0.445 |
| Ruminiclostridium 9 | 8 | 0.658(0.411-1.056) | 0.083 | | 0.181(0.020-1.664) | 0.182 | | 0.802(0.470-1.368) | 0.418 |
| Ruminococcus gauvreauii group | 11 | 1.048(0.789-1.393) | 0.743 | | 0.875(0.273-2.804) | 0.827 | | 1.024(0.709-1.479) | 0.899 |
| Ruminococcus gnavus group | 11 | 1.062(0.869-1.298) | 0.557 | | 0.802(0.301-2.142) | 0.671 | | 0.996(0.746-1.330) | 0.979 |
| Ruminococcaceae NK4A214 group | 13 | 1.134(0.856-1.502) | 0.380 | | 1.109(0.439-2.802) | 0.831 | | 1.093(0.756-1.578) | 0.637 |
| Ruminococcaceae UCG 003 | 12 | 1.270(0.968-1.668) | 0.085 | | 2.135(0.875-5.209) | 0.127 | | 1.297(0.887-1.900) | 0.100 |
| Ruminococcaceae UCG 004 | 11 | 0.944(0.738-1.208) | 0.647 | | 0.612(0.156-2.395) | 0.499 | | 0.907(0.670-1.226) | 0.525 |
| Ruminococcaceae UCG 005 | 14 | 1.001(0.777-1.289) | 0.993 | | 0.757(0.380-1.505) | 0.442 | | 1.044(0.732-1.491) | 0.811 |
| Ruminococcaceae UCG 009 | 11 | 0.862(0.653-1.138) | 0.294 | | 0.455(0.161-1.287) | 0.172 | | 0.840(0.614-1.149) | 0.276 |
| Ruminococcaceae UCG 010 | 6 | 0.731(0.512-1.044) | 0.085 | | 0.937(0.354-2.483) | 0.902 | | 0.730(0.475-1.122) | 0.151 |
| Ruminococcaceae UCG 011 | 8 | 1.070(0.890-1.285) | 0.473 | | 1.057(0.393-2.847) | 0.916 | | 1.147(0.902-1.459) | 0.263 |
| Ruminococcaceae UCG 013 | 11 | 0.880(0.648-1.197) | 0.417 | | 1.257(0.518-3.051) | 0.626 | | 0.985(0.633-1.534) | 0.948 |
| Ruminococcaceae UCG 014 | 10 | 0.937(0.708-1.239) | 0.646 | | 1.024(0.537-1.952) | 0.945 | | 0.897(0.623-1.293) | 0.561 |
| Ruminococcus 1 | 10 | 1.227(0.857-1.756) | 0.263 | | 1.967(0.762-5.076) | 0.199 | | 0.987(0.648-1.503) | 0.952 |
| Ruminococcus 2 | 15 | 1.118(0.884-1.414) | 0.352 | | 1.342(0.763-2.360) | 0.327 | | 1.190(0.852-1.661) | 0.307 |
| Sellimonas | 9 | 1.027(0.865-1.219) | 0.760 | | 1.017(0.347-2.978) | 0.976 | | 1.033(0.837-1.276) | 0.762 |
| Senegalimassilia | 5 | 0.761(0.465-1.245) | 0.277 | | 2.844(0.617-13.097) | 0.272 | | 0.869(0.523-1.446) | 0.590 |
| Slackia | 6 | 1.077(0.720-1.611) | 0.719 | | 0.847(0.046-15.683) | 0.916 | | 1.017(0.684-1.512) | 0.934 |
| Streptococcus | 12 | 1.046(0.679-1.612) | 0.838 | | 3.517(0.795-15.556) | 0.128 | | 1.343(0.816-2.209) | 0.246 |
| Subdoligranulum | 11 | 0.909(0.659-1.253) | 0.560 | | 0.923(0.386-2.208) | 0.861 | | 0.867(0.579-1.298) | 0.488 |
| Sutterella | 12 | 0.931(0.706-1.229) | 0.615 | | 0.980(0.296-3.246) | 0.974 | | 1.032(0.719-1.480) | 0.866 |
| Terrisporobacter | 5 | 1.175(0.853-1.618) | 0.325 | | 2.427(0.921-6.394) | 0.171 | | 1.090(0.712-1.669) | 0.692 |
| Turicibacter | 9 | 0.801(0.617-1.040) | 0.096 | | 0.668(0.232-1.922) | 0.478 | | 0.733(0.529-1.017) | 0.063 |
| Tyzzerella 3 | 12 | 1.087(0.904-1.307) | 0.374 | | 1.303(0.448-3.792) | 0.638 | | 1.129(0.877-1.452) | 0.346 |
| Veillonella | 5 | 1.087(0.706-1.676) | 0.704 | | 3.841(0.001-36990) | 0.833 | | 1.084(6.444-1.824) | 0.761 |
| Victivallis | 10 | 1.109(0.945-1.302) | 0.206 | | 0.937(0.275-3.189) | 0.919 | | 1.111(0.912-1.354) | 0.296 |
