# Supplementary material for: Investigating causal associations among gut microbiota, metabolites, and liver diseases: a Mendelian randomization study
Source: Front Endocrinol (Lausanne). 2023 Jul 5;14:1159148. doi: 10.3389/fendo.2023.1159148 (PMC10354516; doi:10.3389/fendo.2023.1159148)
Supplement: Supplementary file 4 [file Table_4.docx]

| Table S4. SNPs used as instrumental variables from gut microbiota and their associations with alcoholic liver disease | | | | | | | | | | | | |
| --- | --- | --- | --- | --- | --- | --- | --- | --- | --- | --- | --- | --- |
| Genus | SNP | Effect allele | Other allele | | gut microbiota | | | | ALD | | | F |
|  |  |  |  |  | Beta | SE | *p* value |  | Beta | SE | *p* value |  |
| Desulfovibrio | rs2032031 | A | G | -0.065 | | 0.015 | 9.14E-06 | | 0.006 | 0.028 | 0.826 | 19.40 |
| Desulfovibrio | rs72647089 | T | G | -0.107 | | 0.024 | 8.30E-06 | | 0.015 | 0.053 | 0.779 | 19.84 |
| Desulfovibrio | rs7729080 | C | A | -0.070 | | 0.016 | 9.96E-06 | | 0.003 | 0.031 | 0.935 | 19.87 |
| Desulfovibrio | rs12031543 | T | C | -0.127 | | 0.028 | 6.55E-06 | | -0.004 | 0.041 | 0.915 | 20.36 |
| Desulfovibrio | rs4797774 | G | A | 0.213 | | 0.047 | 5.64E-06 | | -0.090 | 0.072 | 0.211 | 20.45 |
| Desulfovibrio | rs6580353 | T | C | 0.077 | | 0.017 | 4.94E-06 | | -0.019 | 0.036 | 0.595 | 20.63 |
| Desulfovibrio | rs2590913 | G | A | 0.154 | | 0.034 | 6.65E-06 | | -0.125 | 0.065 | 0.055 | 20.72 |
| Desulfovibrio | rs2853179 | C | T | 0.081 | | 0.017 | 2.42E-06 | | -0.051 | 0.034 | 0.138 | 21.72 |
| Desulfovibrio | rs13066142 | G | A | 0.119 | | 0.025 | 3.79E-06 | | -0.036 | 0.049 | 0.467 | 22.55 |
| Desulfovibrio | rs16863365 | A | G | 0.109 | | 0.023 | 1.79E-06 | | -0.055 | 0.068 | 0.422 | 23.23 |
| Lachnospira | rs2520509 | A | G | 0.052 | | 0.012 | 7.42E-06 | | 0.013 | 0.031 | 0.681 | 20.10 |
| Lachnospira | rs159484 | G | A | 0.079 | | 0.018 | 6.68E-06 | | -0.077 | 0.055 | 0.160 | 20.16 |
| Lachnospira | rs4923324 | G | A | -0.062 | | 0.013 | 2.44E-06 | | 0.006 | 0.039 | 0.871 | 21.42 |
| Lachnospira | rs4686798 | T | C | 0.053 | | 0.011 | 2.74E-06 | | 0.000 | 0.030 | 0.995 | 21.86 |
| Lachnospira | rs56791201 | T | C | 0.052 | | 0.011 | 2.93E-06 | | -0.048 | 0.030 | 0.104 | 21.92 |
| Lachnospira | rs13157098 | A | G | -0.077 | | 0.016 | 5.99E-07 | | 0.103 | 0.038 | 0.006 | 24.46 |
| Ruminococcaceae UCG 002 | rs10916131 | C | T | -0.069 | | 0.015 | 2.87E-06 | | -0.025 | 0.039 | 0.517 | 22.32 |
| Ruminococcaceae UCG 002 | rs10927423 | C | A | -0.071 | | 0.015 | 8.50E-07 | | 0.001 | 0.037 | 0.983 | 23.34 |
| Ruminococcaceae UCG 002 | rs10964441 | G | A | -0.149 | | 0.034 | 7.45E-06 | | -0.046 | 0.047 | 0.324 | 18.68 |
| Ruminococcaceae UCG 002 | rs113147300 | A | G | -0.076 | | 0.016 | 7.69E-06 | | -0.021 | 0.042 | 0.618 | 21.24 |
| Ruminococcaceae UCG 002 | rs11607472 | A | G | -0.078 | | 0.018 | 7.19E-06 | | -0.093 | 0.057 | 0.100 | 19.58 |
| Ruminococcaceae UCG 002 | rs116974815 | C | A | -0.190 | | 0.040 | 2.03E-06 | | -0.083 | 0.056 | 0.137 | 22.89 |
| Ruminococcaceae UCG 002 | rs11750293 | G | T | -0.058 | | 0.012 | 1.76E-06 | | 0.054 | 0.030 | 0.071 | 23.03 |
| Ruminococcaceae UCG 002 | rs12463378 | A | G | -0.052 | | 0.011 | 2.96E-06 | | -0.044 | 0.031 | 0.153 | 21.69 |
| Ruminococcaceae UCG 002 | rs15256 | C | T | 0.073 | | 0.017 | 9.46E-06 | | -0.010 | 0.044 | 0.816 | 18.93 |
| Ruminococcaceae UCG 002 | rs55793120 | T | C | 0.137 | | 0.027 | 4.81E-07 | | 0.084 | 0.061 | 0.166 | 25.12 |
| Ruminococcaceae UCG 002 | rs57079348 | T | G | -0.077 | | 0.017 | 7.22E-06 | | -0.058 | 0.062 | 0.351 | 19.63 |
| Ruminococcaceae UCG 002 | rs6542556 | A | G | 0.051 | | 0.011 | 7.86E-06 | | -0.027 | 0.029 | 0.356 | 19.97 |
| Ruminococcaceae UCG 002 | rs6793778 | C | T | -0.056 | | 0.013 | 9.81E-06 | | 0.001 | 0.032 | 0.983 | 19.90 |
| Ruminococcaceae UCG 002 | rs7120052 | A | C | 0.062 | | 0.014 | 1.97E-06 | | -0.007 | 0.036 | 0.853 | 21.25 |
| Ruminococcaceae UCG 002 | rs7155595 | C | A | 0.057 | | 0.012 | 1.15E-06 | | 0.000 | 0.031 | 0.995 | 23.73 |
| Ruminococcaceae UCG 002 | rs7249614 | A | G | -0.049 | | 0.011 | 9.07E-06 | | -0.036 | 0.029 | 0.215 | 19.78 |
| Ruminococcaceae UCG 002 | rs76847269 | A | G | 0.164 | | 0.036 | 5.17E-06 | | 0.034 | 0.090 | 0.709 | 21.08 |
| Ruminococcaceae UCG 002 | rs77564310 | A | C | -0.071 | | 0.014 | 3.29E-07 | | 0.002 | 0.035 | 0.954 | 25.63 |
| Ruminococcaceae UCG 002 | rs79016051 | C | T | -0.089 | | 0.019 | 2.34E-06 | | -0.022 | 0.042 | 0.607 | 21.96 |
| Ruminococcaceae UCG 002 | rs882348 | A | G | -0.080 | | 0.018 | 5.45E-06 | | -0.040 | 0.044 | 0.367 | 20.06 |
| Ruminococcus torques group | rs10904297 | A | G | -0.168 | | 0.039 | 2.69E-06 | | 0.024 | 0.099 | 0.808 | 18.53 |
| Ruminococcus torques group | rs1475330 | T | C | 0.052 | | 0.012 | 8.13E-06 | | -0.026 | 0.033 | 0.444 | 19.61 |
| Ruminococcus torques group | rs10967781 | C | A | 0.051 | | 0.011 | 8.37E-06 | | 0.004 | 0.031 | 0.900 | 20.09 |
| Ruminococcus torques group | rs77034621 | T | G | -0.152 | | 0.034 | 6.07E-06 | | 0.127 | 0.114 | 0.268 | 20.36 |
| Ruminococcus torques group | rs4073731 | T | C | 0.065 | | 0.014 | 4.05E-06 | | -0.036 | 0.039 | 0.347 | 21.01 |
| Ruminococcus torques group | rs12434631 | A | G | 0.075 | | 0.015 | 2.77E-06 | | -0.049 | 0.047 | 0.291 | 23.71 |
| Ruminococcus torques group | rs35866622 | T | C | -0.061 | | 0.011 | 2.21E-08 | | 0.045 | 0.030 | 0.136 | 31.28 |
