# Supplementary material for: Investigating causal associations among gut microbiota, metabolites, and liver diseases: a Mendelian randomization study
Source: Front Endocrinol (Lausanne). 2023 Jul 5;14:1159148. doi: 10.3389/fendo.2023.1159148 (PMC10354516; doi:10.3389/fendo.2023.1159148)
Supplement: Supplementary file 5 [file Table_5.docx]

| Table S5. Association of genetically predicted remaining genera with viral hepatitis | | | | | | | | | |
| --- | --- | --- | --- | --- | --- | --- | --- | --- | --- |
| Genus | IVW | | |  | MR-Egger | |  | Weighted median | |
|  | IVs | OR(95% CI) | *p* value | | OR(95%CI) | *p* value | | OR(95%CI) | *p* value |
| Actinomyces | 7 | 0.979(0.730-1.313) | 0.889 | | 1.677(0.820-3.430) | 0.216 | | 1.003(0.690-1.458) | 0.987 |
| Adlercreutzia | 8 | 0.933(0.662-1.313) | 0.690 | | 0.704(0.137-3.610) | 0.688 | | 0.891(0.564-1.407) | 0.619 |
| Akkermansia | 11 | 1.092(0.807-1.478) | 0.567 | | 0.615(0.222-1.703) | 0.374 | | 1.073(0.708-1.626) | 0.739 |
| Allisonella | 8 | 0.996(0.827-1.199) | 0.967 | | 0.881(0.251-3.096) | 0.850 | | 1.020(0.803-1.296) | 0.873 |
| Alloprevotella | 5 | 0.964(0.762-1.221) | 0.764 | | 3.863(0.498-29.983) | 0.287 | | 0.943(0.683-1.302) | 0.721 |
| Anaerotruncus | 13 | 1.129(0.745-1.711) | 0.568 | | 0.757(0.219-2.620) | 0.669 | | 0.975(0.601-1.582) | 0.919 |
| Anaerofilum | 10 | 0.990(0.799-1.227) | 0.929 | | 1.495(0.465-4.804) | 0.519 | | 0.955(0.719-1.270) | 0.754 |
| Anaerostipes | 13 | 1.186(0.834-1.686) | 0.342 | | 0.839(0.228-3.085) | 0.797 | | 1.075(0.646-1.788) | 0.782 |
| Bacteroides | 8 | 1.261(0.676-2.353) | 0.466 | | 0.629(0.019-21.376) | 0.805 | | 1.565(0.828-2.958) | 0.168 |
| Barnesiella | 12 | 1.249( 0.850-1.836) | 0.258 | | 0.981(0.187-5.137) | 0.982 | | 1.552(0.968-2.488) | 0.068 |
| Bifidobacterium | 12 | 0.944(0.714-1.248) | 0.687 | | 0.958(0.479-1.917) | 0.907 | | 0.946(0.662-1.351) | 0.759 |
| Bilophila | 13 | 1.238(0.904-1.695) | 0.182 | | 1.765(0.375-8.315) | 0.487 | | 1.484(0.972- 2.268) | 0.068 |
| Blautia | 12 | 0.738(0.510-1.069) | 0.108 | | 1.204(0.461-3.145) | 0.712 | | 0.875(0.537-1.425) | 0.590 |
| Butyricicoccus | 8 | 0.911(0.620-1.338) | 0.633 | | 0.577(0.271-1.227) | 0.203 | | 0.827(0.504-1.357) | 0.453 |
| Butyricimonas | 13 | 0.773(0.562-1.061) | 0.111 | | 0.497(0.159-1.560) | 0.256 | | 0.701(0.470-1.045) | 0.081 |
| Butyrivibrio | 15 | 1.068(0.919-1.241) | 0.390 | | 1.360(0.704-2.624) | 0.376 | | 0.965(0.794-1.172) | 0.717 |
| Candidatus Soleaferrea | 9 | 1.012(0.718-1.428) | 0.944 | | 3.909(0.106-144.824) | 0.484 | | 0.997(0.694-1.432) | 0.986 |
| Catenibacterium | 4 | 0.942(0.712-1.245) | 0.672 | | 5.050(0.153-167.148) | 0.460 | | 0.972(0.688-1.372) | 0.871 |
| Christensenellaceae R.7 group | 8 | 0.951(0.568-1.592) | 0.847 | | 1.007(0.141-7.165) | 0.995 | | 0.725(0.393-1.338) | 0.304 |
| Clostridium sensu stricto 1 | 6 | 1.174(0.807-1.708) | 0.401 | | 1.107(0.401-3.055) | 0.853 | | 1.227(0.778-1.936) | 0.379 |
| Clostridium innocuum group | 8 | 0.914(0.743-1.125) | 0.396 | | 0.896(0.316-2.543) | 0.843 | | 0.908(0.696-1.184) | 0.475 |
| Collinsella | 9 | 1.410(0.839-2.370) | 0.194 | | 2.477(0.325-18.858) | 0.410 | | 1.745(0.958-3.177) | 0.069 |
| Coprobacter | 11 | 1.202(0.854-1.692) | 0.290 | | 2.780(0.768-10.060) | 0.154 | | 1.110(0.763-1.617) | 0.585 |
| Coprococcus 1 | 12 | 0.962(0.665-1.392) | 0.839 | | 1.230(0.468-3.232) | 0.684 | | 0.806(0.518-1.255) | 0.340 |
| Coprococcus 2 | 8 | 0.942(0.652-1.362) | 0.751 | | 0.236(0.013-4.286) | 0.367 | | 0.999(0.623-1.604) | 0.998 |
| Coprococcus 3 | 8 | 0.769(0.450-1.314) | 0.337 | | 12.813(1.075-152.745) | 0.090 | | 0.906(0.491-1.673) | 0.753 |
| Desulfovibrio | 10 | 0.955(0.655-1.392) | 0.810 | | 0.781(0.242-2.519) | 0.690 | | 0.806(0.520-1.248) | 0.333 |
| Defluviitaleaceae UCG 011 | 9 | 0.875(0.648-1.181) | 0.383 | | 0.756(0.239-2.385) | 0.647 | | 0.776(0.510-1.181) | 0.236 |
| Dialister | 10 | 0.953(0.664-1.369) | 0.794 | | 1.131(0.247-5.189) | 0.878 | | 1.131(0.247-5.189) | 0.936 |
| Dorea | 10 | 0.912(0.553-1.505) | 0.719 | | 0.912(0.553-1.505) | 0.563 | | 0.808(0.457-1.428) | 0.462 |
| Eggerthella | 9 | 1.214(0.957-1.540) | 0.111 | | 1.032(0.331-3.214) | 0.959 | | 1.062(0.778-1.449) | 0.704 |
| Eisenbergiella | 11 | 0.932(0.735-1.181) | 0.559 | | 0.465(0.078-2.784) | 0.423 | | 1.007(0.744-1.364) | 0.964 |
| Enterorhabdus | 6 | 0.906(0.643-1.278) | 0.574 | | 0.724(0.292-1.795) | 0.524 | | 0.869(0.558-1.355) | 0.536 |
| Erysipelatoclostridium | 15 | 0.900(0.689-1.176) | 0.442 | | 0.730(0.247-2.152) | 0.578 | | 0.945(0.667-1.338) | 0.749 |
| Erysipelotrichaceae UCG 003 | 16 | 1.321(0.942-1.851) | 0.106 | | 2.838(1.222-6.590) | 0.029 | | 1.128(0.752-1.691) | 0.562 |
| Eubacterium brachy group | 10 | 0.940(0.773-1.141) | 0.530 | | 0.989(0.453-2.160) | 0.978 | | 0.984(0.772-1.255) | 0.899 |
| Eubacterium coprostanoligenes group | 12 | 1.205(0.833-1.743) | 0.323 | | 0.699(0.171-2.865) | 0.630 | | 1.113(0.688-1.800) | 0.662 |
| Eubacterium eligens group | 6 | 1.082(0.650-1.802) | 0.761 | | 0.746(0.091-6.091) | 0.798 | | 0.870(0.471-1.608) | 0.658 |
| Eubacterium fissicatena group | 9 | 1.213(0.885-1.664) | 0.230 | | 0.730(0.130-4.089) | 0.731 | | 1.057(0.775-1.443) | 0.725 |
| Eubacterium hallii group | 14 | 1.121(0.807-1.559) | 0.495 | | 1.612(0.823-3.160) | 0.189 | | 1.404(0.946-2.083) | 0.092 |
| Eubacterium nodatum group | 11 | 0.879(0.726-1.064) | 0.185 | | 1.322(0.563-3.104) | 0.537 | | 0.892(0.697-1.140) | 0.360 |
| Eubacterium rectale group | 8 | 1.049(0.684-1.608) | 0.828 | | 3.213(0.675-15.298) | 0.193 | | 1.139(0.649-2.000) | 0.651 |
| Eubacterium ruminantium group | 18 | 1.208(0.996-1.464) | 0.055 | | 1.720(0.903-3.279) | 0.119 | | 1.128(0.858-1.482) | 0.388 |
| Eubacterium oxidoreducens group | 5 | 0.865(0.635-1.180) | 0.361 | | 0.957(0.303-3.018) | 0.945 | | 0.954(0.647-1.407) | 0.813 |
| Eubacterium ventriosum group | 15 | 1.128(0.834-1.524) | 0.435 | | 0.941(0.245-3.609) | 0.931 | | 1.379(0.914-2.081) | 0.126 |
| Eubacterium xylanophilum group | 9 | 1.073(0.642-1.793) | 0.788 | | 2.099(0.445-9.901) | 0.380 | | 1.051(0.640-1.725) | 0.846 |
| Faecalibacterium | 10 | 1.028(0.732-1.445) | 0.873 | | 1.056(0.523-2.133) | 0.882 | | 0.863(0.564-1.321) | 0.873 |
| Family XIII AD3011 group | 13 | 1.048(0.688-1.595) | 0.828 | | 0.768(0.097-6.100) | 0.807 | | 1.287(0.797-2.076) | 0.302 |
| Family XIII UCG 001 | 8 | 1.368(0.947-1.977) | 0.095 | | 1.501(0.475-4.743) | 0.515 | | 1.452(0.891-2.366) | 0.134 |
| Flavonifractor | 5 | 1.290(0.815-2.041) | 0.278 | | 0.438(0.079-2.435) | 0.415 | | 0.944(0.511-1.744) | 0.855 |
| Fusicatenibacter | 18 | 1.185(0.880-1.598) | 0.264 | | 0.693(0.226-2.121) | 0.529 | | 0.965(0.644-1.445) | 0.862 |
| Gordonibacter | 11 | 1.007(0.844-1.201) | 0.938 | | 1.227(0.575-2.620) | 0.610 | | 1.030(0.811-1.308) | 0.806 |
| Haemophilus | 9 | 1.042(0.802-1.353) | 0.760 | | 1.263(0.704-2.267) | 0.460 | | 1.040(0.740-1.460) | 0.823 |
| Holdemania | 14 | 0.974(0.743-1.276) | 0.847 | | 0.822(0.360-1.876) | 0.650 | | 1.194(0.835-1.707) | 0.332 |
| Holdemanella | 11 | 1.021(0.815-1.280) | 0.854 | | 1.022(0.536-1.950) | 0.948 | | 1.039(0.773-1.397) | 0.799 |
| Howardella | 9 | 0.966(0.770-1.212) | 0.766 | | 1.760(0.754-4.106) | 0.232 | | 1.095(0.851-1.409) | 0.483 |
| Hungatella | 5 | 1.137(0.793-1.630) | 0.485 | | 5.692(1.018-31.835) | 0.142 | | 1.300(0.901-1.876) | 0.161 |
| Intestinimonas | 16 | 0.909(0.705-1.172) | 0.462 | | 1.255(0.619-2.547) | 0.539 | | 0.927(0.656-1.309) | 0.665 |
| Intestinibacter | 14 | 0.923(0.700-1.218) | 0.572 | | 0.827(0.349-1.960) | 0.674 | | 0.978(0.664-1.441) | 0.912 |
| Lachnoclostridium | 13 | 1.129(0.802-1.590) | 0.486 | | 1.591(0.497-5.091) | 0.450 | | 1.270(0.786-2.050) | 0.329 |
| Lactobacillus | 8 | 1.109(0.773-1.591) | 0.574 | | 1.021(0.376-2.774) | 0.969 | | 1.114(0.768-1.616) | 0.569 |
| Lachnospira | 6 | 0.912(0.538-1.547) | 0.733 | | 4.825(0.212-109.806) | 0.379 | | 0.890(0.444-1.784) | 0.742 |
| Lachnospiraceae FCS020 group | 12 | 1.090(0.716-1.659) | 0.688 | | 1.090(0.716-1.659) | 0.454 | | 0.992(0.638-1.544) | 0.972 |
| Lachnospiraceae NC2004 group | 9 | 0.889(0.685-1.152) | 0.373 | | 0.889(0.685-1.152) | 0.989 | | 0.907(0.653-1.260) | 0.560 |
| Lachnospiraceae ND3007 group | 3 | 1.460(0.490-4.345) | 0.497 | | 0.084(0.0001-10000) | 0.880 | | 0.932(0.343-2.531) | 0.889 |
| Lachnospiraceae NK4A136 group | 15 | 1.143(0.872-1.497) | 0.334 | | 1.346(0.783-2.312) | 0.302 | | 1.376(0.929-2.037) | 0.111 |
| Lachnospiraceae UCG 001 | 13 | 1.183(0.908-1.543) | 0.214 | | 2.213(0.756-6.484) | 0.175 | | 1.252(0.870-1.801) | 0.226 |
| Lachnospiraceae UCG 004 | 12 | 0.890(0.633-1.251) | 0.503 | | 0.999(0.242-4.123) | 0.999 | | 0.892(0.567-1.404) | 0.622 |
| Lachnospiraceae UCG 008 | 10 | 1.006(0.792-1.277) | 0.963 | | 1.310(0.392-4.377) | 0.672 | | 1.037(0.770-1.396) | 0.813 |
| Lachnospiraceae UCG 010 | 10 | 1.016(0.712-1.451) | 0.928 | | 1.427(0.480-4.246) | 0.541 | | 0.963(0.607-1.528) | 0.872 |
| Lactococcus | 8 | 1.158(0.831-1.616) | 0.386 | | 2.493(0.545-11.403) | 0.284 | | 1.081(0.760-1.538) | 0.665 |
| Marvinbryantia | 10 | 1.266(0.864-1.856) | 0.226 | | 2.949(0.679-12.798) | 0.187 | | 1.104(0.685-1.779) | 0.686 |
| Methanobrevibacter | 6 | 0.926(0.694-1.235) | 0.599 | | 1.159(0.353-3.803) | 0.819 | | 0.915(0.644-1.300) | 0.620 |
| Odoribacter | 7 | 1.095(0.636-1.886) | 0.743 | | 0.482(0.088-2.646) | 0.439 | | 0.852(0.482-1.505) | 0.581 |
| Olsenella | 10 | 0.900(0.753-1.075) | 0.246 | | 0.949(0.530-1.700) | 0.866 | | 0.933(0.732-1.190) | 0.577 |
| Oscillibacter | 13 | 0.886(0.697-1.127) | 0.323 | | 0.985(0.385-2.518) | 0.975 | | 0.949(0.671-1.341) | 0.767 |
| Oscillospira | 8 | 0.912(0.655-1.271) | 0.588 | | 1.023(0.253-4.145) | 0.975 | | 0.788(0.518-1.198) | 0.265 |
| Oxalobacter | 11 | 1.055(0.876-1.271) | 0.571 | | 1.719(0.716-4.129) | 0.257 | | 1.094(0.847-1.413) | 0.493 |
| Parabacteroides | 5 | 0.781(0.450-1.353) | 0.377 | | 8.474(0.327-219.31) | 0.288 | | 1.033(0.516-2.068) | 0.926 |
| Paraprevotella | 13 | 0.996(0.810-1.225) | 0.970 | | 1.028(0.470-2.248) | 0.947 | | 1.025(0.786-1.336) | 0.858 |
| Parasutterella | 14 | 1.129(0.878-1.450) | 0.345 | | 1.475(0.728-2.989) | 0.301 | | 1.158(0.805-1.667) | 0.429 |
| Peptococcus | 12 | 1.108(0.864-1.421) | 0.417 | | 1.158(0.425-3.153) | 0.780 | | 1.204(0.906-1.601) | 0.200 |
| Phascolarctobacterium | 8 | 0.921(0.648-1.310) | 0.647 | | 0.387(0.075-1.991) | 0.299 | | 0.950(0.599-1.508) | 0.828 |
| Prevotella7 | 11 | 1.054(0.884-1.257) | 0.557 | | 1.635(0.588-4.549) | 0.371 | | 1.086(0.860-1.371) | 0.487 |
| Prevotella9 | 15 | 1.095(0.873-1.374) | 0.431 | | 1.695(0.876-3.281) | 0.141 | | 1.241(0.915-1.682) | 0.165 |
| Rikenellaceae RC9 gut group | 11 | 0.903(0.722-1.130) | 0.371 | | 1.164(0.269-5.032) | 0.843 | | 0.992(0.775-1.270) | 0.849 |
| Romboutsia | 13 | 1.037(0.764-1.408) | 0.815 | | 1.270(0.525-3.069) | 0.606 | | 1.045(0.686-1.593) | 0.837 |
| Roseburia | 14 | 0.824(0.588-1.157) | 0.264 | | 1.036(0.353-3.034) | 0.950 | | 0.995(0.622-1.591) | 0.982 |
| Ruminiclostridium 5 | 11 | 0.932(0.641-1.355) | 0.714 | | 2.302(0.483-10.978) | 0.323 | | 0.852(0.536-1.355) | 0.499 |
| Ruminiclostridium 6 | 15 | 0.890(0.610-1.298) | 0.545 | | 0.756(0.288-1.985) | 0.579 | | 0.848(0.553-1.299) | 0.448 |
| Ruminiclostridium 9 | 8 | 0.873(0.464-1.642) | 0.674 | | 1.825(0.074-44.788) | 0.725 | | 1.132(0.607-2.109) | 0.697 |
| Ruminococcus gauvreauii group | 11 | 0.796(0.498-1.272) | 0.341 | | 0.879(0.116-6.641) | 0.903 | | 0.975(0.629-1.510) | 0.910 |
| Ruminococcus gnavus group | 11 | 0.951(0.742-1.219) | 0.694 | | 0.600(0.178-2.021) | 0.431 | | 0.833(0.605-1.147) | 0.263 |
| Ruminococcus torques group | 7 | 0.999(0.602-1.655) | 0.996 | | 1.956(0.415-9.229) | 0.435 | | 0.941(0.477-1.855) | 0.860 |
| Ruminococcaceae UCG 002 | 20 | 1.267(0.989-1.624) | 0.061 | | 1.661(0.873-3.163) | 0.140 | | 1.381(0.977-1.954) | 0.068 |
| Ruminococcaceae UCG 003 | 12 | 0.767(0.498-1.180) | 0.228 | | 1.444(0.346-6.019) | 0.625 | | 0.610(0.384-0.972) | **0.037** |
| Ruminococcaceae UCG 004 | 11 | 0.917(0.694-1.211) | 0.541 | | 1.401(0.300-6.537) | 0.678 | | 0.989(0.683-1.430) | 0.951 |
| Ruminococcaceae UCG 005 | 14 | 1.025(0.753-1.397) | 0.874 | | 2.626(1.212-5.693) | **0.031** | | 1.006(0.666-1.520) | 0.977 |
| Ruminococcaceae UCG 009 | 11 | 0.929(0.729-1.182) | 0.548 | | 0.914(0.362-2.308) | 0.854 | | 0.932(0.677-1.284) | 0.668 |
| Ruminococcaceae UCG 010 | 6 | 0.792(0.529-1.187) | 0.259 | | 1.251(0.412-3.796) | 0.713 | | 0.844(0.499-1.428) | 0.528 |
| Ruminococcaceae UCG 011 | 8 | 0.862(0.695-1.071) | 0.180 | | 1.620(0.567-4.626) | 0.402 | | 0.907(0.696-1.181) | 0.468 |
| Ruminococcaceae UCG 013 | 11 | 1.346(0.950-1.908) | 0.094 | | 2.456(0.898-6.721) | 0.114 | | 1.549(0.974-2.466) | 0.065 |
| Ruminococcaceae UCG 014 | 10 | 1.002(0.731-1.374) | 0.991 | | 0.839(0.405-1.738) | 0.649 | | 1.057(0.679-1.646) | 0.806 |
| Ruminococcus 1 | 10 | 0.934(0.590-1.478) | 0.770 | | 1.499(0.431-5.211) | 0.542 | | 0.707(0.413-1.212) | 0.208 |
| Ruminococcus 2 | 15 | 1.198(0.904-1.587) | 0.209 | | 1.180(0.584-2.388) | 0.652 | | 1.148(0.799-1.649) | 0.454 |
| Sellimonas | 9 | 0.953(0.769-1.181) | 0.660 | | 2.337(0.724-7.545) | 0.199 | | 1.005(0.795-1.270) | 0.966 |
| Senegalimassilia | 5 | 1.171(0.797-1.720) | 0.421 | | 1.883(0.430-8.243) | 0.463 | | 1.190(0.758-1.866) | 0.450 |
| Slackia | 6 | 1.011(0.714-1.432) | 0.950 | | 0.230(0.029-1.804) | 0.234 | | 0.907(0.583-1.411) | 0.666 |
| Streptococcus | 12 | 1.126(0.750-1.690) | 0.568 | | 1.640(0.344-7.826) | 0.549 | | 1.153(0.702-1.894) | 0.573 |
| Subdoligranulum | 11 | 0.913(0.647-1.289) | 0.606 | | 0.581(0.239-1.415) | 0.262 | | 0.776(0.487-1.238) | 0.288 |
| Sutterella | 12 | 0.901(0.571-1.423) | 0.656 | | 3.681(0.568-23.865) | 0.202 | | 0.740(0.453-1.209) | 0.229 |
| Terrisporobacter | 5 | 1.013(0.705-1.454) | 0.946 | | 0.842(0.283-2.509) | 0.778 | | 1.027(0.668-1.580) | 0.903 |
| Turicibacter | 9 | 1.141(0.850-1.532) | 0.380 | | 2.553(0.774-8.416) | 0.167 | | 1.150(0.752-1.760) | 0.517 |
| Tyzzerella 3 | 12 | 1.044(0.852-1.279) | 0.677 | | 1.299(0.420-4.019) | 0.660 | | 0.979(0.754-1.271) | 0.873 |
| Veillonella | 5 | 1.029(0.682-1.552) | 0.891 | | 0.004(0.0001-51.867) | 0.335 | | 1.021(0.578-1.803) | 0.943 |
| Victivallis | 10 | 0.958(0.799-1.148) | 0.643 | | 0.982(0.246-3.921) | 0.980 | | 0.993(0.792-1.245) | 0.952 |
