# Supplementary material for: Investigating causal associations among gut microbiota, metabolites, and liver diseases: a Mendelian randomization study
Source: Front Endocrinol (Lausanne). 2023 Jul 5;14:1159148. doi: 10.3389/fendo.2023.1159148 (PMC10354516; doi:10.3389/fendo.2023.1159148)
Supplement: Supplementary file 6 [file Table_6.docx]

| Table S6. SNPs used as instrumental variables from gut microbiota and their associations with viral hepatitis | | | | | | | | | | | | |
| --- | --- | --- | --- | --- | --- | --- | --- | --- | --- | --- | --- | --- |
| Genus | SNP | Effect allele | Other allele | | gut microbiota | | | | viral hepatitis | | | F |
|  |  |  |  |  | Beta | SE | *p* value |  | Beta | SE | *p* value |  |
| Alistipes | rs1107244 | G | A | 0.076 | | 0.017 | 3.59E-06 | | 0.000 | 0.060 | 0.997 | 19.636 |
| Alistipes | rs11769002 | G | A | -0.053 | | 0.011 | 1.45E-06 | | -0.003 | 0.033 | 0.925 | 23.368 |
| Alistipes | rs11958296 | A | G | -0.098 | | 0.022 | 9.30E-06 | | -0.033 | 0.079 | 0.675 | 20.209 |
| Alistipes | rs12990744 | C | T | -0.078 | | 0.017 | 8.21E-06 | | -0.034 | 0.053 | 0.522 | 20.156 |
| Alistipes | rs1689282 | A | C | -0.052 | | 0.011 | 5.28E-06 | | -0.019 | 0.034 | 0.582 | 20.833 |
| Alistipes | rs2290844 | C | T | 0.081 | | 0.019 | 9.10E-06 | | 0.080 | 0.051 | 0.115 | 18.042 |
| Alistipes | rs2450745 | A | C | -0.081 | | 0.018 | 7.12E-06 | | -0.109 | 0.063 | 0.085 | 19.007 |
| Alistipes | rs2875322 | T | C | -0.058 | | 0.013 | 8.78E-06 | | -0.073 | 0.043 | 0.091 | 19.537 |
| Alistipes | rs34417064 | A | G | -0.048 | | 0.011 | 7.01E-06 | | -0.005 | 0.032 | 0.866 | 20.344 |
| Alistipes | rs4810359 | A | G | -0.065 | | 0.015 | 7.50E-06 | | -0.007 | 0.049 | 0.889 | 19.927 |
| Alistipes | rs7129639 | C | A | -0.052 | | 0.011 | 1.78E-06 | | -0.037 | 0.034 | 0.276 | 22.948 |
| Alistipes | rs8130320 | A | G | -0.049 | | 0.011 | 4.84E-06 | | -0.041 | 0.033 | 0.212 | 20.906 |
| Ruminococcaceae NK4A214 group | rs11241747 | C | T | 0.053 | | 0.012 | 6.59E-06 | | 0.012 | 0.036 | 0.728 | 19.781 |
| Ruminococcaceae NK4A216 group | rs11586410 | G | A | -0.086 | | 0.017 | 3.66E-07 | | -0.026 | 0.044 | 0.559 | 25.815 |
| Ruminococcaceae NK4A217 group | rs12642039 | T | C | -0.055 | | 0.012 | 3.43E-06 | | -0.044 | 0.033 | 0.191 | 21.452 |
| Ruminococcaceae NK4A218 group | rs12731 | A | G | -0.053 | | 0.012 | 4.87E-06 | | 0.021 | 0.033 | 0.527 | 21.035 |
| Ruminococcaceae NK4A219 group | rs13087692 | T | G | 0.057 | | 0.013 | 8.69E-06 | | 0.029 | 0.035 | 0.411 | 20.818 |
| Ruminococcaceae NK4A220 group | rs136761 | G | A | -0.059 | | 0.012 | 8.15E-07 | | -0.036 | 0.034 | 0.285 | 24.312 |
| Ruminococcaceae NK4A221 group | rs147475196 | A | G | -0.134 | | 0.030 | 4.72E-06 | | -0.057 | 0.053 | 0.283 | 20.535 |
| Ruminococcaceae NK4A223 group | rs35559912 | T | C | -0.093 | | 0.020 | 4.89E-06 | | -0.010 | 0.050 | 0.833 | 20.629 |
| Ruminococcaceae NK4A224 group | rs4814689 | C | T | -0.108 | | 0.023 | 4.55E-06 | | 0.032 | 0.077 | 0.677 | 22.036 |
| Ruminococcaceae NK4A225 group | rs5994253 | A | G | -0.081 | | 0.016 | 2.35E-07 | | -0.083 | 0.046 | 0.075 | 26.493 |
| Ruminococcaceae NK4A226 group | rs62027366 | T | C | 0.062 | | 0.014 | 6.58E-06 | | 0.063 | 0.040 | 0.118 | 19.998 |
| Ruminococcaceae NK4A227 group | rs6681678 | C | T | -0.100 | | 0.024 | 9.05E-06 | | 0.073 | 0.090 | 0.416 | 17.422 |
| Ruminococcaceae NK4A229 group | rs7573569 | T | C | 0.108 | | 0.023 | 3.23E-06 | | 0.054 | 0.067 | 0.427 | 21.264 |
