# Supplementary material for: Investigating causal associations among gut microbiota, metabolites, and liver diseases: a Mendelian randomization study
Source: Front Endocrinol (Lausanne). 2023 Jul 5;14:1159148. doi: 10.3389/fendo.2023.1159148 (PMC10354516; doi:10.3389/fendo.2023.1159148)
Supplement: Supplementary file 7 [file Table_7.docx]

| Table S7. Association of genetically predicted gut microbiota derived metabolites with non-alcoholic fatty liver disease | | | | | | | |
| --- | --- | --- | --- | --- | --- | --- | --- |
| Methods | IVs | OR | 95% CI | *p* value | Egger intercept, *p* value | Heterogeneity (Q, *p* value) | MR-PRESSO (Global test *p* value) |
| Betaine | | | | | | | |
| IVW | 23 | 1.648 | 0.750-3.618 | 0.2134 | -0.019,  0.440 | 16.141, 0.809 | 0.835 |
| Weighted median | 23 | 1.724 | 0.600-4.950 | 0.3118 |  |  |  |
| MR-Egger | 23 | 5.750 | 0.232-142.661 | 0.2978 |  |  |  |
| MR-PRESSO | 23 | 1.648 | 0.840-3.232 | 0.1604 |  |  |  |
| Carnitine |  |  |  |  |  |  |  |
| IVW | 149 | 1.020 | 0.474-2.194 | 0.9601 | 0.001, 0.874 | 137.005, 0.731 | 0.754 |
| Weighted median | 149 | 1.365 | 0.407-4.583 | 0.6145 |  |  |  |
| MR-Egger | 149 | 0.801 | 0.037-17.536 | 0.8882 |  |  |  |
| MR-PRESSO | 149 | 1.020 | 0.488-2.131 | 0.9586 |  |  |  |
| Cholate |  |  |  |  |  |  |  |
| IVW | 8 | 1.077 | 0.769-1.508 | 0.6671 | -0.035, 0.432 | 4.518, 0.7026 |  |
| Weighted median | 8 | 0.964 | 0.629-1.479 | 0.8671 |  |  | 0.664 |
| MR-Egger | 8 | 1.834 | 0.508-6.618 | 0.3901 |  |  |  |
| MR-PRESSO | 8 | 1.077 | 0.821-1.412 | 0.6089 |  |  |  |
| Choline |  |  |  |  |  |  |  |
| IVW | 18 | 2.593 | 0.182-36.851 | 0.4817 | 0.035, 0.314 | 21.254, 0.215 |  |
| Weighted median | 18 | 2.406 | 0.062-93.649 | 0.6383 |  |  | 0.245 |
| MR-Egger | 18 | 0.094 | 0.001-83.846 | 0.5048 |  |  |  |
| MR-PRESSO | 18 | 2.593 | 0.182-36.851 | 0.4912 |  |  |  |
| Phenylacetate |  |  |  |  |  |  |  |
| IVW | 8 | 0.902 | 0.584-1.390 | 0.6391 | 0.006, 0.796 | 7.997, 0.333 |  |
| Weighted median | 8 | 0.962 | 0.588-1.576 | 0.8785 |  |  | 0.545 |
| MR-Egger | 8 | 0.715 | 0.125-4.082 | 0.7189 |  |  |  |
| MR-PRESSO | 8 | 0.902 | 0.584-1.390 | 0.6533 |  |  |  |
| Threonate |  |  |  |  |  |  |  |
| IVW | 16 | 1.022 | 0.486-2.148 | 0.9542 | -0.005,  0.765 | 13.940, 0.530 |  |
| Weighted median | 16 | 1.342 | 0.468-3.842 | 0.5841 |  |  | 0.584 |
| MR-Egger | 16 | 1.306 | 0.228-7.477 | 0.7688 |  |  |  |
| MR-PRESSO | 16 | 1.022 | 0.450-2.091 | 0.9533 |  |  |  |
| Ursodeoxycholate |  |  |  |  |  |  |  |
| IVW | 7 | 1.151 | 0.643-2.057 | 0.6361 | 0.013, 0.692 | 2.356, 0.884 |  |
| Weighted median | 7 | 1.073 | 0.517-2.227 | 0.8493 |  |  | 0.858 |
| MR-Egger | 7 | 0.936 | 0.304-2.880 | 0.9131 |  |  |  |
| MR-PRESSO | 7 | 1.151 | 0.799-1.656 | 0.4788 |  |  |  |
