# Supplementary material for: Investigating causal associations among gut microbiota, metabolites, and liver diseases: a Mendelian randomization study
Source: Front Endocrinol (Lausanne). 2023 Jul 5;14:1159148. doi: 10.3389/fendo.2023.1159148 (PMC10354516; doi:10.3389/fendo.2023.1159148)
Supplement: Supplementary file 8 [file Table_8.docx]

| Table S8. Association of genetically predicted gut microbiota derived metabolites with alcoholic liver disease | | | | | | | |
| --- | --- | --- | --- | --- | --- | --- | --- |
| Methods | IVs | OR | 95% CI | *p* value | Egger intercept, *p* value | Heterogeneity (Q, *p* value) | MR-PRESSO (Global test *p* value) |
| Alanine | | | | | | | |
| IVW | 37 | 0.922 | 0.283-3.005 | 0.8926 | -0.026,  0.240 | 47.981, 0.087 | 0.098 |
| Weighted median | 37 | 2.166 | 0.467-10.049 | 0.3238 |  |  |  |
| MR-Egger | 37 | 12.121 | 0.151-971.310 | 0.2722 |  |  |  |
| MR-PRESSO | 37 | 0.922 | 0.283-3.005 | 0.8933 |  |  |  |
| Betaine |  |  |  |  |  |  |  |
| IVW | 21 | 0.487 | 0.169-1.400 | 0.1817 | -0.003, 0.880 | 25.954, 0.167 |  |
| Weighted median | 21 | 0.708 | 0.191-2.624 | 0.6055 |  |  | 0.190 |
| MR-Egger | 21 | 0.577 | 0.051-6.579 | 0.6630 |  |  |  |
| MR-PRESSO | 21 | 0.487 | 0.169-1.400 | 0.1967 |  |  |  |
| Carnitine |  |  |  |  |  |  |  |
| IVW | 159 | 1.518 | 0.564-4.082 | 0.4087 | 0.009, 0.287 | 178.010, 0.132 |  |
| Weighted median | 159 | 1.352 | 0.289-6.322 | 0.7016 |  |  | 0.127 |
| MR-Egger | 159 | 0.300 | 0.013-6.897 | 0.4529 |  |  |  |
| MR-PRESSO | 159 | 1.518 | 0.564-4.082 | 0.4099 |  |  |  |
| Choline |  |  |  |  |  |  |  |
| IVW | 23 | 0.309 | 0.041-2.340 | 0.2558 | -0.025, 0.353 | 28.985, 0.145 |  |
| Weighted median | 23 | 0.273 | 0.019-4.006 | 0.3434 |  |  | 0.146 |
| MR-Egger | 23 | 3.942 | 0.142-1092.25 | 0.6376 |  |  |  |
| MR-PRESSO | 23 | 0.309 | 0.041-2.340 | 0.2680 |  |  |  |
| Cholate |  |  |  |  |  |  |  |
| IVW | 9 | 0.945 | 0.724-1.233 | 0.6754 | 0.017, 0.434 | 5.744, 0.676 |  |
| Weighted median | 9 | 0.914 | 0.634-1.317 | 0.6280 |  |  | 0.703 |
| MR-Egger | 9 | 0.790 | 0.480-1.302 | 0.3857 |  |  |  |
| MR-PRESSO | 9 | 0.945 | 0.754-1.184 | 0.6345 |  |  |  |
| Phenyllactate |  |  |  |  |  |  |  |
| IVW | 18 | 0.472 | 0.181-1.230 | 0.1245 | -0.009, 0.704 | 19.083, 0.323 |  |
| Weighted median | 18 | 0.503 | 0.132-1.917 | 0.3140 |  |  | 0.338 |
| MR-Egger | 18 | 0.759 | 0.056-10.327 | 0.8388 |  |  |  |
| MR-PRESSO | 18 | 0.472 | 0.181-1.230 | 0.1429 |  |  |  |
| Stachydrine |  |  |  |  |  |  |  |
| IVW | 7 | 1.027 | 0.733-1.440 | 0.8769 | 0.085, 0.193 | 5.998, 0.423 |  |
| Weighted median | 7 | 1.098 | 0.698-1.727 | 0.6849 |  |  | 0.468 |
| MR-Egger | 7 | 0.373 | 0.095-1.459 | 0.2156 |  |  |  |
| MR-PRESSO | 7 | 1.027 | 0.733-1.440 | 0.8819 |  |  |  |
