# Supplementary material for: Investigating causal associations among gut microbiota, metabolites, and liver diseases: a Mendelian randomization study
Source: Front Endocrinol (Lausanne). 2023 Jul 5;14:1159148. doi: 10.3389/fendo.2023.1159148 (PMC10354516; doi:10.3389/fendo.2023.1159148)
Supplement: Supplementary file 9 [file Table_9.docx]

| Table S9. Association of genetically predicted gut microbiota derived metabolites with viral hepatitis | | | | | | | |
| --- | --- | --- | --- | --- | --- | --- | --- |
| Methods | IVs | OR | 95% CI | *p* value | Egger intercept, *p* value | Heterogeneity (Q, *p* value) | MR-PRESSO (Global test *p* value) |
| Betaine | | | | | | | |
| IVW | 21 | 1.454 | 0.456-4.639 | 0.5266 | -0.017,  0.457 | 24.674, 0.214 | 0.231 |
| Weighted median | 21 | 1.866 | 0.368-9.477 | 0.4516 |  |  |  |
| MR-Egger | 21 | 3.610 | 0.262-49.738 | 0.3495 |  |  |  |
| MR-PRESSO | 21 | 1.454 | 0.456-4.639 | 0.5338 |  |  |  |
| Carnitine |  |  |  |  |  |  |  |
| IVW | 159 | 0.843 | 0.294-2.420 | 0.7515 | 0.010, 0.226 | 134.901, 0.908 |  |
| Weighted median | 159 | 0.422 | 0.082-2.181 | 0.3031 |  |  | 0.909 |
| MR-Egger | 159 | 0.118 | 0.004-3.3236 | 0.2116 |  |  |  |
| MR-PRESSO | 159 | 0.843 | 0.318-2.234 | 0.7323 |  |  |  |
| Choline |  |  |  |  |  |  |  |
| IVW | 23 | 0.149 | 0.013-1.751 | 0.1299 | -0.001, 0.997 | 33.617, 0.054 |  |
| Weighted median | 23 | 0.432 | 0.022-8.372 | 0.5789 |  |  | 0.062 |
| MR-Egger | 23 | 0.151 | 0.001-169.102 | 0.6034 |  |  |  |
| MR-PRESSO | 23 | 0.149 | 0.013-1.751 | 0.1441 |  |  |  |
| Phenylacetate |  |  |  |  |  |  |  |
| IVW | 9 | 1.101 | 0.524-2.310 | 0.7998 | -0.021, 0.418 | 4.605, 0.799 |  |
| Weighted median | 9 | 1.406 | 0.557-3.548 | 0.4703 |  |  | 0.673 |
| MR-Egger | 9 | 1.521 | 0.535-4.325 | 0.4574 |  |  |  |
| MR-PRESSO | 9 | 1.101 | 0.627-1.931 | 0.7468 |  |  |  |
| Phenyllactate |  |  |  |  |  |  |  |
| IVW | 18 | 0.586 | 0.200-1.717 | 0.3303 | -0.042, 0.117 | 18.736, 0.344 |  |
| Weighted median | 18 | 1.078 | 0.253-4.593 | 0.9194 |  |  | 0.380 |
| MR-Egger | 18 | 4.896 | 0.325-73.751 | 0.2679 |  |  |  |
| MR-PRESSO | 18 | 0.586 | 0.200-1.717 | 0.3440 |  |  |  |
| Stachydrine |  |  |  |  |  |  |  |
| IVW | 7 | 0.919 | 0.588-1.437 | 0.7122 | 0.021, 0.802 | 8.209, 0.223 |  |
| Weighted median | 7 | 1.071 | 0.626-1.831 | 0.8035 |  |  | 0.234 |
| MR-Egger | 7 | 0.712 | 0.100-5.058 | 0.7480 |  |  |  |
| MR-PRESSO | 7 | 0.919 | 0.588-1.437 | 0.7248 |  |  |  |
| Ursodeoxycholate |  |  |  |  |  |  |  |
| IVW | 11 | 0.918 | 0.621-1.356 | 0.6666 | 0.011, 0.641 | 10.905, 0.365 |  |
| Weighted median | 11 | 0.880 | 0.541-1.431 | 0.6057 |  |  | 0.411 |
| MR-Egger | 11 | 0.798 | 0.396-1.606 | 0.5425 |  |  |  |
| MR-PRESSO | 11 | 0.917 | 0.621-1.356 | 0.6757 |  |  |  |
