# Supplementary material for: Investigating causal associations among gut microbiota, metabolites, and liver diseases: a Mendelian randomization study
Source: Front Endocrinol (Lausanne). 2023 Jul 5;14:1159148. doi: 10.3389/fendo.2023.1159148 (PMC10354516; doi:10.3389/fendo.2023.1159148)
Supplement: Supplementary file 10 [file Table_10.docx]

| Table S10. SNPs used as instrumental variables from gut microbiota-derived metabolites and their associations with non-alcoholic fatty liver disease | | | | | | | | | | | | |
| --- | --- | --- | --- | --- | --- | --- | --- | --- | --- | --- | --- | --- |
| Metabolite**s** | SNP | Effect allele | Other allele | | metabolite**s** | | | | NAFLD | | | F |
|  |  |  |  |  | Beta | SE | *p* value |  | Beta | SE | *p* value |  |
| Alanine | rs10211524 | A | G | -0.010 | | 0.002 | 7.99E-06 | | -0.028 | 0.102 | 0.782 | 20.446 |
| Alanine | rs11183620 | A | G | 0.010 | | 0.002 | 1.55E-08 | | 0.054 | 0.036 | 0.141 | 33.232 |
| Alanine | rs11652554 | T | C | 0.009 | | 0.002 | 1.52E-06 | | 0.048 | 0.042 | 0.253 | 23.361 |
| Alanine | rs11704957 | A | C | -0.023 | | 0.005 | 2.85E-06 | | -0.105 | 0.096 | 0.274 | 22.224 |
| Alanine | rs11893991 | A | G | 0.009 | | 0.002 | 8.89E-07 | | -0.068 | 0.043 | 0.111 | 24.448 |
| Alanine | rs1260326 | T | C | 0.013 | | 0.002 | 5.56E-14 | | 0.245 | 0.038 | 0.000 | 59.381 |
| Alanine | rs1289671 | A | G | -0.008 | | 0.002 | 1.56E-06 | | 0.063 | 0.038 | 0.098 | 21.778 |
| Alanine | rs1440327 | T | C | -0.008 | | 0.002 | 6.00E-06 | | -0.033 | 0.042 | 0.429 | 20.753 |
| Alanine | rs1566951 | T | C | -0.008 | | 0.002 | 4.52E-06 | | -0.042 | 0.069 | 0.541 | 20.753 |
| Alanine | rs17106647 | T | C | 0.028 | | 0.006 | 8.85E-07 | | -0.026 | 0.109 | 0.812 | 24.476 |
| Alanine | rs17520130 | T | C | -0.009 | | 0.002 | 6.85E-07 | | 0.066 | 0.046 | 0.154 | 23.901 |
| Alanine | rs1850267 | T | C | 0.008 | | 0.002 | 9.56E-06 | | 0.070 | 0.035 | 0.048 | 19.262 |
| Alanine | rs2064272 | A | G | -0.008 | | 0.002 | 4.49E-06 | | -0.003 | 0.050 | 0.956 | 20.753 |
| Alanine | rs211962 | T | C | -0.012 | | 0.003 | 5.31E-06 | | 0.010 | 0.473 | 0.983 | 21.302 |
| Alanine | rs2295689 | A | G | -0.009 | | 0.002 | 6.80E-06 | | 0.004 | 0.056 | 0.947 | 20.250 |
| Alanine | rs4317609 | A | G | -0.008 | | 0.002 | 7.98E-06 | | -0.021 | 0.326 | 0.949 | 19.262 |
| Alanine | rs465152 | A | G | -0.009 | | 0.002 | 6.45E-07 | | -0.095 | 0.040 | 0.018 | 26.190 |
| Alanine | rs485878 | A | G | -0.008 | | 0.002 | 4.67E-06 | | -0.046 | 0.050 | 0.351 | 20.250 |
| Alanine | rs4980554 | A | G | 0.008 | | 0.002 | 2.87E-06 | | 0.050 | 0.042 | 0.234 | 23.266 |
| Alanine | rs6053126 | A | G | -0.014 | | 0.003 | 3.06E-06 | | -0.018 | 0.026 | 0.480 | 21.778 |
| Alanine | rs6497167 | T | G | 0.008 | | 0.002 | 7.64E-06 | | 0.016 | 0.406 | 0.968 | 19.262 |
| Alanine | rs651158 | A | G | 0.009 | | 0.002 | 8.12E-07 | | -0.047 | 0.036 | 0.195 | 23.361 |
| Alanine | rs6706043 | T | G | -0.008 | | 0.002 | 7.33E-06 | | -0.093 | 0.046 | 0.044 | 19.262 |
| Alanine | rs6839120 | A | G | 0.008 | | 0.002 | 4.03E-06 | | 0.023 | 0.038 | 0.547 | 20.753 |
| Alanine | rs6962350 | T | G | 0.008 | | 0.002 | 5.68E-06 | | 0.049 | 0.033 | 0.143 | 19.753 |
| Alanine | rs7191435 | T | C | 0.008 | | 0.002 | 2.53E-06 | | 0.070 | 0.037 | 0.062 | 21.778 |
| Alanine | rs7712111 | A | G | 0.008 | | 0.002 | 1.63E-06 | | 0.008 | 0.020 | 0.697 | 21.778 |
| Alanine | rs7791060 | A | G | -0.017 | | 0.003 | 6.96E-07 | | 0.059 | 0.115 | 0.606 | 24.125 |
| Alanine | rs785896 | A | G | 0.009 | | 0.002 | 6.80E-06 | | 0.012 | 0.048 | 0.794 | 20.250 |
| Alanine | rs890230 | T | C | 0.009 | | 0.002 | 1.06E-06 | | -0.020 | 0.065 | 0.757 | 22.827 |
| Alanine | rs9367164 | A | G | 0.008 | | 0.002 | 9.17E-06 | | 0.026 | 0.066 | 0.700 | 20.516 |
| Alanine | rs9582849 | A | G | 0.020 | | 0.004 | 1.34E-06 | | 0.046 | 0.095 | 0.628 | 23.795 |
| Alanine | rs9876174 | T | C | 0.012 | | 0.003 | 7.00E-06 | | -0.058 | 0.049 | 0.235 | 20.250 |
| Phenyllactate | rs12155900 | T | C | -0.017 | | 0.004 | 7.34E-06 | | 0.024 | 0.034 | 0.480 | 19.905 |
| Phenyllactate | rs12355427 | A | G | -0.012 | | 0.003 | 9.20E-06 | | 0.044 | 0.072 | 0.543 | 20.084 |
| Phenyllactate | rs12637114 | T | C | 0.026 | | 0.006 | 9.65E-06 | | -0.064 | 0.039 | 0.097 | 19.720 |
| Phenyllactate | rs12970549 | A | G | -0.012 | | 0.003 | 8.16E-06 | | -0.153 | 0.061 | 0.012 | 19.612 |
| Phenyllactate | rs13415138 | T | C | -0.013 | | 0.003 | 2.90E-06 | | 0.031 | 0.049 | 0.537 | 22.827 |
| Phenyllactate | rs1471834 | A | G | 0.014 | | 0.003 | 1.45E-06 | | -0.076 | 0.060 | 0.201 | 23.592 |
| Phenyllactate | rs17042584 | T | G | 0.013 | | 0.003 | 4.75E-06 | | -0.022 | 0.057 | 0.703 | 20.573 |
| Phenyllactate | rs1821685 | T | C | 0.013 | | 0.003 | 1.71E-06 | | 0.004 | 0.022 | 0.856 | 23.182 |
| Phenyllactate | rs2032995 | T | G | 0.013 | | 0.003 | 1.35E-06 | | -0.049 | 0.029 | 0.095 | 23.901 |
| Phenyllactate | rs4637923 | T | C | -0.012 | | 0.003 | 9.42E-06 | | 0.031 | 0.044 | 0.492 | 19.753 |
| Phenyllactate | rs4784054 | A | G | 0.035 | | 0.004 | 2.31E-21 | | -0.029 | 0.059 | 0.630 | 88.971 |
| Phenyllactate | rs4799200 | A | G | 0.025 | | 0.006 | 3.79E-06 | | -0.147 | 0.094 | 0.118 | 20.993 |
| Phenyllactate | rs4872094 | T | C | -0.033 | | 0.007 | 8.22E-06 | | 0.041 | 0.050 | 0.418 | 20.065 |
| Phenyllactate | rs6966840 | A | G | 0.013 | | 0.003 | 2.01E-06 | | -0.144 | 0.042 | 0.001 | 22.563 |
| Phenyllactate | rs747902 | A | G | -0.013 | | 0.003 | 2.13E-06 | | 0.027 | 0.049 | 0.576 | 22.827 |
| Phenyllactate | rs7689395 | T | C | -0.025 | | 0.005 | 2.34E-06 | | 0.049 | 0.084 | 0.559 | 22.380 |
| Phenyllactate | rs9467632 | A | G | -0.018 | | 0.004 | 8.50E-07 | | 0.015 | 0.017 | 0.377 | 24.462 |
| Stachydrine | rs17630756 | A | G | 0.177 | | 0.039 | 5.99E-06 | | 0.222 | 0.124 | 0.073 | 20.446 |
| Stachydrine | rs2688892 | T | C | 0.069 | | 0.015 | 4.47E-06 | | 0.131 | 0.054 | 0.015 | 21.038 |
| Stachydrine | rs4708385 | A | G | -0.061 | | 0.013 | 5.20E-06 | | 0.023 | 0.093 | 0.807 | 20.898 |
| Stachydrine | rs7975440 | T | C | -0.089 | | 0.019 | 2.31E-06 | | 0.015 | 2.091 | 0.994 | 22.398 |
| Stachydrine | rs9319364 | T | C | 0.092 | | 0.021 | 8.75E-06 | | 0.050 | 0.049 | 0.313 | 19.710 |
| Stachydrine | rs9443691 | A | G | -0.081 | | 0.018 | 8.68E-06 | | 0.063 | 0.088 | 0.476 | 19.759 |
