# Supplementary material for: Investigating causal associations among gut microbiota, metabolites, and liver diseases: a Mendelian randomization study
Source: Front Endocrinol (Lausanne). 2023 Jul 5;14:1159148. doi: 10.3389/fendo.2023.1159148 (PMC10354516; doi:10.3389/fendo.2023.1159148)
Supplement: Supplementary file 11 [file Table_11.docx]

| Table S11. SNPs used as instrumental variables from gut microbiota-derived metabolites and their associations with alcoholic liver disease | | | | | | | | | | | | |
| --- | --- | --- | --- | --- | --- | --- | --- | --- | --- | --- | --- | --- |
| Metabolites | SNP | Effect allele | Other allele | | metabolites | | | | ALD | | | F |
|  |  |  |  |  | Beta | SE | *p* value |  | Beta | SE | *p* value |  |
| Phenylacetate | rs10520388 | T | G | 0.022 | | 0.005 | 4.92E-06 | | 0.013 | 0.046 | 0.779 | 20.816 |
| Phenylacetate | rs16885269 | T | C | 0.026 | | 0.006 | 8.07E-06 | | -0.002 | 0.043 | 0.957 | 20.014 |
| Phenylacetate | rs17683199 | A | G | -0.045 | | 0.009 | 1.88E-06 | | 0.098 | 0.084 | 0.242 | 22.512 |
| Phenylacetate | rs201060 | A | G | 0.037 | | 0.008 | 9.75E-06 | | 0.054 | 0.074 | 0.471 | 19.551 |
| Phenylacetate | rs2133896 | T | G | 0.047 | | 0.010 | 2.36E-06 | | -0.043 | 0.078 | 0.584 | 22.443 |
| Phenylacetate | rs2345834 | T | C | 0.018 | | 0.004 | 5.45E-06 | | -0.022 | 0.030 | 0.455 | 20.366 |
| Phenylacetate | rs2579931 | A | G | 0.027 | | 0.006 | 4.23E-06 | | -0.018 | 0.060 | 0.766 | 20.787 |
| Phenylacetate | rs4296931 | A | G | 0.152 | | 0.033 | 5.10E-06 | | -0.141 | 0.060 | 0.019 | 20.793 |
| Phenylacetate | rs7580111 | A | T | 0.021 | | 0.005 | 4.94E-06 | | 0.028 | 0.029 | 0.332 | 20.538 |
| Threonate | rs10097949 | A | G | 0.048 | | 0.010 | 3.41E-06 | | 0.039 | 0.053 | 0.463 | 21.686 |
| Threonate | rs10512185 | T | C | 0.017 | | 0.004 | 3.32E-06 | | -0.013 | 0.030 | 0.664 | 21.262 |
| Threonate | rs11914582 | T | C | -0.031 | | 0.007 | 2.89E-06 | | -0.010 | 0.030 | 0.746 | 21.637 |
| Threonate | rs12447958 | A | G | 0.019 | | 0.004 | 7.57E-06 | | 0.020 | 0.030 | 0.505 | 20.036 |
| Threonate | rs12779976 | A | G | 0.035 | | 0.007 | 1.26E-06 | | 0.013 | 0.054 | 0.804 | 23.227 |
| Threonate | rs13163920 | T | C | 0.016 | | 0.004 | 9.51E-06 | | -0.020 | 0.030 | 0.511 | 19.753 |
| Threonate | rs2257061 | T | C | -0.016 | | 0.004 | 6.33E-06 | | 0.014 | 0.030 | 0.631 | 20.250 |
| Threonate | rs2303010 | T | C | -0.147 | | 0.031 | 2.26E-06 | | -0.091 | 0.084 | 0.278 | 22.372 |
| Threonate | rs306472 | T | C | 0.020 | | 0.004 | 6.69E-06 | | -0.004 | 0.038 | 0.918 | 20.046 |
| Threonate | rs3766606 | T | G | 0.021 | | 0.005 | 4.93E-06 | | 0.017 | 0.032 | 0.603 | 21.160 |
| Threonate | rs576266 | T | C | -0.028 | | 0.006 | 5.97E-06 | | -0.022 | 0.037 | 0.561 | 20.321 |
| Threonate | rs7132285 | T | C | 0.137 | | 0.030 | 6.12E-06 | | 0.118 | 0.050 | 0.018 | 20.473 |
| Threonate | rs7216075 | A | G | -0.022 | | 0.004 | 4.39E-07 | | -0.010 | 0.032 | 0.744 | 25.000 |
| Threonate | rs7332927 | A | G | 0.138 | | 0.030 | 3.37E-06 | | -0.060 | 0.101 | 0.550 | 21.569 |
| Threonate | rs7993666 | T | C | 0.021 | | 0.005 | 3.67E-06 | | 0.021 | 0.037 | 0.567 | 21.160 |
| Threonate | rs8031609 | T | G | 0.020 | | 0.004 | 7.45E-06 | | 0.005 | 0.036 | 0.881 | 20.250 |
| Threonate | rs892429 | A | G | 0.084 | | 0.019 | 9.86E-06 | | 0.011 | 0.051 | 0.834 | 19.499 |
| Threonate | rs9572687 | A | G | -0.016 | | 0.004 | 6.55E-06 | | 0.026 | 0.032 | 0.428 | 20.250 |
| Ursodeoxycholate | rs10174534 | A | G | -0.028 | | 0.006 | 7.57E-06 | | 0.007 | 0.029 | 0.806 | 19.753 |
| Ursodeoxycholate | rs11736497 | T | C | 0.115 | | 0.025 | 5.56E-06 | | -0.068 | 0.064 | 0.289 | 20.681 |
| Ursodeoxycholate | rs12587564 | A | G | 0.046 | | 0.010 | 2.63E-06 | | 0.000 | 0.055 | 0.996 | 22.100 |
| Ursodeoxycholate | rs17118371 | T | C | 0.034 | | 0.007 | 1.44E-06 | | 0.013 | 0.031 | 0.670 | 23.338 |
| Ursodeoxycholate | rs2421668 | A | G | 0.034 | | 0.007 | 2.87E-06 | | -0.066 | 0.030 | 0.028 | 21.693 |
| Ursodeoxycholate | rs2720445 | T | C | 0.033 | | 0.007 | 5.56E-06 | | -0.012 | 0.029 | 0.669 | 20.435 |
| Ursodeoxycholate | rs312306 | C | G | -0.065 | | 0.015 | 6.23E-06 | | 0.045 | 0.035 | 0.205 | 20.343 |
| Ursodeoxycholate | rs3760877 | T | C | 0.058 | | 0.013 | 9.55E-06 | | -0.005 | 0.029 | 0.853 | 19.700 |
| Ursodeoxycholate | rs6763802 | T | G | -0.058 | | 0.012 | 6.50E-07 | | 0.038 | 0.062 | 0.535 | 24.656 |
| Ursodeoxycholate | rs7463380 | C | G | 0.076 | | 0.017 | 6.40E-06 | | -0.027 | 0.039 | 0.491 | 20.383 |
| Ursodeoxycholate | rs9573265 | T | C | 0.222 | | 0.049 | 5.55E-06 | | -0.081 | 0.058 | 0.165 | 20.621 |
