# Supplementary material for: Investigating causal associations among gut microbiota, metabolites, and liver diseases: a Mendelian randomization study
Source: Front Endocrinol (Lausanne). 2023 Jul 5;14:1159148. doi: 10.3389/fendo.2023.1159148 (PMC10354516; doi:10.3389/fendo.2023.1159148)
Supplement: Supplementary file 12 [file Table_12.docx]

| Table S12. SNPs used as instrumental variables from gut microbiota-derived metabolites and their associations with viral hepatitis | | | | | | | | | | | | |
| --- | --- | --- | --- | --- | --- | --- | --- | --- | --- | --- | --- | --- |
| Metabolites | SNP | Effect allele | Other allele | | metabolites | | | | viral hepatitis | | | F |
|  |  |  |  |  | Beta | SE | *p* value |  | Beta | SE | *p* value |  |
| Alanine | rs10211524 | A | G | -0.010 | | 0.002 | 7.99E-06 | | -0.047 | 0.033 | 0.155 | 20.446 |
| Alanine | rs11183620 | A | G | 0.010 | | 0.002 | 1.55E-08 | | 0.041 | 0.034 | 0.223 | 33.232 |
| Alanine | rs11652554 | T | C | 0.009 | | 0.002 | 1.52E-06 | | -0.003 | 0.033 | 0.915 | 23.361 |
| Alanine | rs11704957 | A | C | -0.023 | | 0.005 | 2.85E-06 | | 0.034 | 0.084 | 0.691 | 22.224 |
| Alanine | rs11893991 | A | G | 0.009 | | 0.002 | 8.89E-07 | | 0.012 | 0.033 | 0.711 | 24.448 |
| Alanine | rs1260326 | T | C | 0.013 | | 0.002 | 5.56E-14 | | 0.003 | 0.034 | 0.923 | 59.381 |
| Alanine | rs1289671 | A | G | -0.008 | | 0.002 | 1.56E-06 | | -0.016 | 0.032 | 0.620 | 21.778 |
| Alanine | rs1376492 | A | T | 0.009 | | 0.002 | 3.59E-07 | | -0.039 | 0.034 | 0.247 | 25.000 |
| Alanine | rs1440327 | T | C | -0.008 | | 0.002 | 6.00E-06 | | -0.046 | 0.032 | 0.159 | 20.753 |
| Alanine | rs1566951 | T | C | -0.008 | | 0.002 | 4.52E-06 | | 0.004 | 0.033 | 0.904 | 20.753 |
| Alanine | rs17106647 | T | C | 0.028 | | 0.006 | 8.85E-07 | | 0.005 | 0.120 | 0.968 | 24.476 |
| Alanine | rs17520130 | T | C | -0.009 | | 0.002 | 6.85E-07 | | -0.015 | 0.033 | 0.652 | 23.901 |
| Alanine | rs1850267 | T | C | 0.008 | | 0.002 | 9.56E-06 | | 0.051 | 0.032 | 0.115 | 19.262 |
| Alanine | rs2064272 | A | G | -0.008 | | 0.002 | 4.49E-06 | | 0.014 | 0.036 | 0.697 | 20.753 |
| Alanine | rs211962 | T | C | -0.012 | | 0.003 | 5.31E-06 | | -0.021 | 0.032 | 0.514 | 21.302 |
| Alanine | rs2295689 | A | G | -0.009 | | 0.002 | 6.80E-06 | | -0.067 | 0.033 | 0.041 | 20.250 |
| Alanine | rs4317609 | A | G | -0.008 | | 0.002 | 7.98E-06 | | -0.019 | 0.034 | 0.589 | 19.262 |
| Alanine | rs465152 | A | G | -0.009 | | 0.002 | 6.45E-07 | | -0.023 | 0.033 | 0.486 | 26.190 |
| Alanine | rs485878 | A | G | -0.008 | | 0.002 | 4.67E-06 | | 0.034 | 0.033 | 0.291 | 20.250 |
| Alanine | rs4980554 | A | G | 0.008 | | 0.002 | 2.87E-06 | | -0.013 | 0.033 | 0.685 | 23.266 |
| Alanine | rs6053126 | A | G | -0.014 | | 0.003 | 3.06E-06 | | -0.020 | 0.034 | 0.564 | 21.778 |
| Alanine | rs6461542 | A | T | -0.008 | | 0.002 | 6.95E-06 | | -0.034 | 0.033 | 0.299 | 19.262 |
| Alanine | rs6497167 | T | G | 0.008 | | 0.002 | 7.64E-06 | | -0.022 | 0.034 | 0.511 | 19.262 |
| Alanine | rs651158 | A | G | 0.009 | | 0.002 | 8.12E-07 | | 0.003 | 0.033 | 0.917 | 23.361 |
| Alanine | rs6706043 | T | G | -0.008 | | 0.002 | 7.33E-06 | | -0.060 | 0.032 | 0.061 | 19.262 |
| Alanine | rs6839120 | A | G | 0.008 | | 0.002 | 4.03E-06 | | 0.049 | 0.034 | 0.154 | 20.753 |
| Alanine | rs6962350 | T | G | 0.008 | | 0.002 | 5.68E-06 | | -0.033 | 0.034 | 0.332 | 19.753 |
| Alanine | rs703462 | C | G | 0.009 | | 0.002 | 1.06E-07 | | -0.013 | 0.037 | 0.735 | 26.694 |
| Alanine | rs7191435 | T | C | 0.008 | | 0.002 | 2.53E-06 | | 0.022 | 0.032 | 0.496 | 21.778 |
| Alanine | rs7712111 | A | G | 0.008 | | 0.002 | 1.63E-06 | | -0.052 | 0.033 | 0.111 | 21.778 |
| Alanine | rs7791060 | A | G | -0.017 | | 0.003 | 6.96E-07 | | 0.086 | 0.095 | 0.363 | 24.125 |
| Alanine | rs785896 | A | G | 0.009 | | 0.002 | 6.80E-06 | | 0.072 | 0.032 | 0.026 | 20.250 |
| Alanine | rs890230 | T | C | 0.009 | | 0.002 | 1.06E-06 | | 0.013 | 0.032 | 0.695 | 22.827 |
| Alanine | rs9367164 | A | G | 0.008 | | 0.002 | 9.17E-06 | | -0.012 | 0.034 | 0.710 | 20.516 |
| Alanine | rs9582849 | A | G | 0.020 | | 0.004 | 1.34E-06 | | -0.004 | 0.038 | 0.925 | 23.795 |
| Alanine | rs9866434 | A | T | 0.008 | | 0.002 | 5.55E-06 | | 0.027 | 0.033 | 0.410 | 19.753 |
| Alanine | rs9876174 | T | C | 0.012 | | 0.003 | 7.00E-06 | | 0.016 | 0.049 | 0.740 | 20.250 |
| Cholate | rs10999482 | T | G | -0.050 | | 0.011 | 7.28E-06 | | -0.074 | 0.036 | 0.040 | 20.332 |
| Cholate | rs1523437 | A | G | -0.062 | | 0.013 | 2.75E-06 | | -0.058 | 0.047 | 0.221 | 21.990 |
| Cholate | rs16823145 | A | G | -0.261 | | 0.059 | 8.58E-06 | | -0.188 | 0.057 | 0.001 | 19.831 |
| Cholate | rs17723514 | A | G | -0.048 | | 0.010 | 2.30E-06 | | 0.040 | 0.034 | 0.232 | 22.238 |
| Cholate | rs1868259 | A | G | 0.082 | | 0.018 | 7.67E-06 | | -0.005 | 0.032 | 0.870 | 19.980 |
| Cholate | rs2689327 | A | G | 0.049 | | 0.011 | 7.74E-06 | | -0.009 | 0.038 | 0.807 | 20.005 |
| Cholate | rs310271 | A | G | -0.053 | | 0.012 | 5.53E-06 | | -0.007 | 0.036 | 0.841 | 20.753 |
| Cholate | rs6595543 | A | G | 0.074 | | 0.017 | 7.19E-06 | | -0.014 | 0.032 | 0.661 | 20.114 |
| Cholate | rs9510021 | A | C | -0.047 | | 0.010 | 5.77E-06 | | -0.074 | 0.032 | 0.022 | 20.645 |
| Threonate | rs10097949 | A | G | 0.048 | | 0.010 | 3.41E-06 | | 0.013 | 0.059 | 0.828 | 21.686 |
| Threonate | rs10512185 | T | C | 0.017 | | 0.004 | 3.32E-06 | | -0.021 | 0.034 | 0.544 | 21.262 |
| Threonate | rs11914582 | T | C | -0.031 | | 0.007 | 2.89E-06 | | 0.022 | 0.034 | 0.517 | 21.637 |
| Threonate | rs12447958 | A | G | 0.019 | | 0.004 | 7.57E-06 | | -0.041 | 0.034 | 0.234 | 20.036 |
| Threonate | rs12779976 | A | G | 0.035 | | 0.007 | 1.26E-06 | | 0.052 | 0.061 | 0.395 | 23.227 |
| Threonate | rs13163920 | T | C | 0.016 | | 0.004 | 9.51E-06 | | 0.021 | 0.034 | 0.543 | 19.753 |
| Threonate | rs2257061 | T | C | -0.016 | | 0.004 | 6.33E-06 | | 0.015 | 0.034 | 0.660 | 20.250 |
| Threonate | rs2303010 | T | C | -0.147 | | 0.031 | 2.26E-06 | | -0.011 | 0.094 | 0.911 | 22.372 |
| Threonate | rs306472 | T | C | 0.020 | | 0.004 | 6.69E-06 | | -0.034 | 0.043 | 0.428 | 20.046 |
| Threonate | rs3766606 | T | G | 0.021 | | 0.005 | 4.93E-06 | | 0.009 | 0.036 | 0.814 | 21.160 |
| Threonate | rs576266 | T | C | -0.028 | | 0.006 | 5.97E-06 | | -0.011 | 0.042 | 0.799 | 20.321 |
| Threonate | rs7132285 | T | C | 0.137 | | 0.030 | 6.12E-06 | | -0.073 | 0.056 | 0.187 | 20.473 |
| Threonate | rs7216075 | A | G | -0.022 | | 0.004 | 4.39E-07 | | 0.052 | 0.036 | 0.148 | 25.000 |
| Threonate | rs7332927 | A | G | 0.138 | | 0.030 | 3.37E-06 | | -0.217 | 0.114 | 0.057 | 21.569 |
| Threonate | rs7993666 | T | C | 0.021 | | 0.005 | 3.67E-06 | | -0.046 | 0.042 | 0.265 | 21.160 |
| Threonate | rs8031609 | T | G | 0.020 | | 0.004 | 7.45E-06 | | -0.036 | 0.041 | 0.387 | 20.250 |
| Threonate | rs892429 | A | G | 0.084 | | 0.019 | 9.86E-06 | | 0.016 | 0.057 | 0.779 | 19.499 |
| Threonate | rs9572687 | A | G | -0.016 | | 0.004 | 6.55E-06 | | 0.034 | 0.037 | 0.352 | 20.250 |
